# Triple aryne–tetrazine reaction enabling rapid access to a new class of polyaromatic heterocycles†
†Electronic supplementary information (ESI) available: Experimental protocols, characterization data, X-ray crystallographic data (CIF) and NMR spectra of all new compounds. CCDC 1400529. For ESI and crystallographic data in CIF or other electronic format see DOI: 10.1039/c5sc01726b



**DOI:** 10.1039/c5sc01726b

**Published:** 2015-07-03

**Authors:** Sung-Eun Suh, Stephanie A. Barros, David M. Chenoweth

**Affiliations:** a Department of Chemistry , University of Pennsylvania , 231 South 34th Street , Philadelphia , PA 19104 , USA . Email: dcheno@sas.upenn.edu

## Abstract

One of the most challenging goals of modern synthetic chemistry is to develop multi-step reactions for rapid and efficient access to complex molecules. We report a triple aryne–tetrazine reaction that enables rapid access to a new class of polyaromatic heterocycles. This new reaction, which couples diverse reactivity modes between simple aryne and tetrazine starting materials, proceeds in a single operation and takes less than 5 minutes in air with no metal catalyst.

## Abstract 2

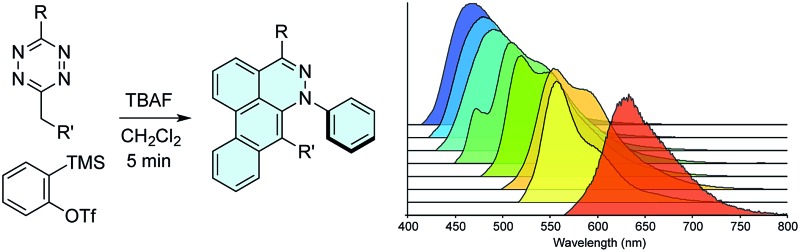
We report the triple aryne–tetrazine reaction for rapid access to a new class of dibenzocinnoline heteroaromatics.

## Introduction

**Fig. 1:**
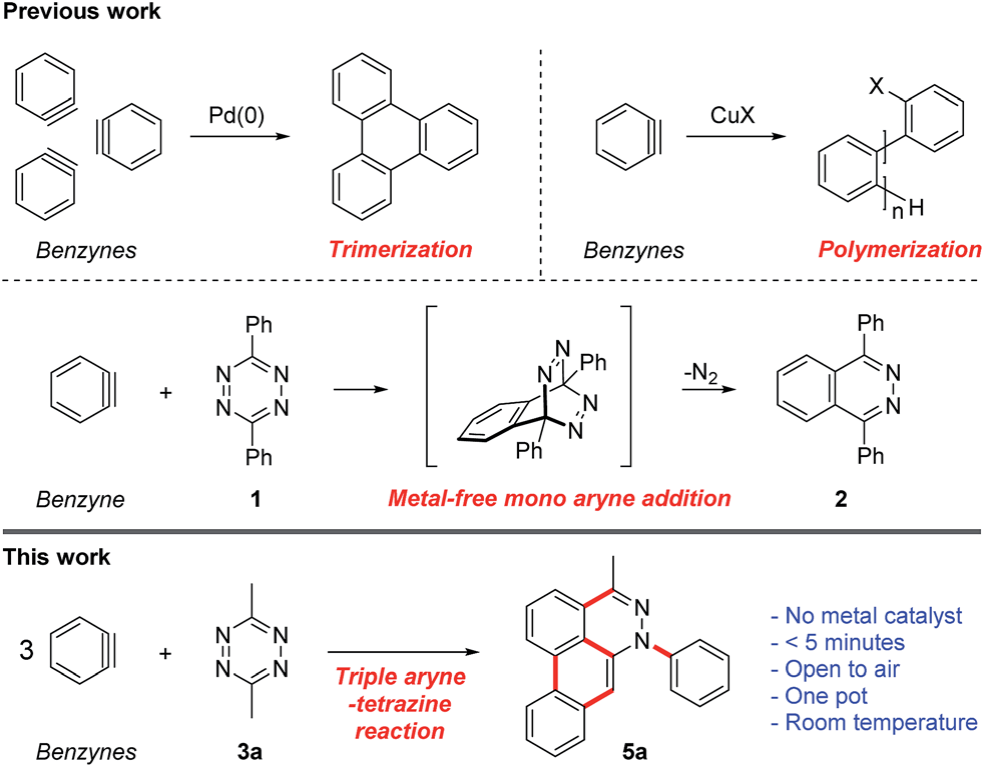
Triple aryne–tetrazine reaction. Red bold lines represent new bonds formed during the course of the reaction.

New polyaromatic heterocycles are important for the discovery and development of lead bioactive molecules and chemical probes. Our interest in developing new chemical probes and selective nucleic acid modulators motivated us to explore direct methods for rapidly accessing novel polyaromatic systems.^1,2^ Recent advances in benzyne/aryne chemistry have paved the way for use of these reactive intermediates in new synthetic methods,^3–12^ complex natural product synthesis,^13–20^ and aryne polymerizations (Fig. 1).^21^ In principal, multiple-consecutive aryne additions to a central core molecule could provide a novel and efficient approach for rapid access to new polyaromatic systems. Metal catalyzed methods have been reported for cyclotrimerization^22^ and polymerization^21^ of arynes. However, harnessing the reactivity of arynes for controlled multiple-consecutive intermolecular reaction processes still remains a significant challenge. An alternative strategy for directly accessing higher order polyaromatic systems could be envisioned using arynes, such as benzyne, in combination with a reactive core molecule, such as a tetrazine, acting as a scaffold for iterative additions.

The reaction between benzyne and tetrazine **1** was reported approximately 50 years ago and is known to proceed through a Diels–Alder/retro-Diels–Alder process, resulting in phthalazine **2** as the terminal product (Fig. 1).^23^ However, if the phthalazine product is thought of as an intermediate, one could envision further aryne additions to produce polyaromatic systems through sequential aryne addition reactions. Here, we show that a simple change in the functionality on tetrazine (**3a**, Fig. 1) can unveil latent reactivity in the presence of arynes, providing rapid and direct access to a new class of dibenzo[*de*,*g*]cinnoline polyaromatic heterocycles **5a**. This new class of dibenzo[*de*,*g*]cinnoline exhibits pH responsiveness resulting in dramatic shifts in photophysical properties upon protonation. This new class of dibenzocinnolines may have several potential applications as cellular imaging agents or bioactive molecules.

## Results and discussion

The reaction transforming tetrazines into dibenzocinnolines is operationally simple and requires less than 5 minutes from start to finish. Recently, important new methods developed by Devaraj and co-workers have paved the way for the efficient synthesis of tetrazines, providing easy access to many substitution patterns.^24^ A source of fluoride anion serves as a mild reagent to rapidly initiate the reaction *via* desilylation of a masked benzyne precursor,^25,26^ obviating the necessity for a metal catalyst, external heat source, or inert gas. The reaction was performed in a vial, under air, charged with tetrazine and the benzyne precursor. After dropwise addition of a 1.0 M solution of tetrabutylammonium fluoride (TBAF) in THF at 24 °C, dibenzo[*de*,*g*]cinnoline **5a** was produced (Fig. 1). The structure of dibenzocinnoline **5a** was confirmed using X-ray crystallography (Table 2 and ESI†). Dibenzo[*de*,*g*]cinnolines, which have not been previously reported, share significant structural homology to several natural products.^27,28^

**Fig. 2:**
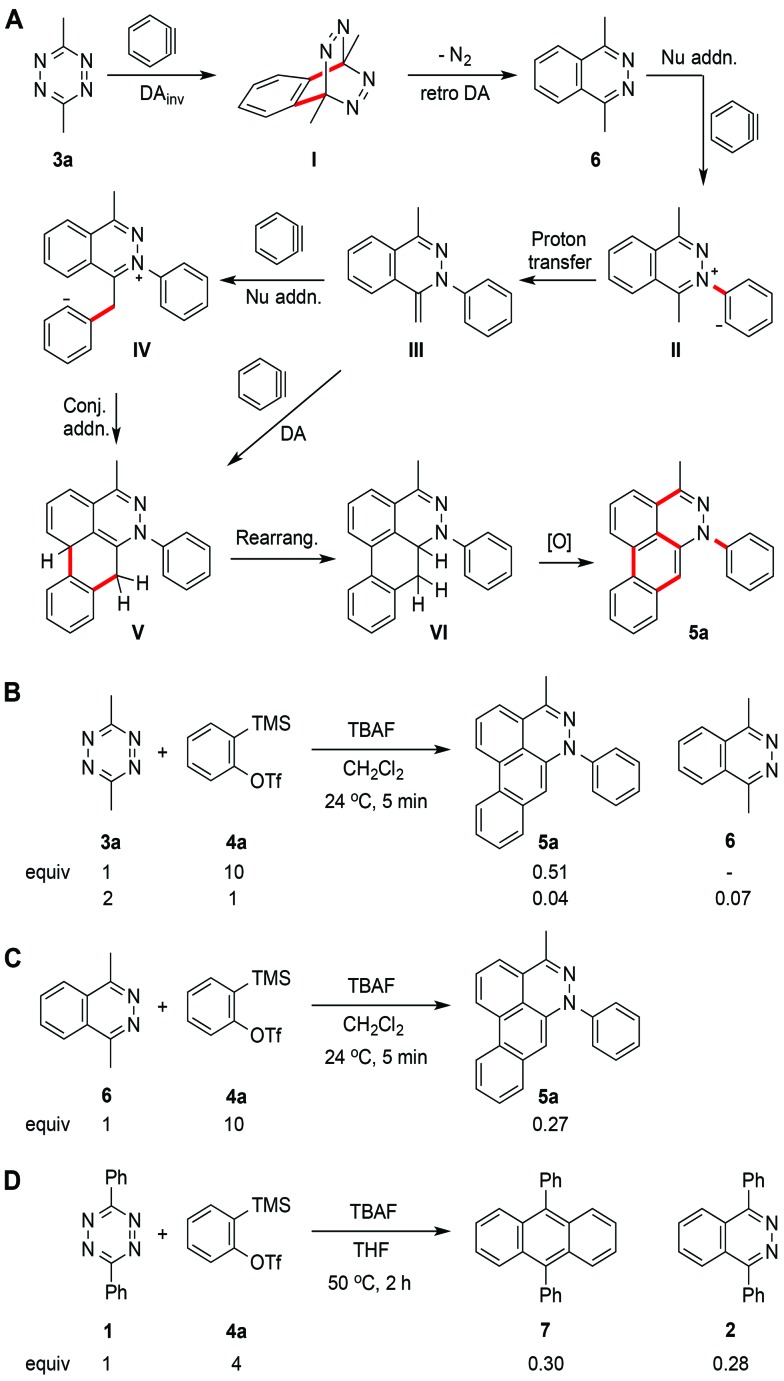
(A) Proposed mechanism of the reaction. DA, Diels-Alder; DA_inv_, inverse electron demand Diels-Alder. (B) Reaction conditions trapping intermediate **6**. (C) Resubjection of isolated intermediate **6** to the original reaction conditions. (D) Reaction of benzyne with diphenyltetrazine **1** to produce 9,10-diphenylanthracene **7**.

A plausible mechanism for the reaction proceeding through six elementary steps is shown in Fig. 2A. Aryne formation commences after addition of TBAF followed by formation of the initial [4 + 2] bicyclo intermediate **I** from **3a**. Cycloreversion with concomitant loss of nitrogen gas produces intermediate phthalazine heterocycle **6**.^23^ Next, phthalazine **6** undergoes nucleophilic addition to benzyne, affording key intermediate **II** followed by proton transfer to produce *s-cis* diene intermediate **III**. A third aryne engages the newly formed diene through one of two possible mechanisms. This first possibility is a second [4 + 2] cycloaddition reaction, resulting in dihydrophenanthrene-like intermediate **V**. The alternate possibility is a non-concerted pathway where a third equivalent of benzyne is attacked by the nucleophilic enamine like intermediate **III** to produce iminium **IV**, followed by intramolecular conjugated addition to afford **V**.^29^ Two mechanistic possibilities are envisioned for the final oxidation, where the first mechanism involves a direct 1,4-oxidation to yield **5a**. Although this mechanism is plausible, we favor a second possible pathway in which a base, such as fluoride, hydroxide, or water,^11^ facilitates an initial 1,4- to 1,2-dihydro rearrangement of cross-conjugated intermediate **V** to restore aromaticity in the [*de*] ring leading to intermediate **VI**. This step is then followed by either direct oxidation or aryne promoted desaturation leading to the fully aromatized dibenzocinnoline **5a**. The feasibility of the aryne promoted desaturation process was recently demonstrated in seminal studies by Hoye and coworkers.^12^ To test the aryne desaturation pathway, we conducted the tandem reaction in the absence of air. The heterocyclic product was formed, providing support for the desaturation pathway.

To trap intermediate phthalazine **6**, 2 equivalents of **3a** were used with 1 equivalent of benzyne precursor **4a** (Fig. 2B), since **6** was not observed in the optimized conditions shown in Table 1, entry 7. Using these conditions, **6** was obtained in 7% yield and was resubjected to the optimized reaction conditions (Fig. 2B and C). The desired product **5a** was produced in 27% yield. Although further inverse Diels–Alder reaction between intermediate **6** and a benzyne is plausible, 9,10-dimethylanthracene was not observed. However, when diphenyltetrazine **1** reacted with benzyne precursor **4a** and TBAF in THF at 50 °C for 2 hours, 9,10-diphenylanthracene **7** and 1,4-diphenylphthalazine **2** were both obtained. This result was consistent with our hypothesis that a benzylic proton unveils masked reactivity at phthalazines diverting the reaction down alternate competing mechanistic pathways leading to the formation of dibenzo[*de*,*g*]cinnolines.

**Table 1:** Selected optimization experiments of the reaction

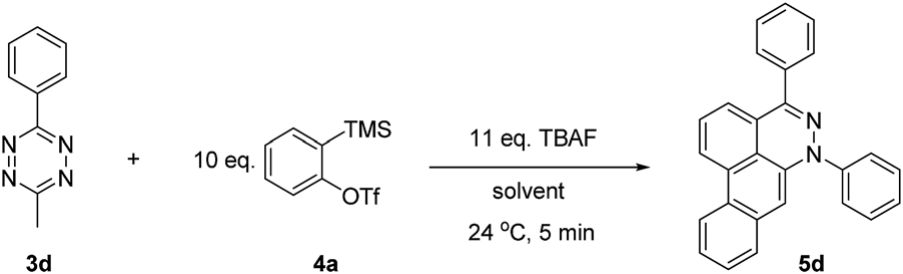			
Entry	Solvent	Conc. (M)	Yield^*a*^ (%)
1	THF	1	22
2	Et_2_O	1	25
3	Hexanes	1	14
4	Xylenes	1	14
5	DMF	1	1
6	CH_3_CN	1	25
7	CH_2_Cl_2_	1	26
8	CH_2_Cl_2_	0.1	6
9	CH_2_Cl_2_	0.5	7
10	CH_2_Cl_2_	1.5	10
11	CH_2_Cl_2_	2.0	13
12	—	—	19

^*a*^HPLC yield. 9,10-Diphenylanthracene was used as an internal standard.

Reaction optimization studies were conducted using nonvolatile tetrazine **3d** (Table 1). Among the several desilylating reagents used (see Table S1 in ESI†), TBAF afforded **5d** in 26% yield (Table S1,† entry 1), while other fluoride anion sources, such as tetrabutylammonium difluorotriphenylsilicate (TBAT), CsF, HF, and KF, resulted in no reaction (Table S1,† entries 2–12). In the presence of 18-crown-6, KF resulted in a low yield of product **5d** (Table S1,† entry 13). As expected, the reaction did not occur when other additives including tetrabutylammonium chloride or tetrabutylammonium bromide were added in the absence of a fluoride source (Table S2 in ESI†).

The highest yields were obtained using CH_2_Cl_2_ as the solvent. However, several alternative solvents such as THF, Et_2_O, xylene, hexane, and CH_3_CN resulted in a reasonable yield of product **5d** (Table 1, entries 1–7). The reaction was sensitive to the concentration with the highest conversion to product achieved at a final concentration of 1.0 M (Table 1, entries 7–12). In addition, excess benzyne precursor and TBAF reagents were required to achieve optimal yields (see Table S3 in ESI†). A decrease in the temperature and an increase in the reaction time resulted in decreased product yields (see Table S4 in ESI†). The addition of TBAF to the reaction mixture over the course of 60 seconds resulted in the best yields. During the TBAF addition, a steady increase in the internal temperature of the reaction mixture to a maximum of 51 °C after 70 seconds followed by a decrease to 24 °C was observed. After a total time of 300 seconds, the reaction was complete (see Fig. S1 in ESI†).

Interestingly, **5d** was isolated in 27% yield when **4a** was used as a limiting reagent, TBAF was added to **3d** over a period of 4 hours at 0 °C, and the solution was stirred for an additional 20 hours (see Table S4,† entry 7). Reduction in the amount of benzyne precursor used resulted in longer reaction times with unreacted tetrazine and required lower temperatures. Further optimization is currently underway in our group.

Using the optimized conditions described here, several dibenzocinnolines **5a–5j** were synthesized. Given that the reaction forms four new C–C bonds and one new C–N bond during the course of 6–8 steps, yields from 10–52% are quite reasonable. Alkyl, aryl, and heteroaryl substituents are tolerated in position R1. Differentially dialkylated tetrazines show selectivity for reaction at the least sterically hindered position. For example, 3-methyl-6-nonyl-1,2,4,5-tetrazine **3b** has two benzylic positions; however, only product **5b** (Table 2) was observed, consistent with nonyl group sterics dictating addition of the second benzyne to the least sterically hindered nitrogen atom (see intermediate **II** in Fig. 2), which is much bulkier than the methyl group. In addition, isolated **5a** or **5b** did not react further with excess benzyne in CH_2_Cl_2_ or THF under refluxing conditions. This result is consistent with the nitrogen–benzyne adduct creating a sterically congested environment that precludes addition to the adjacent nitrogen atom, as seen from the crystal structure of **5a** in Table 2. To further test the influence of steric hindrance, the bulky adamantyl tetrazine **3c** was used. The bulky adamantyl group was expected to shut down the Diels–Alder reaction, preventing appropriate alignment of the aryne with the central tetrazine carbon atoms. To our surprise, product **5c** was produced in 10% yield.

**Table 2:** Substrate scope for the synthesis of dibenzo[*de*,*g*]cinnolines and X-ray crystal structure of **5a** ^*a*^ ^,^ ^*b*^

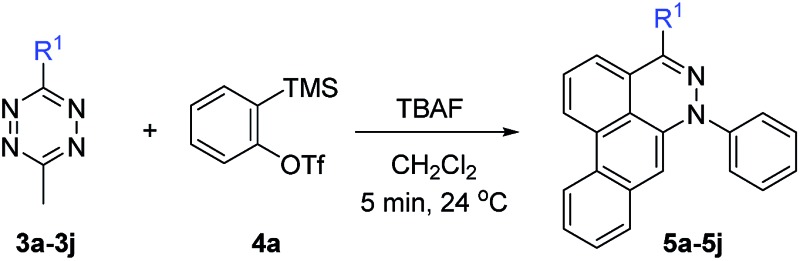
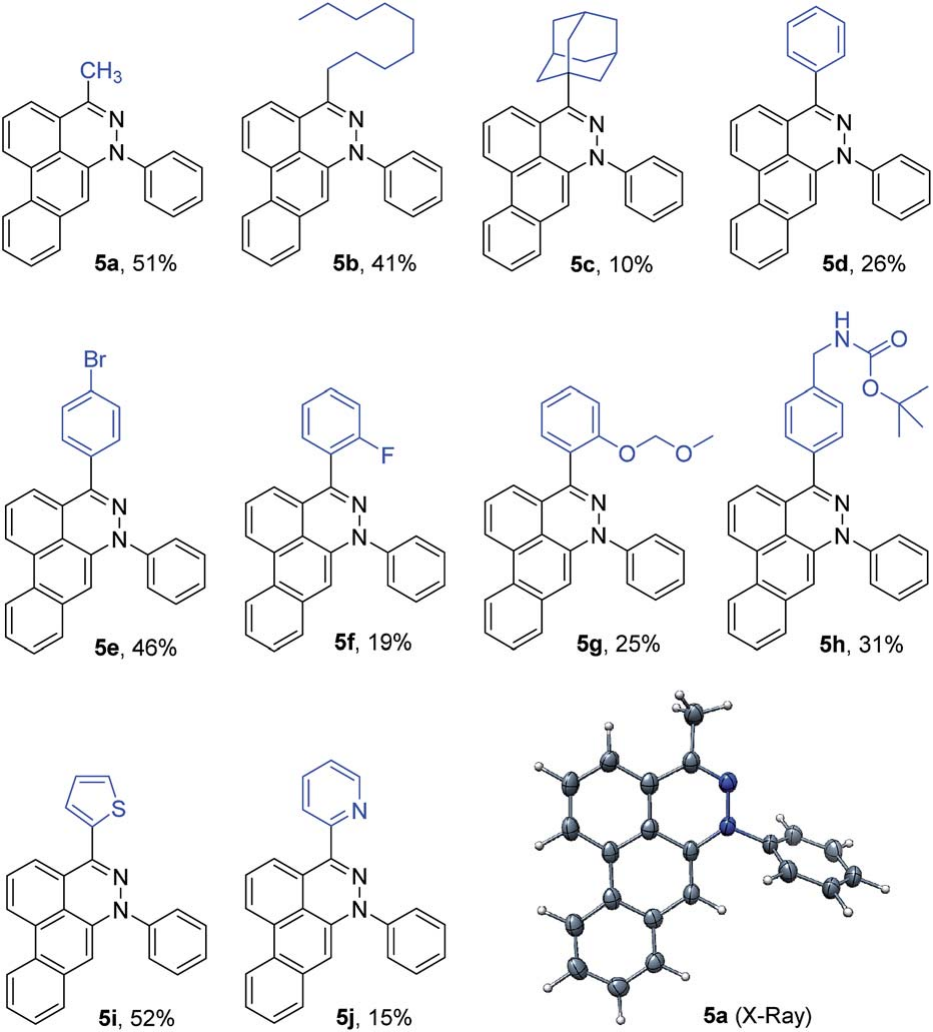

^*a*^Reactions were performed under the optimized condition shown in Table 1, entry 7.

^*b*^Isolated yield.

To broaden the scope of the reaction, difunctionalized tetrazines were explored. We found that the reaction is not limited to substitution at the R1 position. Difunctionalized tetrazines with substitution at R2 resulted in reasonable yields of dibenzocinnoline products **5k–5m** (Table 3). Encouraged by these results, alternate benzyne precursors were tested with a set of tetrazines (Table 4). We found that addition of 4,5-dimethyl-*o*-benzyne to tetrazines afforded **5n–5p** in reasonable yields (24–31%; Table 4).

**Table 3:** Scope of tetrazines for the synthesis of dibenzo[*de*,*g*]cinnolines^*a*^ ^,^ ^*b*^

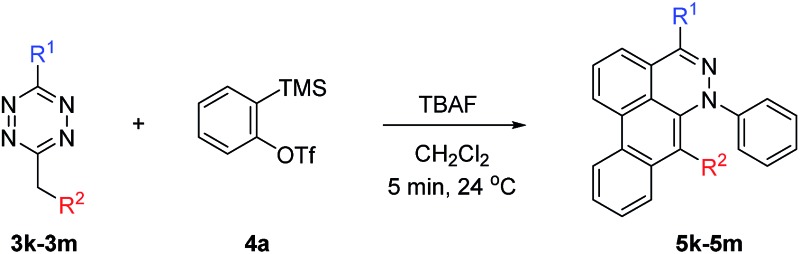
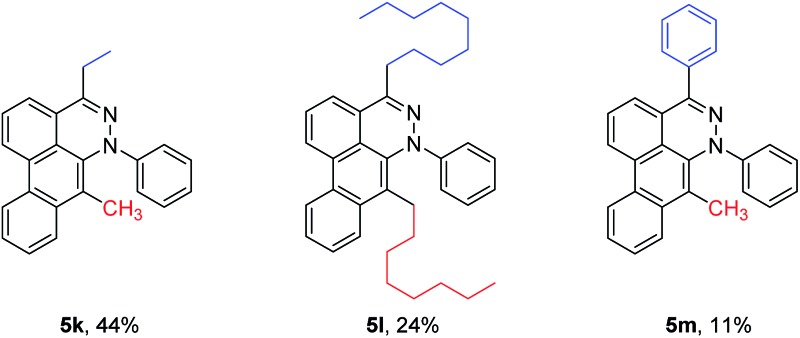

^*a*^Reactions were performed under the optimized conditions shown in Table 1, entry 7.

^*b*^Isolated yield.

**Table 4:** Addition of 4,5-dimethyl-*o*-benzyne^*a*^ ^,^ ^*b*^

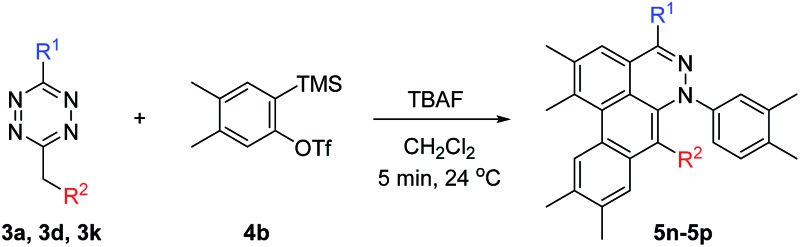
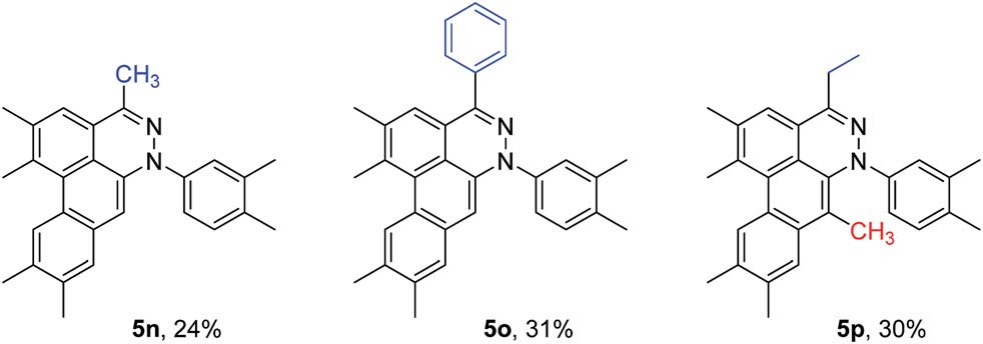

^*a*^Reactions were performed under the optimized condition shown in Table 1, entry 7.

^*b*^Isolated yield reported.

Depending on the tetrazine used, we found that TBAF not only initiates the reaction but also decomposes the tetrazine starting materials in a competing but slower background reaction. For mono-substituted tetrazines, such as 3-methyl-1,2,4,5-tetrazine, decomposition was faster than the initial Diels–Alder reaction, resulting in neither the desired product nor the phthalazine intermediate. Additionally, 3-benzyl-6-methyl-1,2,4,5-tetrazine decomposed faster due to the increased acidity of the methylene accelerating the tetrazine decomposition. Also, the addition of dinaphthyne precursor to 3-methyl-6-phenyl-1,2,4,5-tetrazine **3d** led to dinaphthocinnoline **5q**.

**Fig. 3:**
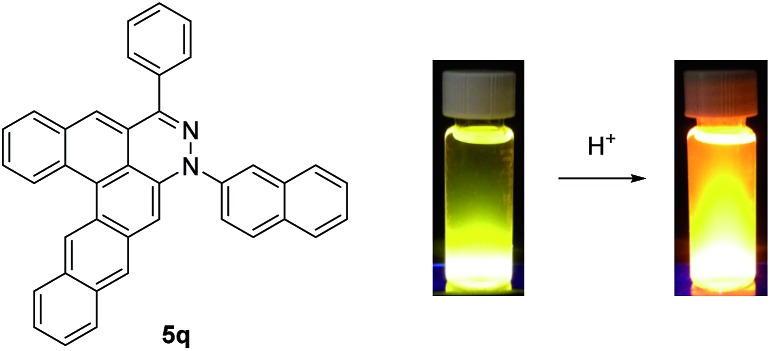
Structure of **5q**. Solutions of **5q** in CH_2_Cl_2_ (left) and in CH_2_Cl_2_/TFA (right) irradiated at 365 nm with a UV lamp (left).

**Fig. 4:**
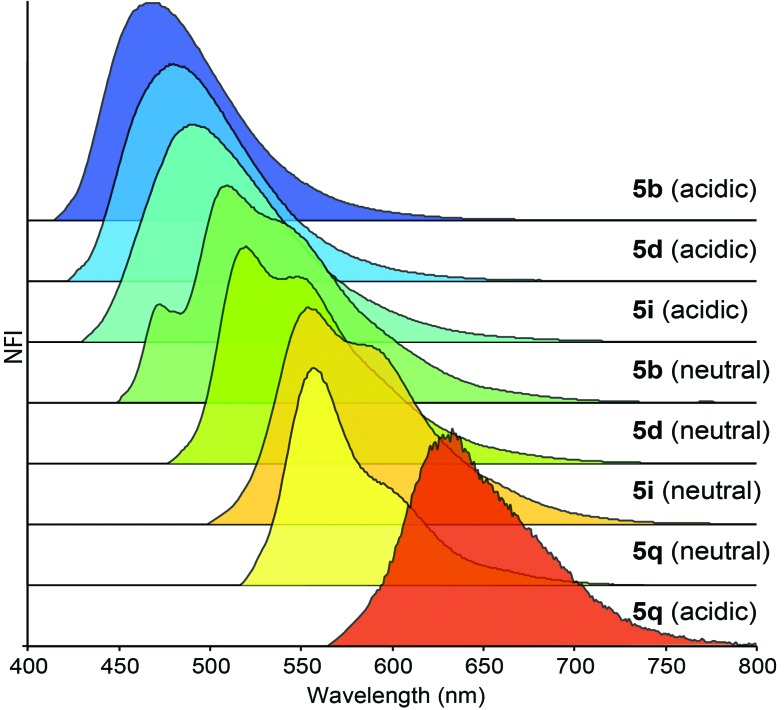
Emission spectra for the neutral and protonated forms of compounds **5b**, **5d**, **5i**, and **5q**.

**Fig. 5:**
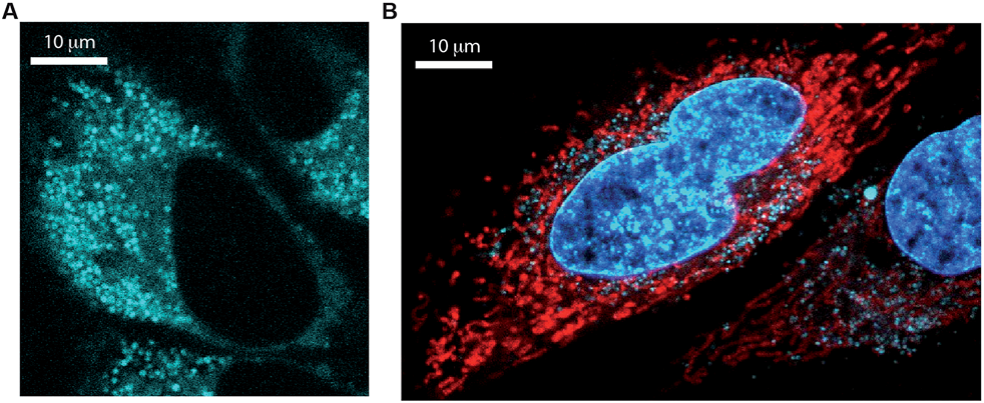
Live-cell imaging of HeLa cells in the presence of **5e**. (A) Cultured HeLa cells were incubated with **5e** for 2 hours and imaged using confocal microscopy. (B) Cells were incubated with **5e** for 2 hours, then counterstained with Hoechst 33342 and MitoTracker Red FM. Extranuclear cyan corresponds to compound **5e**. Using an excitation of 405 nm and emission from 450 to 500 nm allows for simultaneous visualization of **5e**, nuclear localized Hoechst 33342, and mitochondrial localized MitoTracker Red FM.

Dibenzo[*de*,*g*]cinnolines exhibit interesting photophysical properties (see ESI†). Dibenzo[*de*,*g*]cinnolines **5b**, **5d**, and **5i** are emissive in the solid state and solution state under 365 nm UV radiation. Additionally they exhibited hypsochromic emission response in the presence of acid. In contrast, dinaphthocinnoline **5q** exhibited a bathochromic emission shift in the presence of acid unlike **5b**, **5d**, and **5i** (Fig. 3). Taken together, these results show that the photophysical properties are tunable across a large window of the visible spectrum (Fig. 4) and we expect even more interesting photophysical phenomena as we explore new derivatives for applications in materials, sensing, and imaging. Preliminary cellular imaging with **5e** shows that upon excitation of the acidic form at 405 nm, intracellular vesicles can be selectively stained (Fig. 5a). When paired with commonly used dyes, such as Hoechst and MitoTracker, we have the ability to differentiate different intracellular compartments (Fig. 5b). The ability to tune the spectral properties of the dibenzocinnolines for selective imaging of subcellular structures makes this a promising new class of fluorescent probes for live cell imaging.

## Conclusions

In summary, we have developed a new synthetic approach to rapidly access a structurally novel class of dibenzo[*de*,*g*]cinnolines using tetrazine and aryne precursors. The starting materials are easily prepared, and the reaction is simple and facile, proceeding in less than 5 minutes. The photophysical properties of this new class of fluorophore are tunable and compatible with live-cell imaging. Efforts are currently underway to further probe the biological activity and to expand access to different structures *via* regioselective asymmetric aryne addition.^30^
