# Supplementary material for: Triple aryne–tetrazine reaction enabling rapid access to a new class of polyaromatic heterocycles
Source: Chem Sci. 2015 Jul 3;6(9):5128–32. doi: 10.1039/c5sc01726b (PMC4570739; doi:10.1039/c5sc01726b)
Supplement: Supplementary file 1 [file SC-006-C5SC01726B-s001.pdf]

Electronic Supplementary Material (ESI) for Chemical Science.
This journal is © The Royal Society of Chemistry 2015

Triple Aryne-Tetrazine Reaction Enabling Rapid Access to a New Class of Polyaromatic Heterocycles

Sung-Eun Suh, Stephanie A. Barros, and David M. Chenoweth*

Department of Chemistry, University of Pennsylvania, 231 34th Street,

Philadelphia, Pennsylvania 19104-6323, United States

Table of content

General information …………………………………………………………… S02

Additional optimization information ……………………………………………… S03 – S04

Real-time temperature study…………………………………………………… S05

Experimental procedures ……………………………………………………… S06 – S37

References ……………………………………………………………………… S38

NMR spectra …………………………………………………………………… S39 – S114

UV/Vis and fluorescence spectra ………………………………………………… S115 – S121

Crystal data and structure refinement…………………………………………… S122 – S123

General information

Materials. All commercial reagents and solvents were used as received. Zinc trifluoromethanesulfonate, hydrazine anhydrous, propionitrile, decanenitrile, 2-thiophenecarbonitrile, 2-pyridinecarbonitrile, 2-fluorobenzonitrile, adamantane-1-carbonitrile, 4-(aminomethyl)benzonitrile hydrochloride, di-*tert*-butyl dicarbonate and 1.0M tetrabutylammonium fluoride in THF were purchased from Aldrich. Acetonitrile and benzonitrile were purchased from Fisher Scientific, 4-bromobenzonitrile from Oakwood Products Inc, 3-(Trimethylsilyl)-2-naphthyl trifluoromethanesulfonate from TCI and methylene chloride-d_2 and chloroform-d from Cambridge Isotope Laboratories Inc. Flash column chromatography was performed using Silicycle silica gel (55−65 Å pore diameter). Thin-layer chromatography was performed on Sorbent Technologies silica plates (250 μm thickness). Dulbecco's Modified Eagle Medium (DMEM) was purchased from Invitrogen. Fetal bovine serum (FBS) was purchased from Gibco, Life Technologies. L-glutamine and penicillin/streptomycin were purchased from Corning Cellgro.

General spectroscopic methods. Proton nuclear magnetic resonance spectroscopy (^{1}H NMR) and Carbon nuclear magnetic resonance spectroscopy (^{13}C NMR) spectra were recorded on a Bruker UNI 500 NMR. Fluorine nuclear magnetic resonance spectroscopy (^{19}F NMR) was recorded on a Bruker DMX 360 NMR. High-resolution mass spectra were obtained by Dr. Rakesh Kohli at the University of Pennsylvania's Mass Spectrometry Service Center on a Micromass AutoSpec electrospray/chemical ionization spectrometer. X-ray diffraction data obtained and solved by Dr. Patrick Carroll at the University of Pennsylvania. Ultraviolet absorption spectrophotometry was performed on a JASCO V-650 spectrophotometer with a PAC-743R multichannel Peltier using quartz cells with a 1 cm cell path length. High performance liquid chromatography analysis was performed using a Jasco HPLC instrument equipped with a Phenomenex column (Luna 5u C18(2) 100A; 250 × 4.60 mm, 5 μm). Graph of time versus temperature was obtained using a Vernier Go-Link and temperature probe.

Cell Culture and Imaging. HeLa cells were maintained in a humidified incubator at 37 °C in 5% CO_2. HeLa cells were cultured in DMEM supplemented with 10% fetal bovine serum (Giboc, Life Technologies) and penicillin and streptomycin (Corning Cellgro). For live cell imaging, cells were plated in glass-bottom 35-mm dishes (MatTEK) 24 hours before experiments. 24 hours after plating, 1 μL of compounds (stock solution 10 mM) was added to 2 mL of media. Cells were incubated for 2 hours with compounds at 37 °C followed by washing with fresh media 2 times. During imaging, cells were maintained in DMEM without phenol red supplemented with 10% FBS. A Leica TCS SP8 confocal microscope equipped with a 63x/1.4 NA oil immersion objective lens was used. Compound **5e** was excited at 405 nm and emission was observed from 450-500 nm. To observed MitoTracker Red FM, an excitation of wavelength of 552 nm was used and emission was observed from 575-640 nm. Hoechst 33342 was observed by excitation with 405 nm and emission from 408-450 nm. Images were processed using Fiji ImageJ.

Additional optimization information

The yield was determined by HPLC and 9,10-dipbhenylanthracene was used as an internal standard.

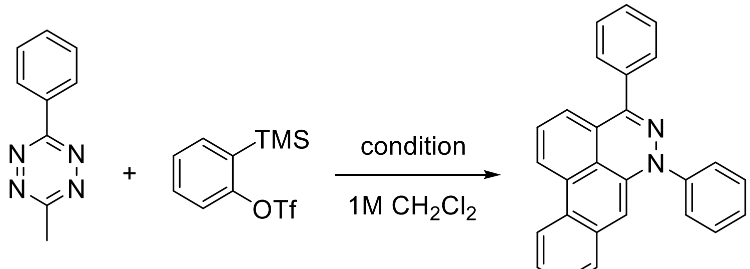

3d **4a** **5d**

Table S1. Effect of fluoride anion source and combination with additive.

Entry	F^-	Eq of F^-	Eq of Benz.	Additive[b]	Temp (°C)	Time	Yield (%)[c]
1	TBAF[a]	10	11	-	24	1 h	-
2	TBAF[a]	10	11	TFA	24	1 h	-
3	TBAF[a]	10	11	NEt_3	24	1 h	-
4	TBAF	11	10	-	24	5 min	26 (23 [d])
5	TBAT	11	10	-	24	1 h	-
6	TBAT	11	10	TFA	24	1 h	-
7	TBAT	11	10	NEt_3	24	1 h	-
8	CsF	11	10	-	24	1 h	-
9	CsF	11	10	TFA	24	1 h	-
10	CsF	11	10	NEt_3	24	1 h	-
11	HF	11	10	-	24	1 h	-
12	KF	11	10	-	24	1 h	-
13	KF	11	10	18-crown-6	24	5 min	3

[a] 1 eq of TBAF was added to 1.1 eq of benzyne precursor and then 10 eq of stock solution from those mixture was added to tetrazine.
[b] 10 eq of additive was added.
[c] HPLC yield.
[d] Isolated yield.

Table S2. Effect of additive without fluoride anion source.

Entry	F^-	Eq of F^-	Eq of Benz.	Additive[a]	Temp (°C)	Time	Yield (%)[b]
1	-	-	10	Indole	24	1 h	-
2	-	-	10	Imidazole	24	1 h	-
3	-	-	10	TMG	24	1 h	-
4	-	-	10	DBU	24	1 h	-
5	-	-	10	t-BuOK/THF	24	1 h	-
6	-	-	10	2,6-lutidine	24	1 h	-
7	-	-	10	Pyridine	24	1 h	-
8	-	-	10	*t*-BuNH$_2$	24	1 h	-
9	-	-	10	TBAOH	24	1 h	-
10	-	-	10	TBABr	24	1 h	-
11	-	-	10	TBACl	24	1 h	-

[a] 10 eq of additive was added.
[b] HPLC yield.

Table S3. Effect of equivalence of benzyne precursor and TBAF.

Entry	F^-	Eq of F^-	Eq of Benz.	Additive	Temp (°C)	Time	Yield (%)[a]
1	TBAF	11	10	-	24	5 min	26
2	TBAF	11	11	-	24	5 min	25
3	TBAF	8.8	8	-	24	5 min	19
4	TBAF	6.6	6	-	24	5 min	11
5	TBAF	4.4	4	-	24	5 min	6
6	TBAF	2.2	2	-	24	5 min	3
7	TBAF	1.1	1	-	24	5 min	1
8	TBAF	9	10	-	24	5 min	22
9	TBAF	7	10	-	24	5 min	14
10	TBAF	11	22	-	24	5 min	23

[a] HPLC yield.

Table S4. Effect of temperature and reaction time.

Entry	F^-	Eq of F^-	Eq of Benz.	Additive	Temp (°C)	Time	Yield (%)[a]
1	TBAF	11	10	-	24	5 min	26
2	TBAF	11	10	-	0	5 min	6
3	TBAF	11	10	-	0 to 24[b]	35 min	7
4	TBAF	11	10	-	0 to 24[b]	65 min	10
5	TBAF	11	10	-	24	65 min	13
6	TBAF	3.3	3	-	0	24 h[c]	27[d]
7	TBAF	3.3	3	-	24	4 h[e]	5[d]

[a] HPLC yield except entry 6.
[b] TBAF was added dropwise for 5 min at 0 °C and then the reaction mixture was warmed to 24 °C for additional time.
[C] TBAF was added in period of 4 h at 0 °C and then was stirred for additional 20 h.
[d] Isolated yield based on the benzyne precursor as the starting material.
[e] TBAF was added in period of 4 h at 24 °C.

Real-time temperature study

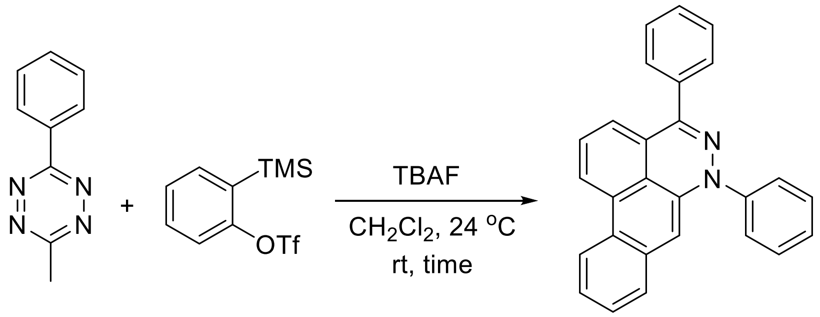

3d **4a** **5d**

A vial was charged with 14.8 mg (0.086 mmol) of **3d** and 300 mg (0.86 mmol) of 3-(trimethylsilyl)-2-naphthyl trifluoromethanesulfonate in 0.09 mL of dichloromethane at 24˚C, and 1 M TBAF in THF (0.95 mL, 0.95 mmol) was slowly added to the solution over the course of 1 min (0 to 60 sec in Supplementary Figure 1). The highest temperature was 52 ˚C at 73 sec.

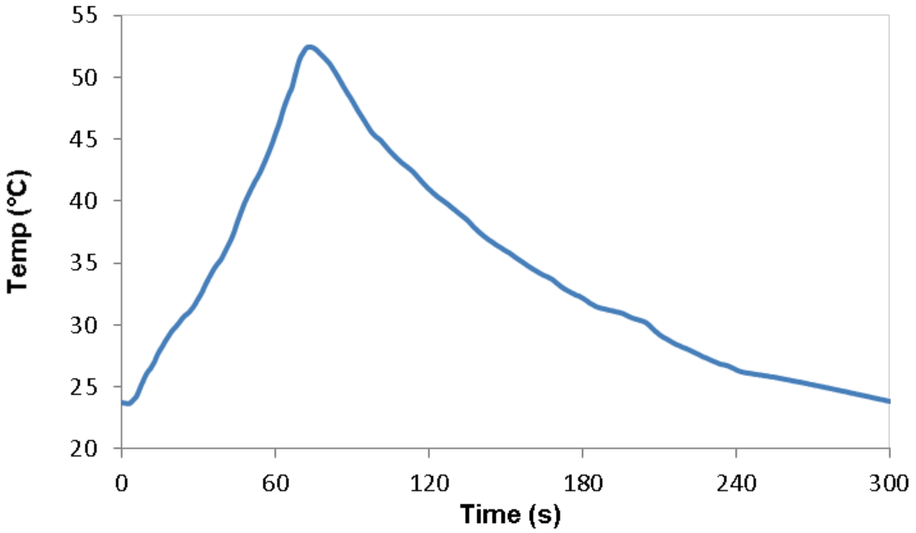

Figure S1. Graph of time vs. temperature of the reaction.

Experimental procedures

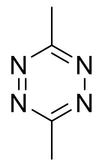

3a

3,6-dimethyl-1,2,4,5-tetrazine (3a): 3a was prepared according to the previously published procedure[1] from commercially available acetonitrile. The product was purified by flash column chromatography (ethyl acetate/hexane 1:4) to afford **3a**. The data for this compound was previously reported in the literature.[2-4]

Physical Property: Red crystal.

Isolated Yield: 30%

TLC: R_f = 0.29 (silica gel, ethyl acetate/hexane 1:4).

^{1}H NMR (500 MHz, $CDCl_3$) δ 2.96 (s, 6H).

^{13}C NMR (125 MHz, $CDCl_3$) δ 167.2, 21.0

IR (neat): 1636, 1412, 1350, 1292, 1270, 913, 873, and 734 cm^{-1}.

HRMS (ESI) calculated for $C_4H_7N_4$ $[M+H]^+$ 111.0660, no peak matched the calculated exact mass.[5]

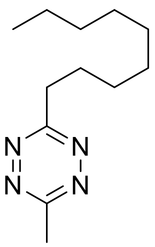

3b

3-methyl-6-nonyl-1,2,4,5-tetrazine (3b): 3b was prepared according to the previously published procedure[1] from commercially available acetonitrile and decanenitrile. The product was purified by flash column chromatography (ethyl acetate/hexane 1:16) to afford **3b**.

Physical Property: Purple crystal, m.p. = 33-34 ˚C.

Isolated Yield: 19%

TLC: $\mathbf{R_f}$ = 0.50 (silica gel, ethyl acetate/hexane 1:4).

^{1}**H NMR** (500 MHz, $CDCl_3$) δ 3.27 (t, 2H, J = 7.7 Hz), 3.02 (s, 3H), 1.96-1.88 (m, 2H), 1.46-1.19 (m, 12H), 0.90-0.84 (m, 3H).

^{13}**C NMR** (125 MHz, $CDCl_3$) δ 170.3, 167.4, 34.9, 32.0, 29.5, 29.39, 29.37, 29.27, 28.5, 22.8, 21.2, 14.2.

IR (neat): 2953, 2914, 2848, 2870, 1718, 1653, 1469, 1407, 1338, 1317, 1274, 1200, 891, 774, and 720 cm^{-1}.

HRMS (ESI) calculated for $C_{12}H_{23}N_4$ $[M+H]^+$ 223.1918, found 223.1922.

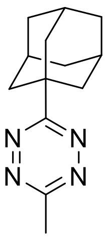

3c

3-((3r,5r,7r)-adamantan-1-yl)-6-methyl-1,2,4,5-tetrazine (3c): 3c was prepared according to the previously published procedure[1] from commercially available acetonitrile and adamantane-1-carbonitrile. The product was purified by flash column chromatography (ethyl acetate/hexane 1:12) to afford **3c**.

Physical Property: Red solid, m.p. = 55-56 ˚C.

Isolated Yield: 22%

TLC: $\mathbf{R_f}$ = 0.37 (silica gel, ethyl acetate/hexane 1:4).

^{1}H NMR (500 MHz, $CDCl_3$) δ 2.90 (s, 3H), 2.10-2.00 (m, 9H), 1.71 (bs, 6H).

^{13}C NMR (125 MHz, $CDCl_3$) δ 174.3, 166.6, 40.5, 39.2, 36.3, 28.2, 21.0.

IR (neat): 2905, 2850, 1453, 1398, 1353, 1312, 1088, 1046, 890, 736 cm^{-1}.

HRMS (ESI) calculated for $C_{13}H_{19}N_4$ $[M+H]^+$ 231.1605, found 231.1600.

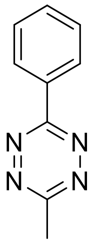

3d

3-methyl-6-phenyl-1,2,4,5-tetrazine (3d): 3d was prepared according to the previously published procedure[1] from commercially available acetonitrile and benzonitrile. The product was purified by flash column chromatography (ethyl acetate/hexane 1:10) to afford **3d**. The data for this compound was previously reported in the literature.[4,6]

Physical Property: Purple crystal.

Isolated Yield: 58%

TLC: $\mathbf{R_f}$ = 0.42 (silica gel, 25% ethyl acetate/hexane).

^{1}H NMR (500 MHz, $CDCl_3$) δ 8.51-8.45 (m, 2H), 7.56-7.46 (m, 3H), 3.01 (s, 3H).

^{13}C NMR (125 MHz, $CDCl_3$) δ 167.1, 163.9, 132.4, 131.8, 129.1, 127.8, 21.1.

IR (neat): 1473, 1401, 1362, 1176, 1089, 1022, 890, 760, 722, 692, and 675 cm^{-1}.

HRMS (ESI) calculated for $C_9H_9N_4$ $[M+H]^+$ 173.0822, found 173.0819.

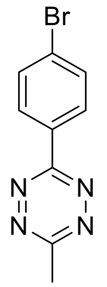

3e

3-(4-bromophenyl)-6-methyl-1,2,4,5-tetrazine (3e): 3e was prepared according to the previously published procedure[1] from commercially available acetonitrile and 4-bromobenzonitrile. The product was purified by flash column chromatography (ethyl acetate/hexane 1:10) to afford **3e**.

Physical Property: Purple crystal, m.p. = 151-152 ˚C.

Isolated Yield: 16%

TLC: R$_f$ = 0.53 (silica gel, ethyl acetate/hexane 1:4).

^{1}H NMR (500 MHz, $CDCl_3$) δ 8.43-8.38 (m, 2H), 7.70-7.66 (m, 2H), 3.07 (s, 3H)

^{13}C NMR (125 MHz, $CDCl_3$) δ 167.5, 163.6, 132.6, 130.8, 129.4, 127.7, 21.3.

IR (neat): 1588, 1402, 1366, 1174, 1091, 1072, 1008, 890, 847, 798, and 633 cm^{-1}.

HRMS (ESI) calculated for $C_9H_8BrN_4$ $[M+H]^+$ 250.9922, no peak matched the calculated exact mass.

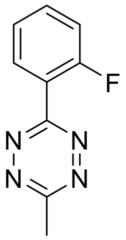

3f

3-(2-fluorophenyl)-6-methyl-1,2,4,5-tetrazine (3f): 3f was prepared according to the previously published procedure[1] from commercially available acetonitrile and 2-fluorobenzonitrile. The product was purified by flash column chromatography (ethyl acetate/hexane 1:8) to afford **3f**.

Physical Property: Red solid, m.p. = 50-51 ˚C.

Isolated Yield: 26%

TLC: $\mathbf{R_f}$ = 0.44 (silica gel, ethyl acetate/hexane 1:4).

^{1}H NMR (500 MHz, $CDCl_3$) δ 8.18 (td, 1H, J = 7.6, 1.8 Hz), 7.59-7.53 (m, 1H), 7.33 (td, 1H, J = 7.6, 1.0 Hz), 7.26 (dd, 1H, J = 10.8, 1.0 Hz), 3.08 (s, 3H).

^{13}C NMR (125 MHz, $CDCl_3$) δ 166.8, 163.9, 162.3, 160.2, 133.9, 133.8, 131.3, 124.74, 124.71, 120.7, 120.6, 117.32, 117.15, 21.2.

^{19}F NMR (338 MHz, $CDCl_3$) δ -112.8 to -112.9 (m, 1F).

IR (neat): 2129, 1614, 1586, 1497, 1466, 1400, 1364, 1253, 1216, 1114, 1087, 1028, 888, 821, 763, 736 cm^{-1}.

HRMS (ESI) calculated for $C_9H_8FN_4$ $[M+H]^+$ 191.0729, no peak matched the calculated exact mass.

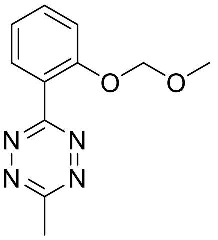

3g

3-(2-(methoxymethoxy)phenyl)-6-methyl-1,2,4,5-tetrazine (3g): A vial was charged with 80 mg (0.43 mmol) of **3n** and 0.44 mL (2.55 mmol) of *N*,*N*-diisopropylethylamine (DIPEA) in 1.00 mL of dichloromethane at 0 ˚C, and the solution was stirred for 20 min. To the resulting solution was added 0.01 mL (1.28 mmol) of chloromethylmethyl ether (MOMCl). After 20 min at 0 ˚C, the solution was warmed to 24 ˚C and stirred for another 12 h. The solution was quenched with saturated NH_4Cl solution, extracted with dichloromethane, and purified by column chromatography (ethyl acetate/hexane 1:4) to afford **3g** (54.1 mg).

Physical Property: Red oil.

Isolated Yield: 55%

TLC: $\mathbf{R_f}$ = 0.30 (silica gel, ethyl acetate/hexane 1:4).

^{1}H NMR (500 MHz, $CDCl_3$) δ 7.89 (dd, 1H, J = 7.7, 0.9 Hz), 7.53 (ddd, 1H, J = 8.4, 7.5, 1.7 Hz), 7.32 (d, 1H, J = 8.4 Hz), 7.20 (td, 1H, 7.5, 0.9 Hz), 5.23 (s, 2H), 3.47 (s, 3H), 3.10 (s, 3H).

^{13}C NMR (125 MHz, $CDCl_3$) δ 166.3, 165.9, 155.9, 132.9, 131.6, 123.3, 122.3, 116.1, 95.1, 56.4, 21.2.

IR (neat): 2923, 1603, 1496, 1461, 1399, 1154, 1080, 982, 756, 736 cm^{-1}.

HRMS (ESI) calculated for $C_{11}H_{13}N_4O_2$ $[M+H]^+$ 233.1034, found 233.1048.

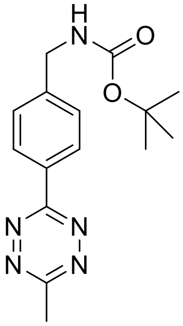

3h

***tert*-butyl (4-(6-methyl-1,2,4,5-tetrazin-3-yl)benzyl)carbamate (3h): 3h** was obtained according to the literature procedures and ^{1}H NMR was matched with the literature.[1]

^{1}H NMR (500 MHz, $CDCl_3$) δ 8.46 (d, 2H, J = 8.2 Hz), 7.44 (d, 2H, J = 8.2 Hz), 5.22 (bs, 1H), 4.38 (d, 2H, J = 5.7 Hz), 3.04 (s, 3H), 1.44 (s, 9H).

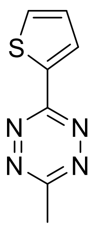

3i

3-methyl-6-(thiophen-2-yl)-1,2,4,5-tetrazine (3i): 3i was prepared according to the previously published procedure[1] from commercially available acetonitrile and thiophene-2-carbonitrile. The product was purified by flash column chromatography (ethyl acetate/hexane 1:10) to afford **3i**.

Physical Property: Orange-red crystal, m.p. = 132-133 ˚C.

Isolated Yield: 27%

TLC: R_f = 0.46 (silica gel, ethyl acetate/hexane 1:10).

^{1}H NMR (500 MHz, $CDCl_3$) δ 8.19 (dd, 1H, J = 3.7, 0.8 Hz), 7.62 (dd, 1H, J = 5.0, 0.8 Hz), 7.21-7.18 (m, 1H), 3.00 (s, 3H).

^{13}C NMR (125 MHz, $CDCl_3$) δ 166.7, 162.0, 135.8, 132.3, 131.0, 128.9, 21.2.

IR (neat): 1535, 1437, 1394, 1370, 1346, 1294, 1216, 1082, 1054, 991, 897, 854, 732, and 670 cm^{-1}.

HRMS (ESI) calculated for $C_7H_7N_4S$ $[M+H]^+$ 179.0386, found 179.0393.

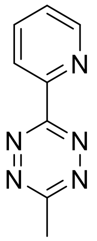

3j

3-methyl-6-(pyridin-2-yl)-1,2,4,5-tetrazine (3j): 3j was prepared according to the previously published procedure[1] from commercially available acetonitrile and 2-cyanopyridine. The product was purified by flash column chromatography (ethyl acetate/hexane 1:1) to afford **3j.**

Physical Property: Purple crystal, m.p. = 104-105 ˚C.

Isolated Yield: 11%

TLC: R_f = 0.06 (silica gel, ethyl acetate/hexane 1:4).

^{1}H NMR (500 MHz, $CDCl_3$) δ 8.70 (dd, 1H, J = 4.7, 0.7 Hz), 8.38 (d, 1H, J = 7.9 Hz), 7.76 (td, 1H, J = 7.8, 1.7 Hz), 7.34 (ddd, 1H, J = 7.6, 4.7, 1.0 Hz), 2.93 (s, 3H).

^{13}C NMR (125 MHz, $CDCl_3$) δ 167.7, 163.2, 150.5, 149.9, 137.1, 126.0, 123.5, 21.0.

IR (neat): 1641, 1584, 1567, 1400, 1364,1103, 1040, 992, 902, 778, 745, 736 cm^{-1}.

HRMS (ESI) calculated for $C_8H_8N_5$ $[M+H]^+$ 174.0775, found 174.0783.

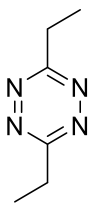

3k

3,6-diethyl-1,2,4,5-tetrazine (3k): 3k was prepared according to the previously published procedure[1] from commercially available propionitrile. The product was purified by flash column chromatography (ethyl acetate/hexane 1:20) to afford **3k**.

Physical Property: Red oil.

Isolated Yield: 38%

TLC: R_f = 0.60 (silica gel, ethyl acetate/hexane 1:4).

^{1}H NMR (500 MHz, $CDCl_3$) δ 3.22 (q, 4H, J = 7.6 Hz), 1.40 (t, 6H, J = 7.6 Hz).

^{13}C NMR (125 MHz, $CDCl_3$) δ 170.8, 28.2, 12.2.

IR (neat): 2983, 2942, 2884, 1688, 1464, 1399, 1266, 1056, 888, 737, 701, 652 cm^{-1}.

HRMS (ESI) calculated for $C_6H_{11}N_4$ $[M+H]^+$ 139.0973, no peak matched the calculated exact mass.

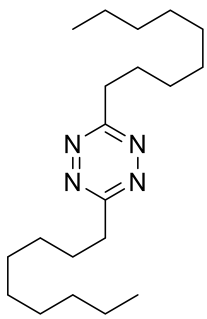

3l

3,6-dinonyl-1,2,4,5-tetrazine (3l): 3l was prepared according to the previously published procedure[1] from commercially available decanenitrile. was purified by flash column chromatography (ethyl acetate/hexane 1:20) to afford **3l**.

Physical Property: Red crystal, m.p. = 32-33 ˚C.

Isolated Yield: 40%

TLC: $\mathbf{R_f}$ = 0.75 (silica gel, ethyl acetate/hexane 1:4).

^{1}**H NMR** (500 MHz, $CDCl_3$) δ 3.27 (t, 4H, J = 7.7 Hz), 1.97-1.88 (m, 4H), 1.46-1.19 (m, 24H), 0.90-0.83 (m, 6H).

^{13}**C NMR** (125 MHz, $CDCl_3$) δ 170.2, 34.8, 31.9, 29.4, 29.3, 29.3, 29.2, 28.3, 22.7, 14.1.

IR (neat): 2928, 2856, 1458, 1395, 906, 731, 668 cm^{-1}.

HRMS (ESI) calculated for $C_{20}H_{39}N_4$ $[M+H]^+$ 335.3170, found 335.3180.

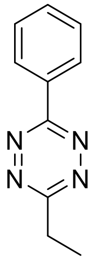

3m

3-ethyl-6-phenyl-1,2,4,5-tetrazine (3m): 3m was prepared according to the previously published procedure[1] from commercially available propionitrile and benzonitrile. The product was purified by flash column chromatography (ethyl acetate/hexane 1:12) to afford **3m**.

Physical Property: Red oil.

Isolated Yield: 69%

TLC: $\mathbf{R_f}$ = 0.57 (silica gel, ethyl acetate/hexane 1:4).

^{1}H NMR (500 MHz, $CDCl_3$) δ 8.57-8.53 (m, 2H), 7.58-7.52 (m, 3H), 3.36 (q, 2H, J = 7.6 Hz), 1.53 (t, 3H, J = 7.6 Hz).

^{13}C NMR (125 MHz, $CDCl_3$) δ 170.8, 164.2, 132.5, 131.9, 129.2, 127.9, 28.3, 12.3.

IR (neat): 1465, 1396, 1265, 1091, 898, 736, 692 cm^{-1}.

HRMS (ESI) calculated for $C_{10}H_{11}N_4$ $[M+H]^+$ 187.0980, no peak matched the calculated exact mass.

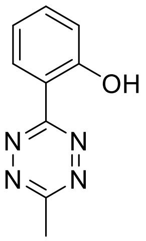

3n

2-(6-methyl-1,2,4,5-tetrazin-3-yl)phenol (3n): 3n was prepared according to the previously published procedure[1] from commercially available acetonitrile and 2-hydroxybenzonitrile. The product was purified by flash column chromatography (ethyl acetate/hexane 1:10) to afford **3n**.

Physical Property: Red crystal, m.p. = 63-64 ˚C.

Isolated Yield: 36%

TLC: $\mathbf{R_f}$ = 0.50 (silica gel, ethyl acetate/hexane 1:4).

^{1}**H NMR** (500 MHz, $CDCl_3$) δ 11.11 (bs, 1H), 8.64 (dd, 1H, J = 8.1, 1.7 Hz), 7.52 (ddd, 1H, J = 8.3, 7.2, 1.7 Hz), 7.14-7.05 (m, 2H), 3.12 (s, 3H).

^{13}**C NMR** (125 MHz, $CDCl_3$) δ 166.6, 164.9, 160.0, 135.1, 128.6, 120.3, 118.7, 114.1, 21.3.

IR (neat): 3053, 1618, 1585, 1484, 1402, 1265, 1234, 1085, 909, 759, 735 cm^{-1}.

HRMS (ESI) calculated for $C_9H_9N_4O$ $[M+H]^+$ 189.0772, no peak matched the calculated exact mass.

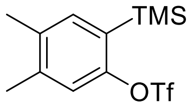

4b

4,5-dimethyl-2-(trimethylsilyl)phenyl trifluoromethanesulfonate (4b): 4b was obtained according to the literature procedures and ^{1}H NMR was matched with the literature.[7]

Isolated Yield: 82%.

^{1}**H NMR** (500 MHz, $CDCl_3$) δ 7.25 (s, 1H), 7.10 (s, 1H), 2.29 (s, 3H), 2.27 (s, 3H), 0.36 (s, 9H).

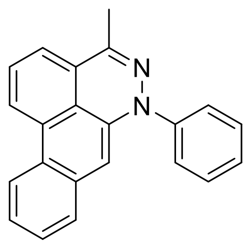

5a

4-methyl-6-phenyl-6*H*-dibenzo[*de,g*]cinnoline (5a): 5a was prepared according to the general procedure using **3a** and **4a**. The product was purified by flash column chromatography (ethyl acetate/hexane 1:20) to afford **5a**.

Physical Property: Yellow solid, m.p. = 145-146 ˚C.

Isolated Yield: 51%.

TLC: R_f = 0.60 (silica gel, ethyl acetate/hexane 1:4).

^{1}H NMR (500 MHz, CD_2Cl_2) δ 8.39 (d, 1H, J = 8.3 Hz), 8.34 (d, 1H, J = 8.2 Hz), 7.64-7.56 (m, 5H), 7.46-7.37 (m, 3H), 7.30 (ddd, 1H, J = 8.3, 6.2, 2.1 Hz), 7.26 (dd, 1H, J = 7.4, 0.9 Hz), 6.43 (s, 1H), 2.29 (s, 3H).

^{13}C NMR (125 MHz, CD_2Cl_2) δ 144.4, 142.6, 137.7, 134.3, 131.8, 130.3, 128.7, 127.9, 127.8, 127.1, 127.1, 126.3, 126.2, 124.3, 123.1, 123.0, 122.8, 118.2, 97.7, 19.1.

IR (neat): 3057, 2920, 2850, 1618, 1588, 1496, 1441, 1400, 1340, 1305, 1272, 1233, 1207, 1142, 1024, 807, 756, 744, 735, 699, and 644 cm^{-1}.

HRMS (ESI) calculated for $C_{22}H_{17}N_2$ $[M+H]^+$ 309.1387, found 309.1406.

Quantum Yield (Φ):

(a) 1.5 % of the neutral form.

(b) 27.7 % in the presence of 1073 equivalents of trifluoroacetic acid**.**

Extinction Coefficient (ε):

(a) 7362 ± 164 (417 nm), 16847 ± 348 (349 nm), 18285 ± 419 (269 nm), 34084 ± 214 (231 nm) for the neutral form.

(b) 7378 ± 181 (411 nm), 46894 ± 370 (230 nm) in the presence of of 1073 equivalents of trifluoroacetic acid**.**

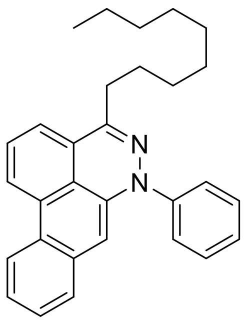

5b

4-nonyl-6-phenyl-6*H*-dibenzo[*de,g*]cinnoline (5b): 3b was prepared according to the general procedure using **3b** and **4a**. The product was purified by flash column chromatography (ethyl acetate/hexane 1:25) to afford **5b**.

Physical Property: Yellow solid, m.p. = 99-100 ˚C.

Isolated Yield: 41%.

TLC: $\mathbf{R_f}$ = 0.74 (silica gel, diethylether/hexane 1:4).

^{1}**H NMR** (500 MHz, CD_2Cl_2) δ 8.38 (d, 1H, J = 8.0 Hz), 8.34 (d, 1H, J = 8.1 Hz), 7.63-7.54 (m, 5H), 7.44-7.36 (m, 3H), 7.33-7.28 (m, 2H), 6.45 (s, 1H), 2.72-2.60 (m, 2H), 1.79-1.69 (m, 2H), 1.50-1.42 (m, 2H), 1.41-1.20 (m, 10H), 0.91-0.85 (m, 3H).

^{13}**C NMR** (125 MHz, CD_2Cl_2) δ 145.8, 144.5, 137.7, 134.3, 132.0, 130.3, 128.7, 127.8, 127.1, 127.0, 126.6, 125.7, 124.4, 123.1, 122.78, 122.76, 118.0, 97.6, 32.7, 32.3, 30.04, 30.01, 29.9, 29.8, 27.1, 23.1, 14.3.

IR (neat): 3050, 2925, 2852, 1616, 1588, 1496, 1461, 1442, 1399, 1338, 1301, 1264, 1232, 1206, 1142, 815, 738, 704, 668, and 649 cm^{-1}.

HRMS (ESI) calculated for $C_{30}H_{33}N_2$ $[M+H]^+$ 421.2639, found 421.2630.

Quantum Yield (Φ):

(a) 1.2% of the neutral form.

(b) 6.6% in the presence of 1048 equivalents of trifluoroacetic acid**.**

Extinction Coefficient (ε):

(a) 9205 ± 358 (419 nm), 15954 ± 109 (350 nm), 17336 ± 601 (269 nm), 34072 ± 342 (229 nm) for the neutral form.

(b) 7103 ± 16 (419 nm), 42445 ± 459 (229 nm) in the presence of 1048 equivalents of trifluoroacetic acid**.**

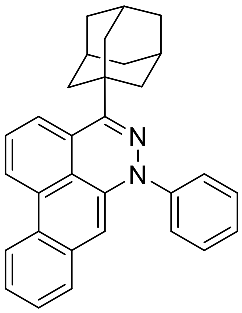

5c

4-((3*r*,5*r*,7*r*)-adamantan-1-yl)-6-phenyl-6*H*-dibenzo[*de,g*]cinnoline (5c): 5c was prepared according to the general procedure using **1c** and **4a**. The product was purified by flash column chromatography (ethyl acetate/hexane 1:30) to afford **5c**.

Physical Property: Yellow oil.

Isolated Yield 10%.

TLC: R_f = 0.79 (silica gel, ethyl acetate/hexane 1:4).

^{1}**H NMR** (500 MHz, $CDCl_3$) δ 8.37 (m, 2H), 7.90 (d, 1H, J = 7.5 Hz), 7.66-7.52 (m, 5H), 7.44-7.36 (m, 3H), 7.31 (ddd, 1H, J = 8.3, 6.2, 1.7 Hz), 6.56 (s, 1H), 2.25-2.19 (m, 6H), 2.14 (bs, 3H), 1.90-1.80 (m, 6H).

^{13}**C NMR** (125 MHz, $CDCl_3$) δ 149.4, 144.3, 136.5, 133.5, 132.0, 129.6, 127.5, 127.30, 127.28, 126.9, 126.4, 125.7, 124.13, 123.9, 122.7, 122.3, 121.8, 120.1, 96.9, 40.5, 40.0, 37.0, 29.0.

IR (neat): 2903, 2848, 1612, 1584, 1494, 1440, 1392, 1339, 1288, 1233, 1144, 760, 736 cm^{-1}.

HRMS (ESI) calculated for $C_{31}H_{28}N_2$ $[M+H]^+$ 429.2326, found 429.2298.

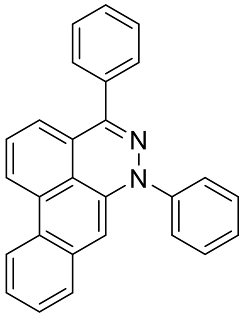

5d

4,6-diphenyl-6*H*-dibenzo[*de,g*]cinnoline (5d): 5d was prepared according to the general procedure using **3d** and **4a**. The product was purified by flash column chromatography (ethyl acetate/hexane 1:20) to afford **5d**.

Physical Property: Yellow solid, m.p. = 135-136 ˚C.

Isolated Yield 26%.

TLC: R_f = 0.53 (silica gel, diethylether/hexane 1:4).

^{1}H NMR (500 MHz, CD_2Cl_2) δ 8.37 (d, 1H, J = 8.0 Hz), 8.34 (d, 1H, J = 8.1 Hz), 7.65-7.60 (m, 4H), 7.59-7.54 (m, 2H), 7.53-7.37 (m, 7H), 7.33 (ddd, 1H, J = 8.2, 6.3, 2.1 Hz), 7.20 (dd, 1H, J = 7.5, 0.9 Hz), 6.45 (s, 1H).

^{13}C NMR (125 MHz, CD_2Cl_2) δ 146.0, 143.7, 137.2, 136.0, 133.8, 131.7, 129.9, 128.6, 128.53, 128.51, 128.1, 127.6, 127.5, 126.8, 126.4, 125.4, 124.2, 123.0, 122.6, 122.4, 119.4, 97.7.

IR (neat): 3052, 2918, 2848, 1614, 1586, 1492, 1458, 1441, 1398, 1320, 1237, 1145, 1129, 984, 816, 761, 735, and 698 cm^{-1}.

HRMS (ESI) calculated for $C_{27}H_{19}N_2$ $[M+H]^+$ 371.1543, found 371.1547.

Quantum Yield (Φ):

(a) 0.7% of the neutral form

(b) 8.1% in the presence of 1034 equivalents of trifluoroacetic acid**.**

Extinction Coefficient (ε):

(a) 4478 ± 140 (419 nm), 13669 ± 371 (357 nm), 18217 ± 536 (261 nm), 25145 ± 719 (242 nm) for the neutral form.

(b) 7346 ± 19 (417 nm), 25976 ± 371 (404 nm) in the presence of 1034 equivalents of trifluoroacetic acid**.**

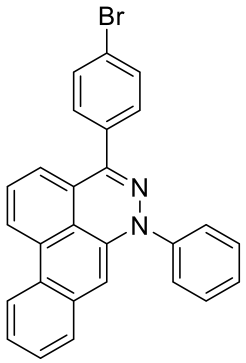

5e

4-(4-bromophenyl)-6-phenyl-6*H*-dibenzo[*de,g*]cinnoline (5e): 5e was prepared according to the general procedure using **3e** and **4a**. The product was purified by flash column chromatography (ethyl acetate/hexane 1:20) to afford **5e**.

Physical Property: Yellow solid, m.p. = 188-189 ˚C.

Isolated Yield 46%.

TLC: $\mathbf{R_f}$ = 0.59 (silica gel, diethylether/hexane 1:4).

^{1}H NMR (500 MHz, CD_2Cl_2) δ 8.37 (d, 1H, J = 8.3 Hz), 8.33 (d, 1H, J = 8.2 Hz), 7.65-7.38 (m, 12H), 7.33 (ddd, 1H, J = 8.1, 5.9, 2.4 Hz), 7.21 (dd, 1H, J = 7.4, 0.5 Hz), 6.45 (s, 1H).

^{13}C NMR (125 MHz, CD_2Cl_2) δ 144.8, 143.6, 137.1, 135.0, 133.7, 131.8, 131.7, 130.2, 129.9, 128.1, 127.7, 127.5, 126.92, 126.88, 126.5, 125.1, 124.2, 123.2, 122.7, 122.4, 122.4, 119.1, 98.0.

IR (neat): 3064, 2916, 2848, 2176, 1614, 1588, 1493, 1456, 1441, 1315, 1237, 1144, 1128, 1080, 1011, 984, 832, 757, 737, and 625 cm^{-1}.

HRMS (ESI) calculated for $C_{27}H_{18}BrN_2$ $[M+H]^+$ 449.0648, found 449.0643.

Quantum Yield (Φ):

(a) 0.5% of the neutral form.

(b) 6.9% in the presence of 1083 equivalents of trifluoroacetic acid**.**

Extinction Coefficient (ε):

(a) 5949 ± 130 (418 nm), 19021 ± 243 (358 nm), 34833 ± 827 (243 nm) for the neutral form.

(b) 7960 ± 63 (418 nm), 11769 ± 581 (356 nm), 40935 ± 701 (242 nm) in the presence of 1083 equivalents of trifluoroacetic acid**.**

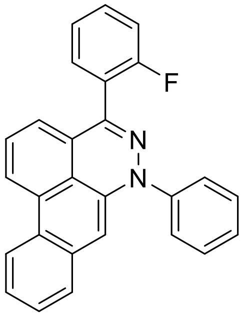

5f

4-(2-fluorophenyl)-6-phenyl-6*H*-dibenzo[*de,g*]cinnoline (5f): 5f was prepared according to the general procedure using **3f** and **4a**. The product was purified by flash column chromatography (ethyl acetate/hexane 1:20) to afford **5f**.

Physical Property: Yellow solid, m.p. = 80-81 ˚C.

Isolated Yield 19%.

TLC: R_f = 0.63 (silica gel, ethyl acetate/hexane 1:4).

^{1}H NMR (500 MHz, $CDCl_3$) δ 8.40-8.33 (m, 2H), 7.66-7.39 (m, 10H), 7.38-7.33 (m, 1H), 7.31 (td, 1H, J = 7.5, 1.2 Hz), 7.26-7.21 (m, 1H), 6.92 (ddd, 1H, J = 7.4, 2.9, 0.9 Hz).

^{13}C NMR (125 MHz, $CDCl_3$) δ 161.7, 159.7, 143.5, 141.7, 137.1, 133.7, 131.6, 131.43, 131.40, 130.73, 130.66, 130.0, 128.3, 127.8, 127.5, 126.9, 126.5, 125.5, 124.70, 124.67, 124.3, 123.7, 123.6, 123.2, 122.7, 122.4, 119.25, 119.23, 116.0, 115.8, 98.1.

^{19}F NMR (338 MHz, CD2Cl2) δ -113.3 to -113.4 (m, 1F).

IR (neat): 1738, 1373, 1238, 1045, 939, 918 cm^{-1}.

HRMS (ESI) calculated for $C_{27}H_{18}FN_2$ $[M+H]^+$ 389.1449, found 389.1454.

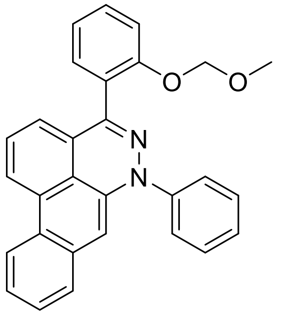

5g

4-(2-(methoxymethoxy)phenyl)-6-phenyl-6*H*-dibenzo[*de,g*]cinnoline (5g): 5g was prepared according to the general procedure using **3g** and **4a**. The product was purified by flash column chromatography (ethyl acetate/hexane 1:15) to afford **5g**.

Physical Property: Yellow oil.

Isolated Yield 25%.

TLC: $\mathbf{R_f}$ = 0.45 (silica gel, ethyl acetate/hexane 1:4).

^{1}**H NMR** (500 MHz, $CDCl_3$) δ 8.35 (dd, 2H, J = 8.6, 2.8 Hz), 7.66-7.61 (m, 2H), 7.60-7.54 (m, 2H), 7.51-7.38 (m, 6H), 7.33 (ddd, 1H, J = 8.3, 6.6, 1.7 Hz), 7.25 (d, 1H, J = 8.0 Hz), 7.14 (td, 1H, J = 7.6, 1.0 Hz), 6.84 (dd, 1H, J = 7.3, 0.7 Hz), 6.50 (s, 1H), 5.15 (m, 2H), 3.36 (s, 3H).

^{13}**C NMR** (125 MHz, $CDCl_3$) δ 155.6, 144.5, 143.7, 137.2, 133.8, 131.4, 130.9, 130.1, 129.8, 128.1, 127.5, 127.4, 126.8, 126.4, 126.3, 125.95, 125.90, 124.2, 123.0, 122.3, 122.2, 119.8, 114.8, 97.6, 94.7, 56.1.

IR (neat): 3053, 1587, 1493, 1453, 1440, 1398, 1319, 1264, 1234, 1153, 996, 735, 703 cm^{-1}.

HRMS (ESI) calculated for $C_{29}H_{23}N_2O_2$ $[M+H]^+$ 431.1755, found 431.1760.

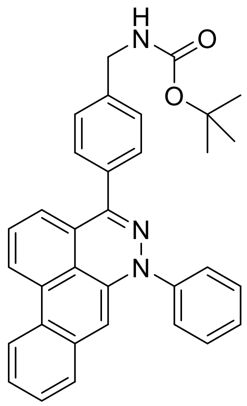

5h

***tert*-butyl (4-(6-phenyl-6*H*-dibenzo[*de,g*]cinnolin-4-yl)benzyl)carbamate (5h): 5h** was prepared according to the general procedure using **3h** and **4a**. The product was purified by flash column chromatography (ethyl acetate/hexane 1:4) to afford **5h**.

Physical Property: Yellow solid, m.p. = 177-178 ˚C.

Isolated Yield: 31%.

TLC: R_f = 0.21 (silica gel, ethyl acetate/hexane 1:4).

^{1}H NMR (500 MHz, CD_2Cl_2) δ 8.40-8.25 (m, 2H), 7.67-7.56 (m, 5H), 7.55-7.49 (m, 1H), 7.48-7.38 (m, 5H), 7.34 (ddd, 1H, J = 8.3, 6.1, 2.2 Hz), 7.27 (d, 1H, J = 7.3 Hz), 6.47 (s, 1H), 5.03 (bs, 1H), 4.37 (d, 1H, J = 5.8 Hz), 1.48 (s, 9H).

^{13}C NMR (125 MHz, CD_2Cl_2) δ 155.8, 145.7, 143.7, 140.1, 137.2, 134.9, 133.8, 131.8, 129.9, 128.7, 128.1, 127.7, 127.5, 127.4, 126.9, 126.8, 126.5, 125.4, 124.2, 123.1, 122.6, 122.4, 119.5, 97.8, 79.2, 44.3, 28.1.

IR (neat): 3345, 2976, 1707, 1612, 1586, 1492, 1440, 1397, 1365, 1318, 1288, 1271, 1237, 1167, 1145, 1129,760, 737, 695 cm^{-1}.

HRMS (ESI) calculated for $C_{33}H_{30}N_3O_2$ $[M+H]^+$ 500.2333, found 500.2332.

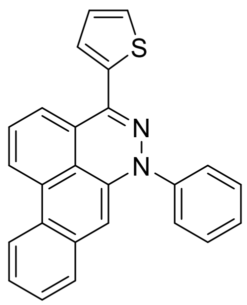

5i

6-phenyl-4-(thiophen-2-yl)-6*H*-dibenzo[*de,g*]cinnoline (5i): 5i was prepared according to the general procedure using **3i** and **4a**. The product was purified by flash column chromatography (ethyl acetate/hexane 1:20) to afford **5i**.

Physical Property: Red solid, m.p. = 139-140 ˚C.

Isolated Yield 52%.

TLC: R_f = 0.56 (silica gel, diethylether/hexane 1:4).

^{1}H NMR (500 MHz, CD_2Cl_2) δ 8.40 (d, 1H, J = 8.4 Hz), 8.35 (d, 1H, J = 8.1 Hz), 7.66 (dd, 1H, J = 7.5, 1.0 Hz), 7.64-7.53 (m, 6H), 7.48-7.38 (m, 4H), 7.34 (ddd, 1H, J = 8.2, 6.0, 2.2 Hz), 7.16 (dd, 1H, J = 5.1, 3.5 Hz), 6.47 (s, 1H).

^{13}C NMR (125 MHz, CD_2Cl_2) δ 143.9, 140.4, 138.8, 137.2, 134.1, 132.3, 130.4, 128.7, 128.3, 128.0, 127.5, 127.4, 127.3, 127.1, 126.9, 126.1, 125.4, 124.8, 123.8, 123.3, 122.9, 119.5, 98.8.

IR (neat): 3057, 2922, 2850, 1614, 1586, 1492, 1458, 1443, 1394, 1338, 1293, 1238, 1146, 1128, 947, 798, 752, 700, 694, and 674 cm^{-1}.

HRMS (ESI) calculated for $C_{25}H_{17}N_2S$ $[M+H]^+$ 377.1107, found 377.1099.

Quantum Yield (Φ):

(a) 0.6% of the neutral form.

(b) 1.5% in the presence of 1001 equivalents of trifluoroacetic acid**.**

Extinction Coefficient (ε):

(a) 4610 ± 20 (418 nm), 14238 ± 20 (362 nm), 27222 ± 192 (244 nm) for the neutral form.

(b) 6395 ± 318 (419 nm), 9607 ± 879 (360 nm), 31340 ± 27 (242 nm), 38234 ± 959 (228 nm) in the presence of 1001 equivalents of trifluoroacetic acid**.**

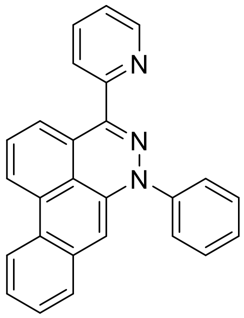

5j

6-phenyl-4-(pyridin-2-yl)-6*H*-dibenzo[*de,g*]cinnoline (5j): 5j was prepared according to the general procedure using **3j** and **4a**. The product was purified by flash column chromatography (ethyl acetate/hexane 1:4) to afford **5j**.

Physical Property: Orange solid, m.p. = 139-140 ˚C.

Isolated Yield 15%.

TLC: R_f = 0.12 (silica gel, diethylether/hexane 1:4).

^{1}H NMR (500 MHz, CD_2Cl_2) δ 8.70 (d, 1H, J = 4.5 Hz), 8.40 (d, 1H, J = 8.3 Hz), 8.37 (d, 1H, J = 8.2 Hz), 8.04 (d, 1H, J = 7.6 Hz), 7.81 (td, 1H, J = 7.7, 1.8 Hz), 7.76 (d, 1H, J = 7.9 Hz), 7.68-7.55 (m, 5H), 7.51-7.33 (m, 5H), 6.45 (s, 1H).

^{13}C NMR (125 MHz, CD_2Cl_2) δ 156.0, 148.8, 144.1, 143.8, 137.6, 137.3, 134.0, 132.1, 130.5, 128.6, 128.3, 127.9, 127.7, 127.3, 127.1, 125.01, 124.99, 123.74, 123.73, 123.6, 123.0, 122.8, 121.0, 98.7.

IR (neat): 3052, 2922, 1614, 1586, 1494, 1472, 1458, 1443, 1429, 1398, 1338, 1322, 1264, 1150, 1055, 997, 819, 736, 708, 698, and 645 cm^{-1}.

HRMS (ESI) calculated for $C_{26}H_{18}N_3$ $[M+H]^+$ 372.1496, found 372.1498.

Quantum Yield (Φ):

(a) 0.6% of the neutral form

(b) 0.0% in the presence of 1048 equivalents of trifluoroacetic acid**.**

Extinction Coefficient (ε):

(a) 1641 ± 46 (413 nm), 5306 ± 80 (361 nm), 17336 ± 601 (271 nm) for the neutral form.

(b) 1749 ± 100 (560 nm), 3857 ± 118 (365 nm), 26933 ± 133 (228 nm) in the presence of 1048 equivalents of trifluoroacetic acid**.**

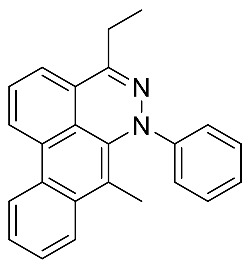

5k

4-ethyl-7-methyl-6-phenyl-6*H*-dibenzo[*de,g*]cinnoline (5k): 5k was prepared according to the general procedure using **3k** and **4a**. The product was purified by flash column chromatography (ethyl acetate/hexane 1:30) to afford **5k**.

Physical Property: Yellow solid, m.p. = 60-61 ˚C.

Isolated Yield: 44%.

TLC: $\mathbf{R_f}$ = 0.80 (silica gel, ethyl acetate/hexane 1:4).

^{1}**H NMR** (500 MHz, CD_2Cl_2) δ 8.53-8.48 (m, 2H), 7.87 (dd, 1H, J = 8.4, 0.6 Hz), 7.64-7.56 (m, 2H) 7.48 (ddd, 1H, J = 8.2, 7.0, 1.2 Hz), 7.40 (dd, 1H, J = 7.4, 0.8 Hz), 7.40-7.33 (m, 4H), 7.10 (tt, 1H, 6.8, 1.7 Hz), 2.82 (q, 2H, 7.4 Hz), 2.01 (s, 3H), 1.37 (t, 3H, 7.5 Hz)

^{13}**C NMR** (125 MHz, CD_2Cl_2) δ 149.7, 148.2, 134.3, 132.1, 129.6, 128.8, 127.3, 127.2, 126.9, 125.6, 123.5, 123.4, 122.9, 122.6, 121.2, 118.4, 109.2, 25.4, 16.9, 10.8.

IR (neat): 2916, 2849, 2360, 2342, 1587, 1485, 1446, 1388, 1372, 1335, 1283, 1265, 1231, 1212, 1138, 1032, 1012, 752, 737, 691, 668 cm^{-1}.

HRMS (ESI) calculated for $C_{24}H_{20}N_2$ $[M]^+$ 336.1621, found 336.1630.

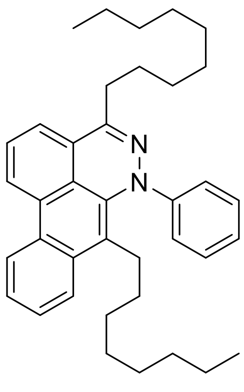

5l

4-nonyl-7-octyl-6-phenyl-6*H*-dibenzo[*de,g*]cinnoline (5l): 5l was prepared according to the general procedure using **3l** and **4a**. The product was purified by flash column chromatography (ethyl acetate/hexane 1:30) to afford **5l**.

Physical Property: Yellow oil.

Isolated Yield: 24%.

TLC: $\mathbf{R_f}$ = 0.80 (silica gel, ethyl acetate/hexane 1:4).

^{1}**H NMR** (500 MHz, CD_2Cl_2) δ 8.51 (d, 1H, J = 8.6 Hz), 8.48 (d, 1H, J = 8.2 Hz), 7.89 (d, 1H, J = 8.4 Hz), 7.63-7.58 (m, 1H), 7.55 (ddd, 1H, J = 8.5, 6.9, 1.3 Hz), 7.46 (ddd, 1H, J = 8.1, 6.9, 1.1 Hz), 7.43-7.38 (m, 3H), 7.36-7.30 (m, 2H), 7.10 (tt, 1H, J = 7.3, 1.1 Hz), 2.76 (t, 2H, J = 7.7 Hz), 2.59 (t, 2H, J = 7.6 Hz), 1.83-1.75 (m, 2H), 1.52-1.44 (m, 2H), 1.42-1.22 (m, 14H), 1.22-1.04 (m, 8H), 0.94-0.82 (m, 6H).

^{13}**C NMR** (125 MHz, CD_2Cl_2) δ 149.1, 148.9, 133.5, 132.1, 129.6, 129.0, 127.4, 127.0, 126.8, 126.2, 124.1, 123.89, 123.86, 123.4, 122.9, 122.8, 121.3, 118.5, 115.5, 32.2, 31.8, 31.8, 29.5, 29.5, 29.4, 29.32, 29.28, 29.19, 29.1, 28.2, 26.7, 22.67, 22.60, 13.84, 13.82.

IR (neat): 2954, 2924, 2853, 2361, 2340, 1586, 1490, 1438, 1386, 1332, 1281, 1264, 1228, 1211, 1136, 818, 755, 694, 612 cm^{-1}.

HRMS (ESI) calculated for $C_{38}H_{48}N_2$ $[M]^+$ 532.3812, found 532.3821.

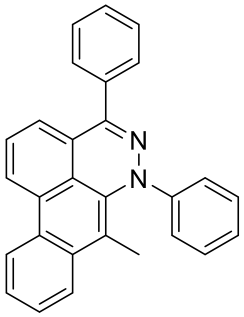

5m

7-methyl-4,6-diphenyl-6*H*-dibenzo[*de,g*]cinnoline (5m): 5m was prepared according to the general procedure using **3m** and **4a**. The product was purified by flash column chromatography (ethyl acetate/hexane 1:20) to afford **5m**.

Physical Property: Yellow oil.

Isolated Yield: 11%.

TLC: R_f = 0.61 (silica gel, ethyl acetate/hexane 1:4).

^{1}H NMR (500 MHz, CD_2Cl_2) δ 8.53-8.48 (m, 2H), 7.88 (dd, 1H, J = 8.3, 0.9 Hz), 7.68-7.63 (m, 2H), 7.59 (ddd, 1H, J = 8.3, 6.9, 1.3 Hz), 7.55-7.45 (m, 5H), 7.43-7.33 (m, 4H), 7.30 (dd, 1H, J = 7.5, 0.9 Hz), 7.13 (tt, 1H, 7.1, 1.5 Hz), 2.02 (s, 3H).

^{13}C NMR (125 MHz, CD_2Cl_2) δ 149.1, 148.1, 135.9, 134.5, 132.1, 129.8, 128.9, 128.82, 128.80, 128.5, 128.1, 127.5, 126.7, 125.7, 123.88, 123.86, 123.81, 123.6, 123.1, 122.7, 121.3, 120.6, 109.4, 16.8

IR (neat): 2916, 2849, 2348, 1594, 1489, 1460, 1445, 1372, 1138, 1031, 755, 696, 655, 644 cm^{-1}.

HRMS (ESI) calculated for $C_{28}H_{20}N_2$ $[M]^+$ 384.1621, found 384.1612.

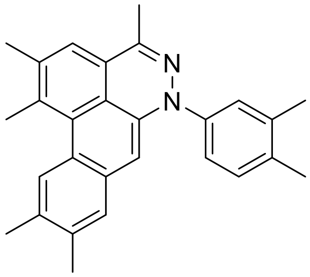

5n

6-(3,4-dimethylphenyl)-1,2,4,9,10-pentamethyl-6*H*-dibenzo[*de,g*]cinnoline (5n): 5n was prepared according to the general procedure using **1a** and **4b**. The product was purified by flash column chromatography (ethyl acetate/hexane 1:20) to afford **5n**.

Physical Property: Yellow oil.

Isolated Yield: 24%.

TLC: R_f = 0.54 (silica gel, ethyl acetate/hexane 1:4).

^{1}H NMR (500 MHz, CD_2Cl_2) δ 8.20 (s, 1H), 7.33-7.25 (m, 2H), 7.25-7.20 (m, 1H), 7.16 (s, 1H), 7.12 (s, 1H), 6.21 (s, 1H), 2.85 (s, 3H), 2.54 (s, 3H), 2.41 (s, 3H), 2.36 (s, 3H), 2.34 (s, 3H), 2.33 (s, 3H), 2.27 (s, 3H).

^{13}C NMR (125 MHz, CD_2Cl_2) δ 142.0, 141.3, 138.5, 137.9, 137.0, 136.0, 135.8, 134.2, 133.7, 131.3, 130.7, 129.7, 128.2, 127.9, 126.3, 125.0, 124.0, 123.3, 123.0, 119.5, 96.7, 21.7, 21.6, 20.1, 19.6, 19.5, 19.22, 18.7.

IR (neat): 2916, 2857, 1606, 1502, 1449, 1372, 1300, 1235, 1194, 1170, 1086, 1014, 871, 803, 736 cm^{-1}.

HRMS (ESI) calculated for $C_{28}H_{28}N_2$ $[M]^+$ 392.2247, found 392.2252.

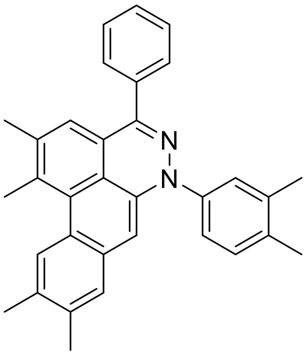

5o

6-(3,4-dimethylphenyl)-1,2,9,10-tetramethyl-4-phenyl-6*H*-dibenzo[*de,g*]cinnoline (5o): 5o was prepared according to the general procedure using **3d** and **4b**. The product was purified by flash column chromatography (ethyl acetate/hexane 1:20) to afford **5o**.

Physical Property: Yellow oil.

Isolated Yield: 31%.

TLC: R_f = 0.59 (silica gel, ethyl acetate/hexane 1:4).

^{1}H NMR (500 MHz, CD_2Cl_2) δ 8.18 (s, 1H), 7.63-7.60 (m, 2H), 7.50-7.40 (m, 3H), 7.35-7.25 (m, 3H), 7.17 (s, 1H), 7.09 (s, 1H), 6.25 (s, 1H), 2.83 (s, 3H), 2.423 (s, 3H), 2.416 (s, 3H), 2.36 (s, 3H), 2.34 (s, 3H), 2.35-2.32 (m, 6H).

^{13}C NMR (125 MHz, CD_2Cl_2) δ 145.3, 141.8, 138.6, 137.9, 136.9, 136.4, 136.2, 136.0, 134.1, 133.9, 131.7, 130.7, 130.7, 130.1, 128.6, 128.5, 128.4, 128.2, 127.6, 126.4, 126.2, 123.8, 123.6, 122.4, 121.1, 29.7, 21.5, 20.1, 19.6, 19.4, 19.2.

IR (neat): 2921, 1603, 1500, 1418, 1300, 1264, 1227, 1141, 1068, 1005, 878, 831, 738, 687 cm^{-1}.

HRMS (ESI) calculated for $C_{33}H_{30}N_2$ $[M]^+$ 454.2404, found 454.2403.

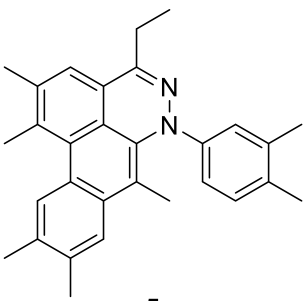

5p

6-(3,4-dimethylphenyl)-4-ethyl-1,2,7,9,10-pentamethyl-6*H*-dibenzo[*de,g*]cinnoline (5p): 5p was prepared according to the general procedure using **3k** and **4b**. The product was purified by flash column chromatography (ethyl acetate/hexane 1:20) to afford **5p**.

Physical Property: Yellow oil.

Isolated Yield: 30%.

TLC: R_f = 0.69 (silica gel, ethyl acetate/hexane 1:4).

^{1}H NMR (500 MHz, CD_2Cl_2) δ 8.22 (s, 1H), 7.55 (s, 1H), 7.27 (s, 1H), 7.11 (d, 1H, J = 2.3 Hz), 7.04 (d, 1H, J = 8.2 Hz), 6.92 (dd, 1H, J = 8.2, 2.3 Hz), 2.85 (s, 3H), 2.79 (q, 2H, J = 7.4 Hz), 2.55 (s, 3H), 2.48 (s, 3H), 2.46 (s, 3H), 2.242 (s, 3H), 2.237 (s, 3H), 1.91 (s, 3H), 1.33 (3H, t, 7.4 Hz).

^{13}C NMR (125 MHz, CD_2Cl_2) δ 148.6, 146.6, 137.2, 136.7, 135.8, 134.9, 133.9, 131.8, 131.6, 130.4, 129.7, 129.5, 128.9, 126.8, 124.7, 123.0, 122.7, 120.7, 120.5, 118.7, 108.2, 25.5, 21.7, 21.6, 20.1, 20.0, 19.9, 19.0, 16.8, 11.3.

IR (neat): 2968, 2917, 1607, 1594, 1499, 1488, 1453, 1357, 1264, 1195, 1166, 1002, 865, 820, 737 cm^{-1}.

HRMS (ESI) calculated for $C_{30}H_{32}N_2$ $[M]^+$ 420.2560, found 420.2550.

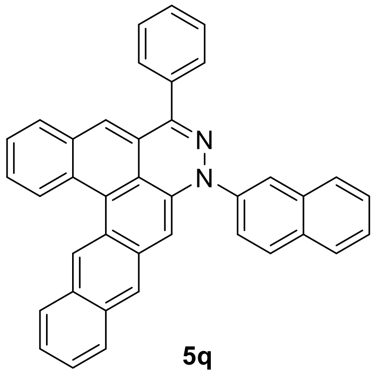

5q

8-(naphthalen-2-yl)-6-phenyl-8*H*-dinaphtho[3,2,1-*de*:2',3'-*g*]cinnoline (5q): A vial was charged with 14.8 mg (0.086 mmol) of **3d** and 300 mg (0.86 mmol) of 3-(trimethylsilyl)-2-naphthyl trifluoromethanesulfonate in 0.09 mL of dichloromethane at 24˚C, and 1 M TBAF in THF (0.95 mL, 0.95 mmol) was slowly added to the solution over the course of 1 min. After the addition, the solution was concentrated in vacuo, and fiterated on the silica gel, and purified by preparative TLC using ethyl acetate/hexane (1:10) as the eluent to give 0.61 mg of **5q**.

Physical Property: Red solid.

Isolated Yield: 1 %.

TLC: $\mathbf{R_f}$ = 0.50 (silica gel, ethyl acetate/hexane 1:4).

^{1}**H NMR** (500 MHz, CD_2Cl_2) δ 9.43 (s, 1H), 9.18 (d, 1H, J = 8.6 Hz), 8.21 (s, 1H), 8.15-7.94 (m, 6H), 7.90 (d, 1H, J = 8.0 Hz), 7.88-7.44 (m, 13H), 7.01 (s, 1H).

^{13}**C NMR** (125 MHz, CD_2Cl_2) δ 146.3, 141.9, 136.78, 136.73, 134.7, 134.6, 133.5, 133.1, 132.9, 131.3, 130.9, 130.2, 129.7, 129.4, 129.3, 129.2, 129.1, 128.7, 128.5, 128.31, 128.26, 128.0, 127.8, 127.4, 127.3, 127.1, 126.9, 126.8, 125.8, 125.5, 125.4, 124.8, 124.2, 124.0, 122.8, 122.0, 101.2.

HRMS (ESI) calculated for $C_{38}H_{48}N_2$ $[M]^+$ 520.1934, found 520.1930.

Quantum Yield (Φ):

(a) 6.7% of the neutral form.

(b) 2.0% in the presence of 1001 equivalents of trifluoroacetic acid**.**

Extinction Coefficient (ε):

(a) 2061 ± 339 (532 nm), 1850 ± 355 (501 nm), 9469 ± 57 (362 nm), 8663 ± 100 (340 nm), 23217 ± 698 (266 nm), 23141 ± 720 (257 nm) for the neutral form.

(b) 2290 ± 519 (524 nm), 15500 ± 793 (279 nm) in the presence of 1001 equivalents of trifluoroacetic acid**.**

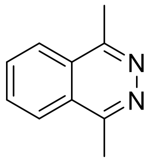

6

1,4-dimethylphthalazine (6): A vial was charged with 400 mg (4 mmol) of **1a** and 0.48 mL (2 mmol) of **2** in 2.0 mL of dichloromethane at 24 ˚C, and 1 M TBAF in THF (2.2 mmol, 1.1 mL) was slowly added to the solution over the course of 1 min. After the addition, the solution was concentrated in vacuo, and filtered on the silica gel, and purified by flash column chromatography (ethyl acetate = 100%) to afford 23 mg of **4**. The data for this compound was previously reported in the literature.[8]

Physical Property: White solid.

Isolated Yield: 7 %.

TLC: $\mathbf{R_f}$ = 0.10 (silica gel, 100% ethyl acetate).

^{1}H NMR (500 MHz, $CDCl_3$)

δ 8.03 (dd, 2H, J = 6.3, 3.3 Hz), 7.86 (dd, 2H, J = 6.3, 3.3 Hz), 2.93 (s, 6H).

^{13}C NMR (125 MHz, $CDCl_3$)

δ 156.26, 131.80, 125.85, 124.79, 19.76.

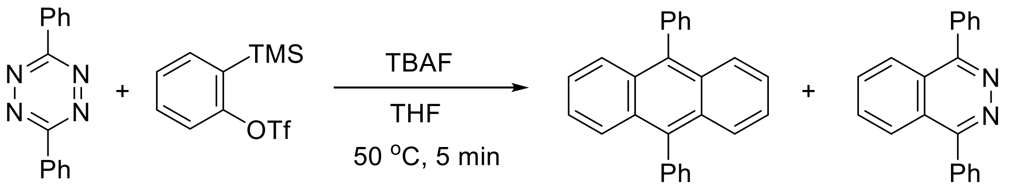

1 **4a** **7** **2**

9,10-diphenylanthracene (7) and 1,4-diphenylphthalazine (2): A vial was charged with 23.4 mg (0.1 mmol) of **1** and 298.4 mg (1 mmol) of **4a** in 0.1 mL of THF at 24 ˚C, and 1 M TBAF in THF (1.1 mmol, 1.1 mL) was added to the solution. After the addition, the solution was heated to 50 °C for 2 hours and athen was concentrated in vacuo, and filtered on the silica gel, and purified by flash column chromatography (ethyl acetate/hexane 1:4) to afford 10 mg of **7** and 8 mg of **2**. The data for these compounds were previously reported in the literature.[9,10]

9,10-diphenylanthracene (7)

Physical Property: White solid.

Isolated Yield: 30 %.

TLC: R_f = 0.79 (silica gel, ethyl acetate/hexane 1:4).

^{1}H NMR (500 MHz, $CDCl_3$)

δ 7.72-7.68 (m, 4H), 7.63-7.53 (m, 6H), 7.51-7.47 (m, 4H), 7.35-7.31 (m, 4H).

1,4-diphenylphthalazine (2)

Physical Property: White solid.

Isolated Yield: 28 %.

TLC: R_f = 0.11 (silica gel, silica gel, ethyl acetate/hexane 1:4).

^{1}H NMR (500 MHz, $CDCl_3$)

δ 8.17-8.12 (m, 4H), 7.87-7.79 (m, 6H), 7.62-7.54 (m, 6H).

References

1 J. Yang, M. R. Karver, W. Li, S. Sahu and N. K. Devaraj, Angew. Chem. 2012, *Angew. Chem. Int. Ed.* **2012**, *51*, 5222–5225.

2 R. A. Carboni and R. V. Lindsey. Jr., *J. Am. Chem. Soc.* **1959**, *81*, 4342–4346.

3 W. Skorianetz and E. Sz. Kováts, *Helv. Chim. Acta* **1971**, *54*, 1922–1939.

4 A. Counotte-Potman, H. C. van der Plas and B. van Veldhuizen, *J. Org. Chem.* **1981**, *46*, 2138–2141.

5 S. J. Weininger E. R. Thornton, *J. Am. Chem. Soc.* **1967**, *89*, 2050–2054.

6 W. X. Hu and F. Xu, *J. Heterocycl. Chem.* **2008**, *45*, 1745–1750.

7 A. T. Biju and F. Glorius, *Angew. Chem. Int. Ed.* **2010**, *49*, 9761–9764.

8 D. Bhattacharjee and F. D. Popp, *J. Heterocycl. Chem.* **1980**, *17*, 433–437

9 E. Yagodkin and C. J. Douglas, *Tetrahedron Lett.* **2010**, *51*, 3037–3040.

10 J. Li, J. Gao, W.-W. Xiong and Q. Zhang, *Tetrahedron Lett.* **2014**, *55*, 4346–4349.

^{1}H NMR spectrum of **2** in $CDCl_3$ (500 MHz).

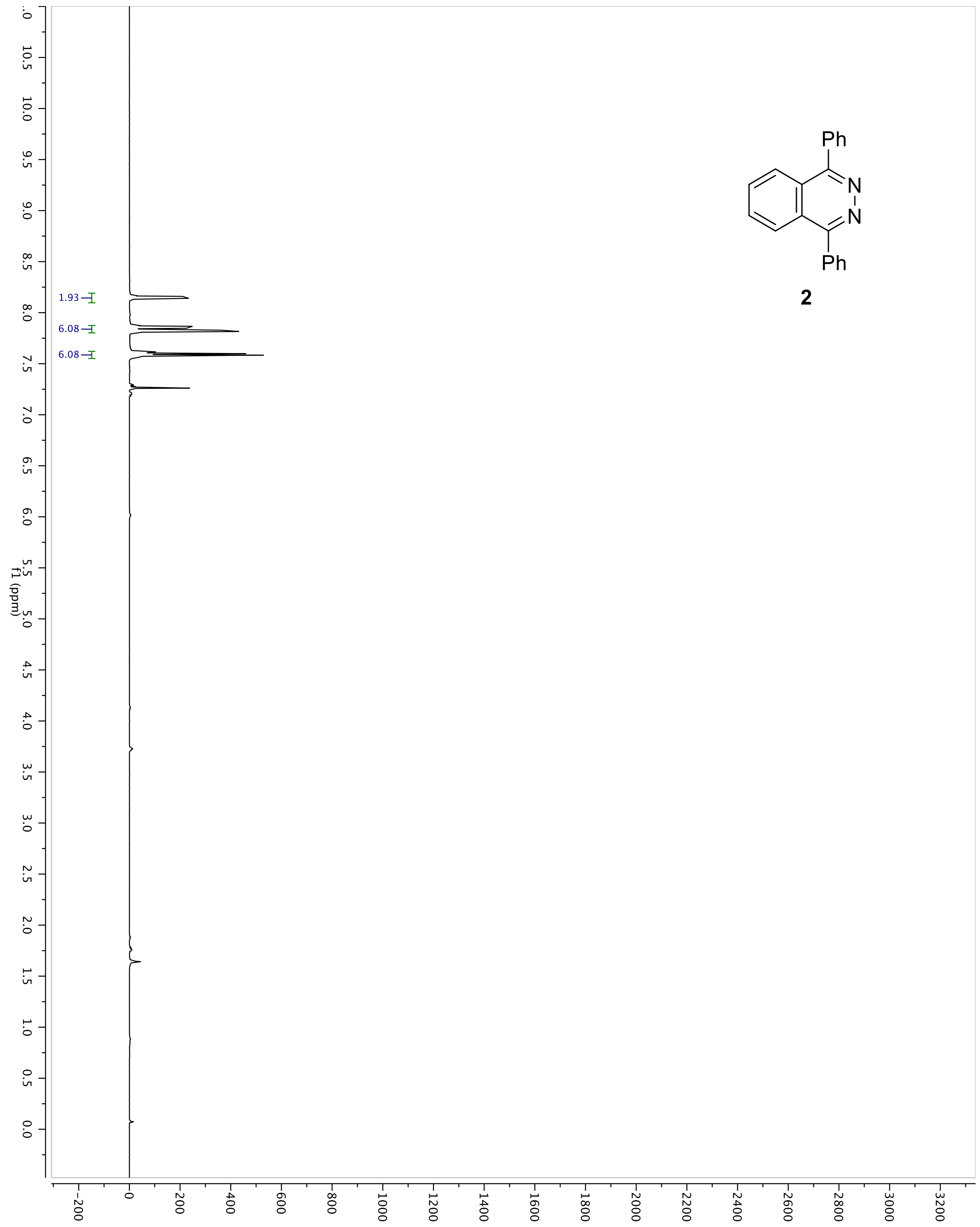

^{1}H NMR spectrum of **3a** in $CDCl_3$ (500 MHz).

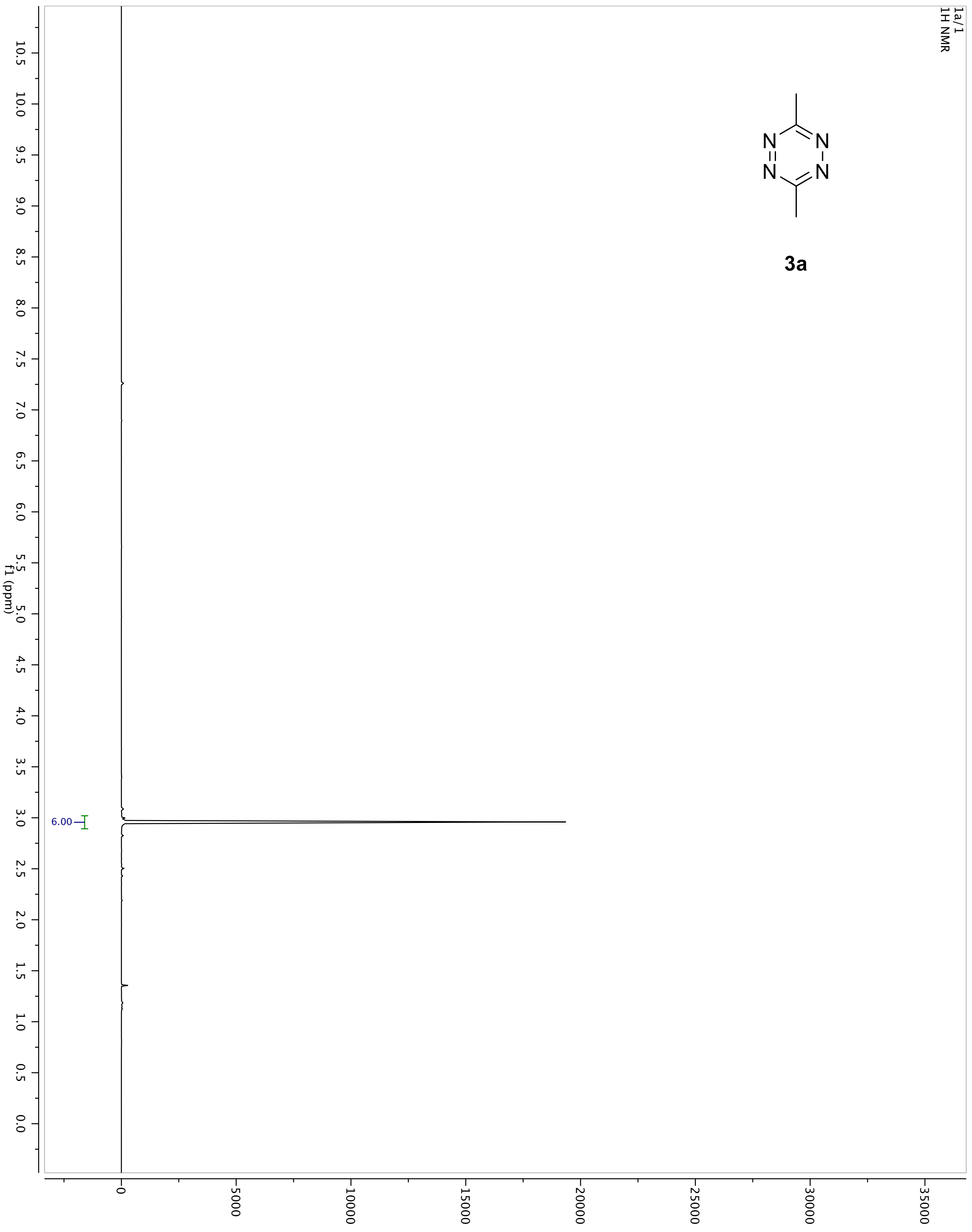

^{13}C NMR spectrum of **3a** in $CDCl_3$ (125 MHz).

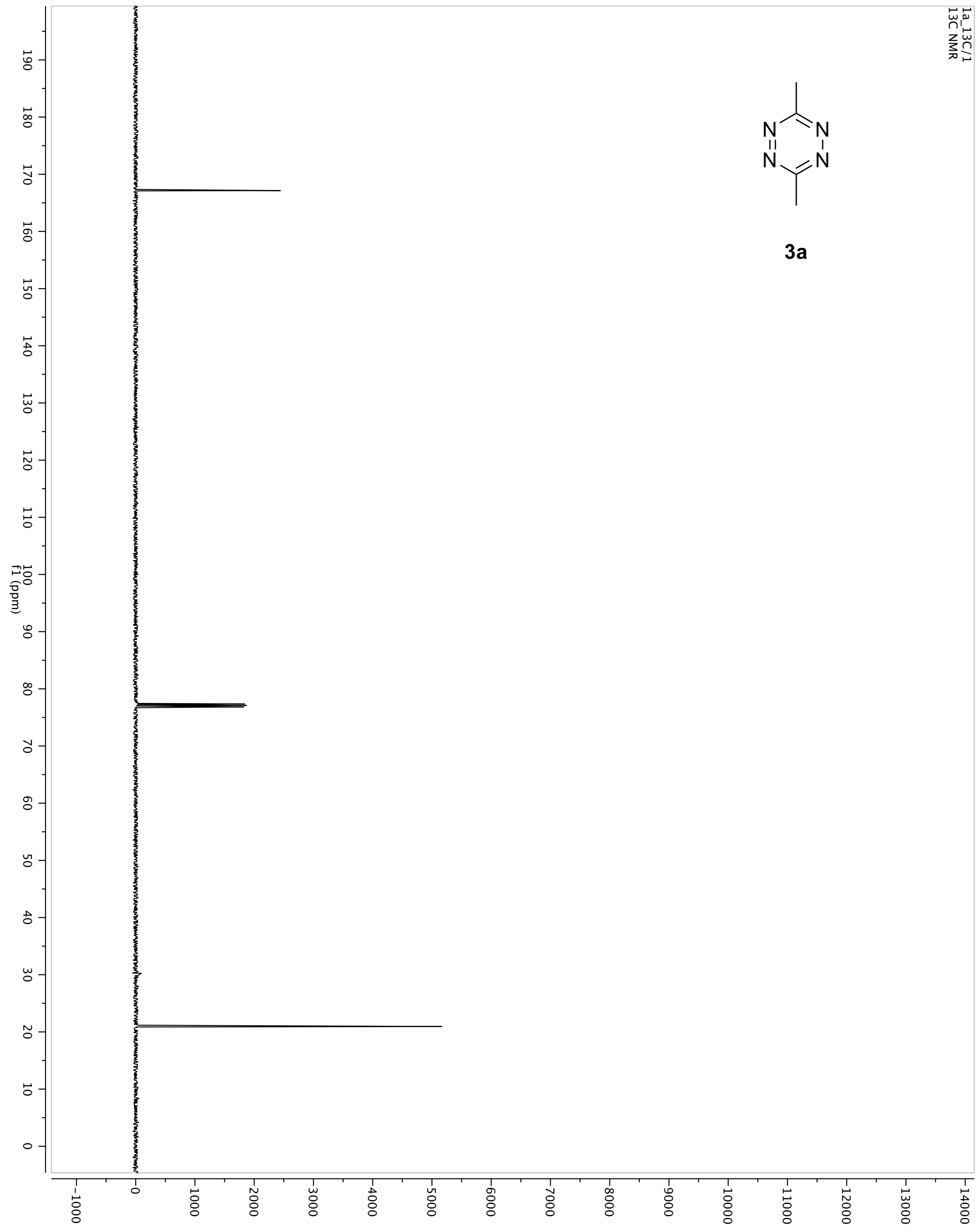

^{1}H NMR spectrum of **3b** in $CDCl_3$ (500 MHz).

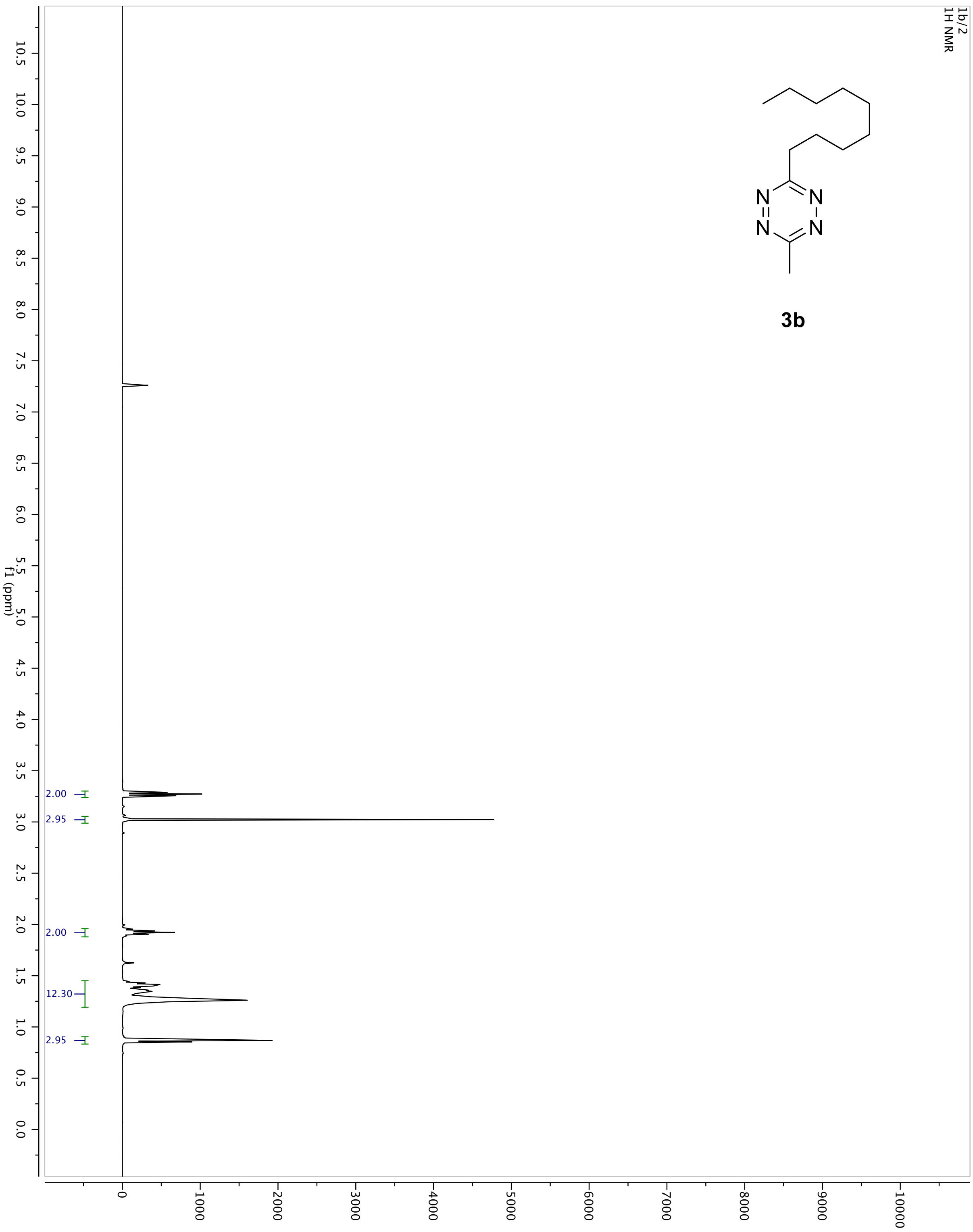

^{13}C NMR spectrum of **3b** in $CDCl_3$ (125 MHz).

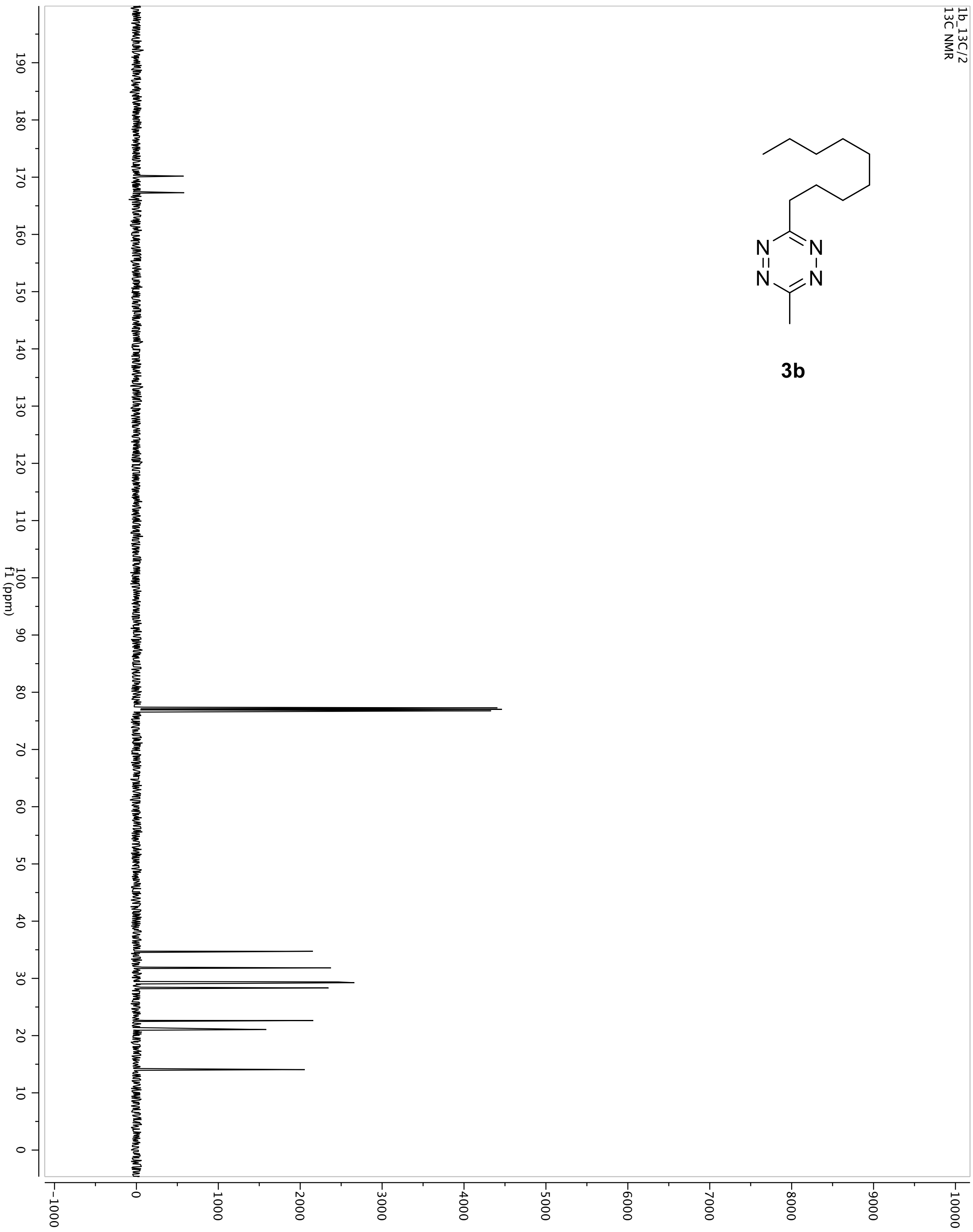

^{1}H NMR spectrum of **3c** in $CDCl_3$ (500 MHz).

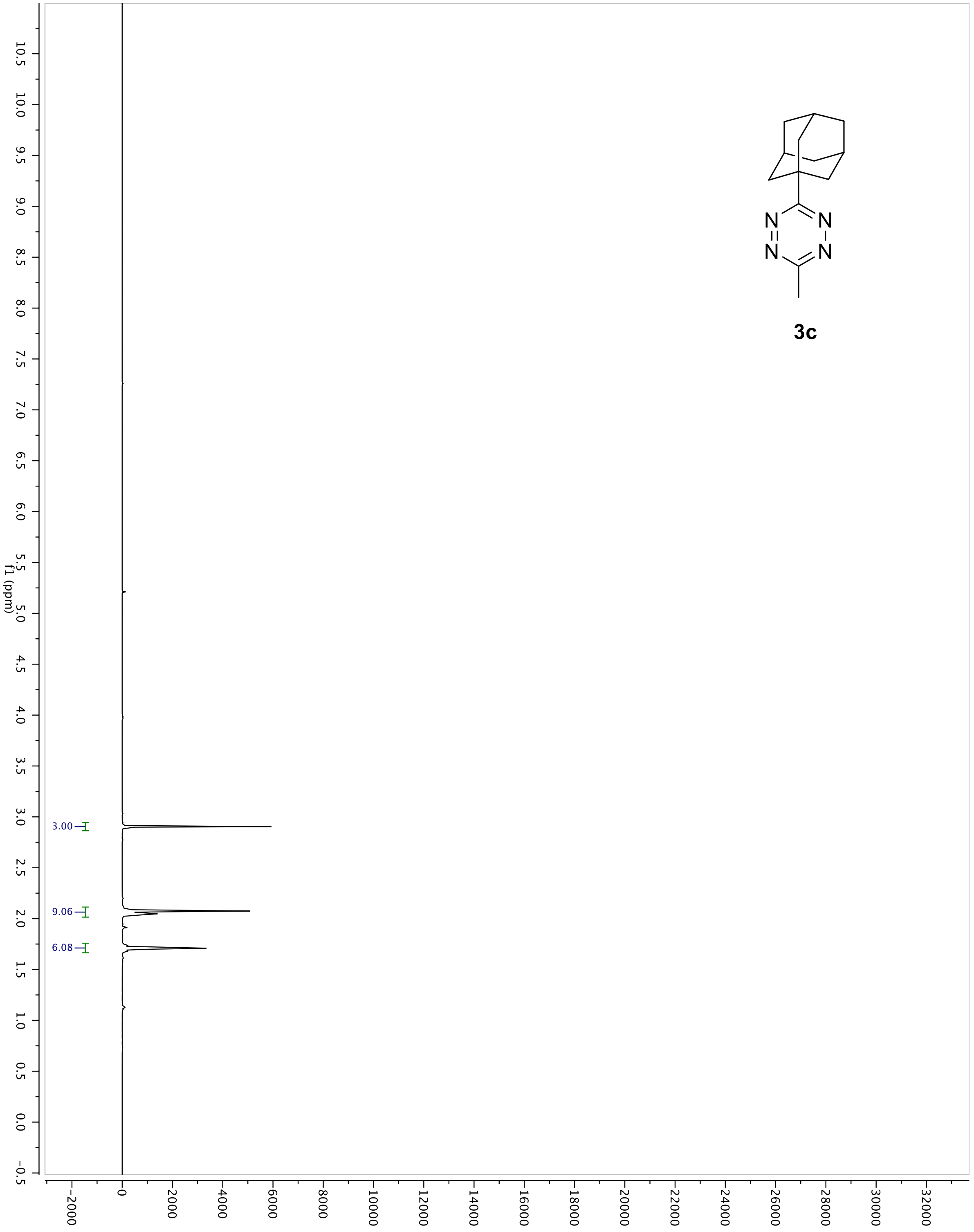

^{13}C NMR spectrum of **3c** in $CDCl_3$ (125 MHz).

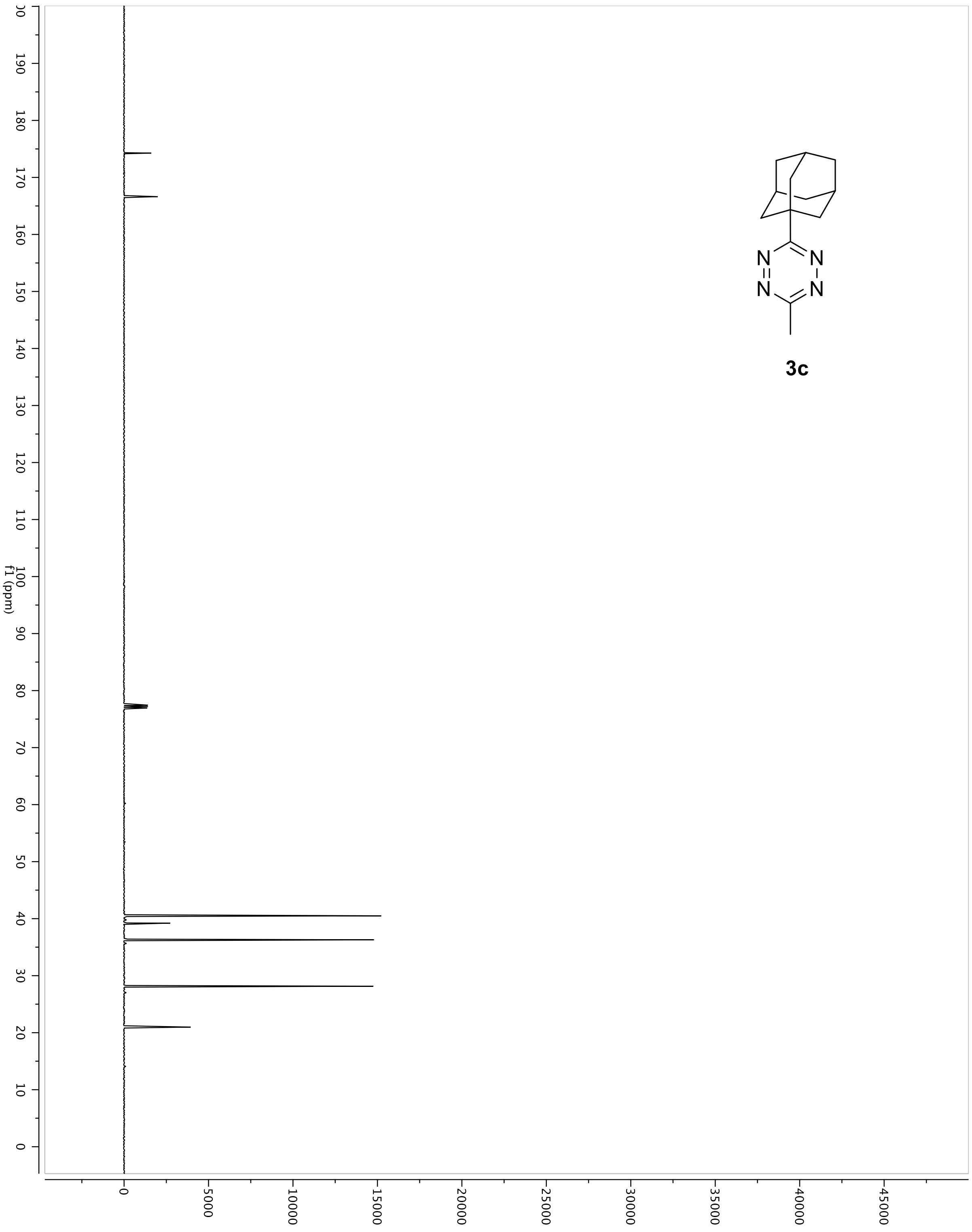

^{1}H NMR spectrum of **3d** in $CDCl_3$ (500 MHz).

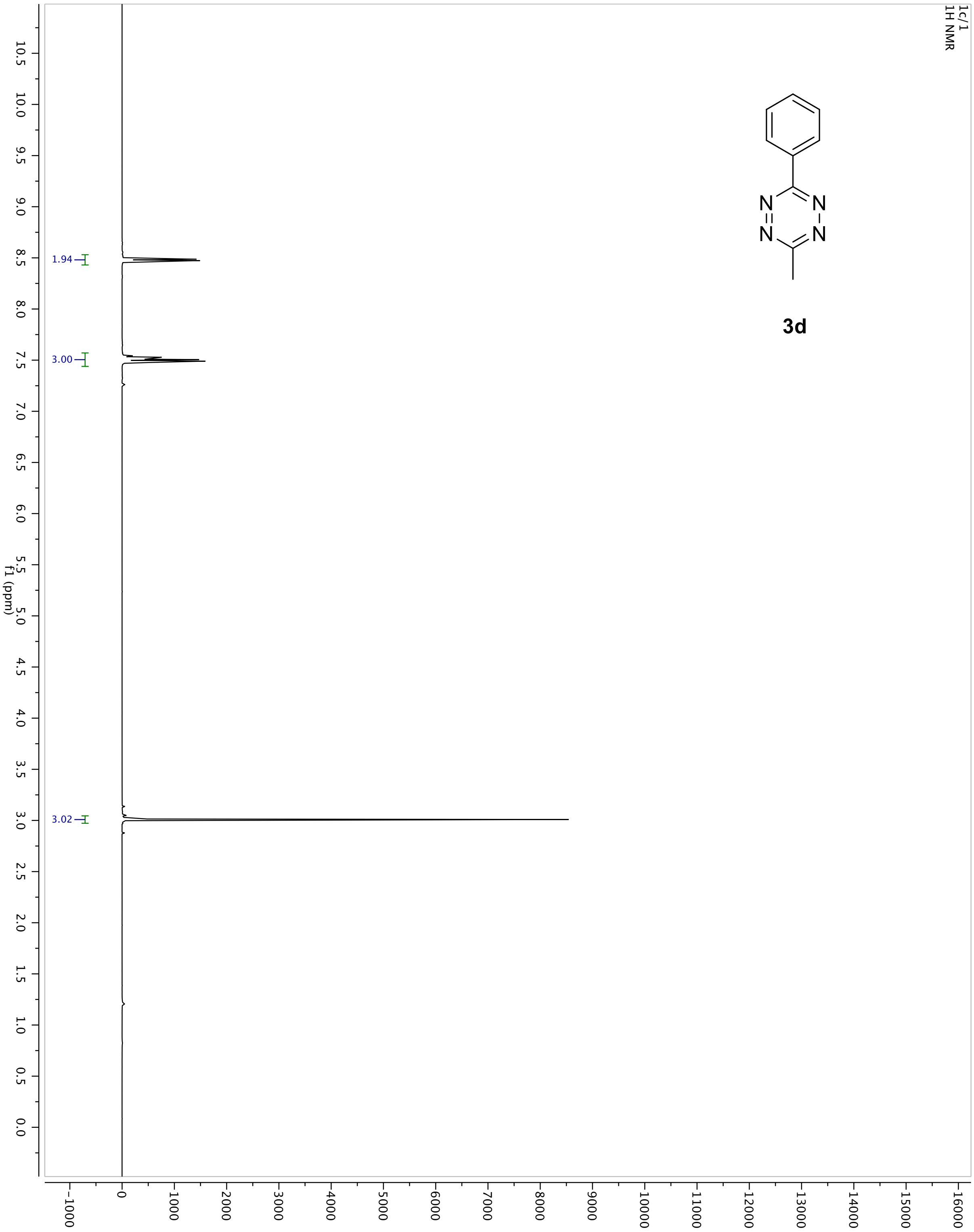

^{13}C NMR spectrum of **3d** in $CDCl_3$ (125 MHz).

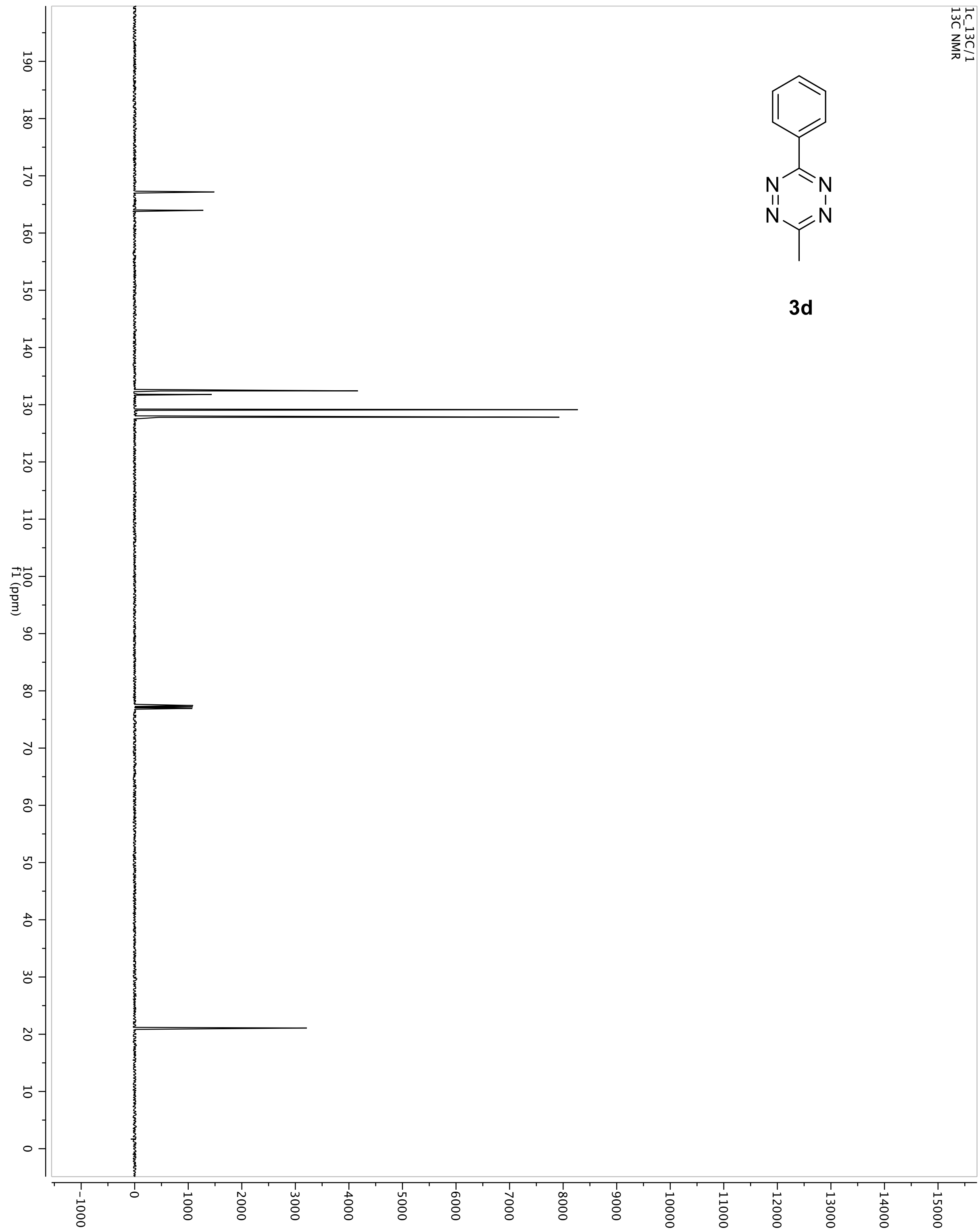

^{1}H NMR spectrum of **3e** in $CDCl_3$ (500 MHz).

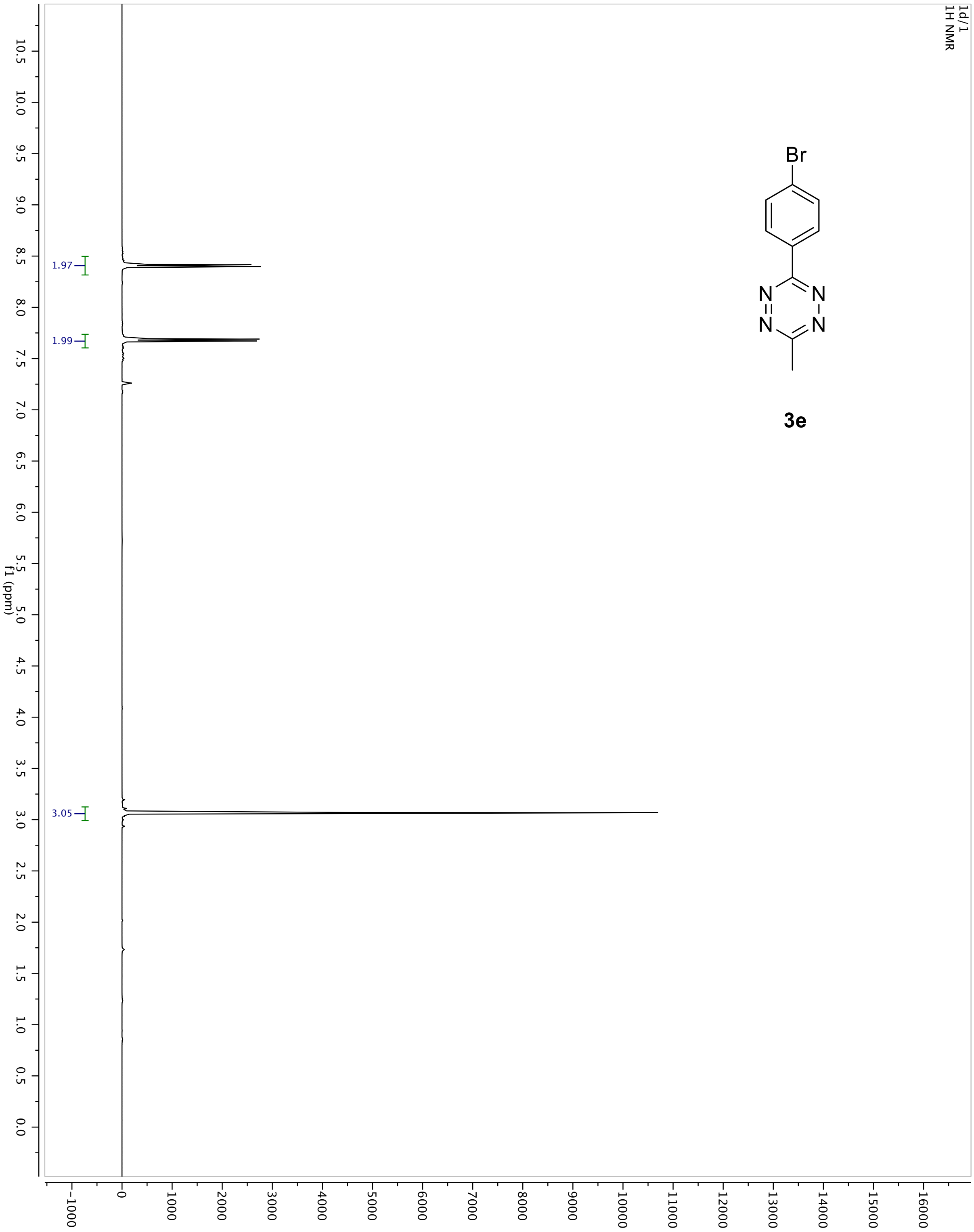

^{13}C NMR spectrum of **3e** in $CDCl_3$ (125 MHz).

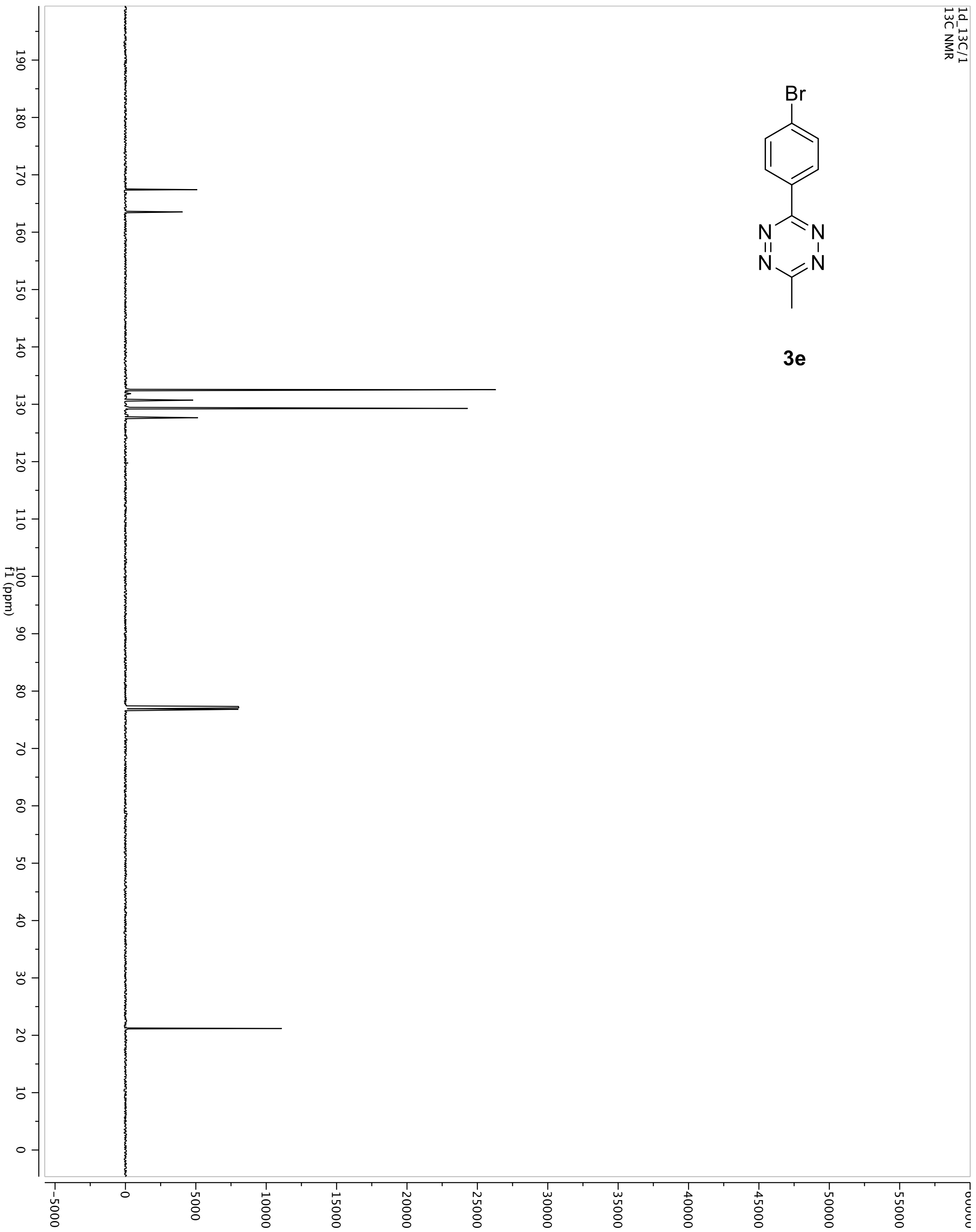

^{1}H NMR spectrum of **3f** in $CDCl_3$ (500 MHz).

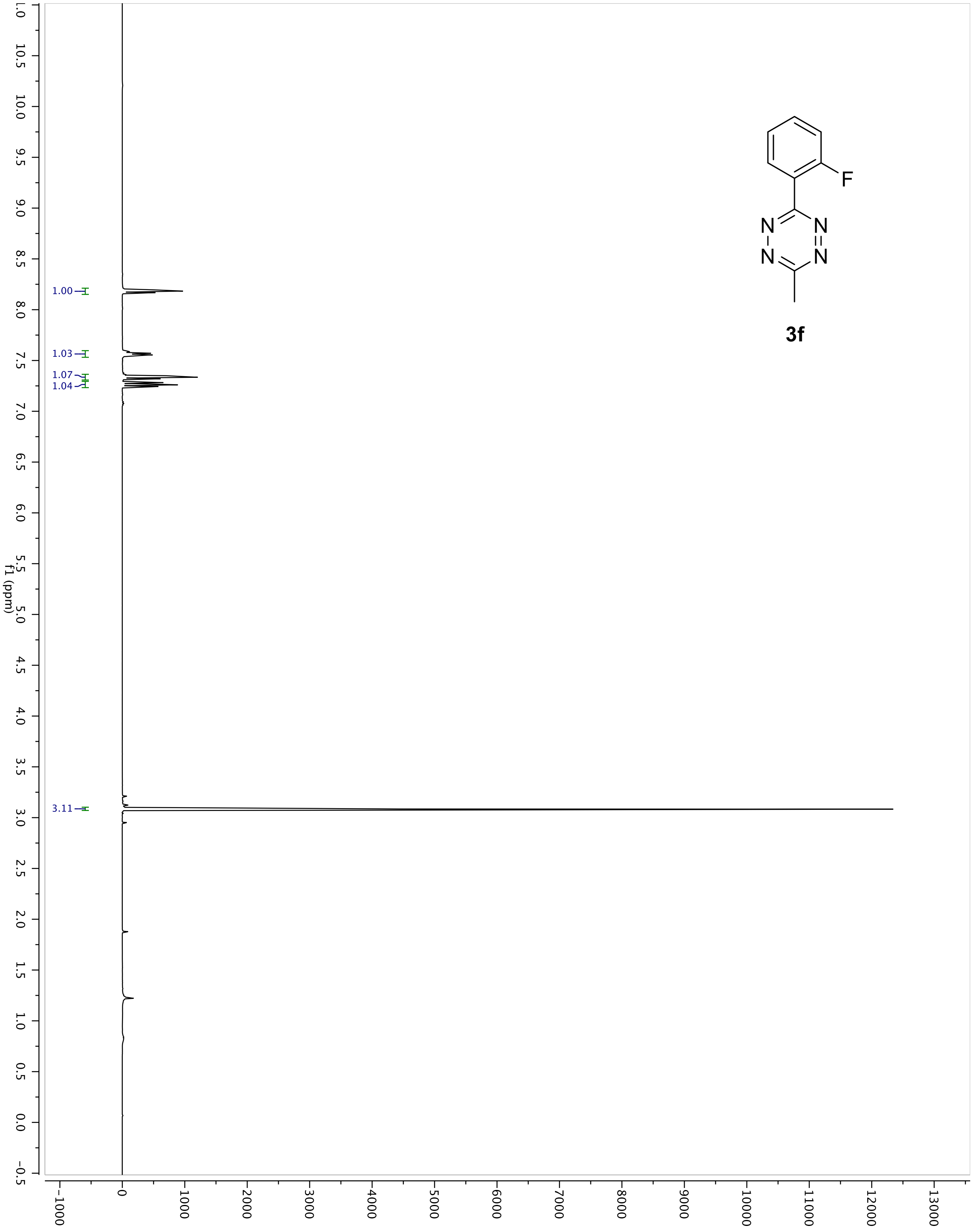

^{13}C NMR spectrum of **3f** in $CDCl_3$ (125 MHz).

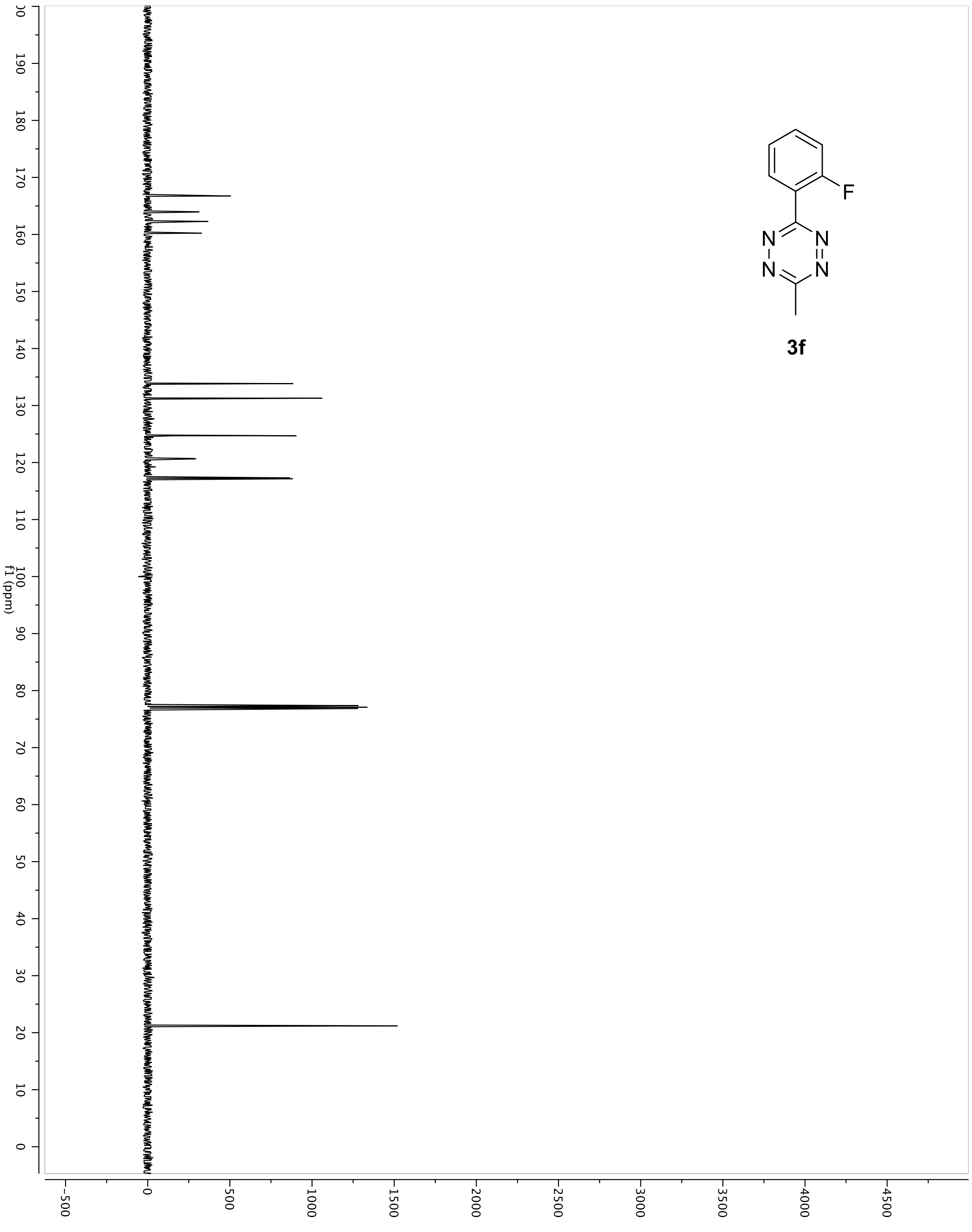

^{19}F NMR spectrum of **3f** in $CDCl_3$ (338 MHz).

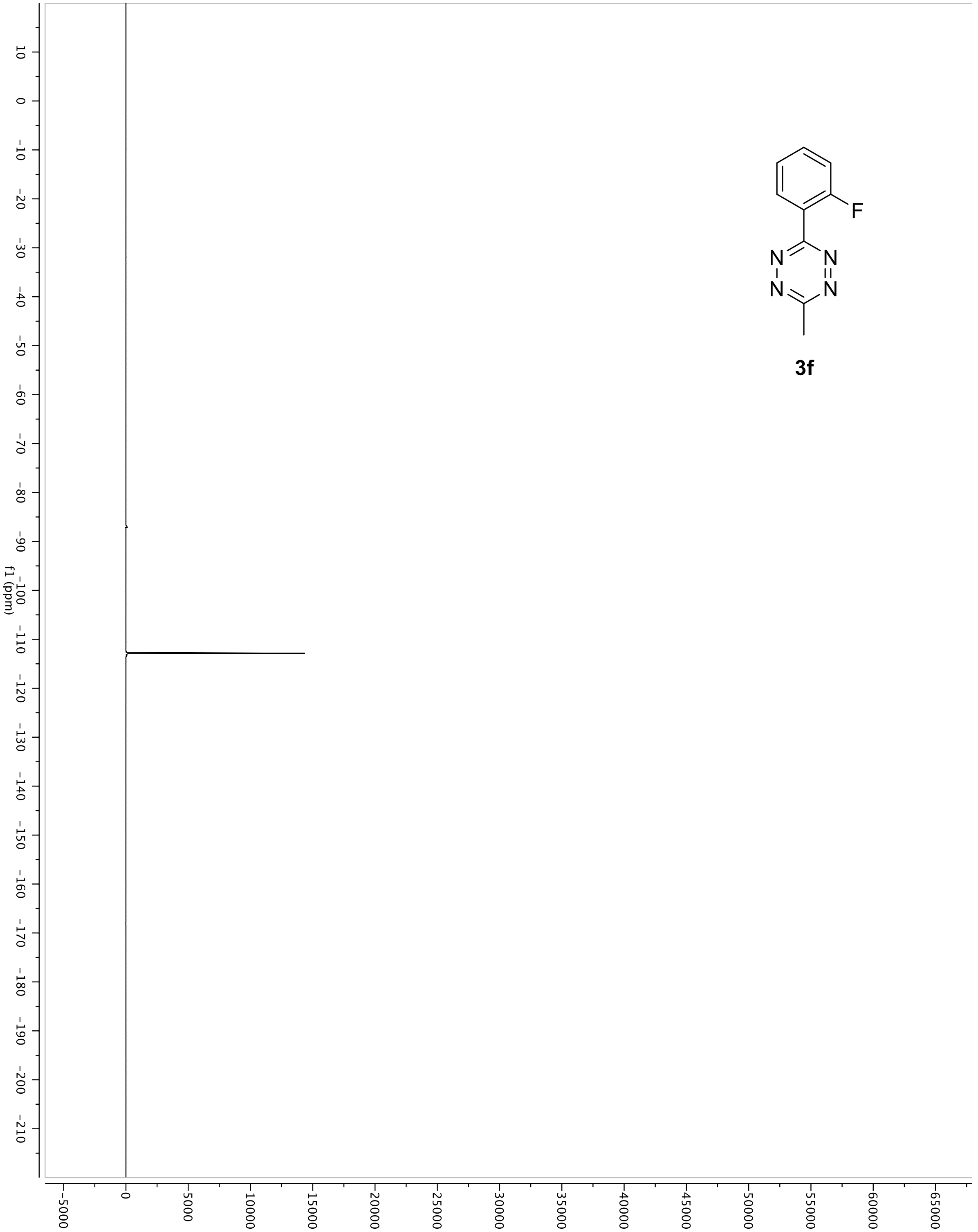

^{1}H NMR spectrum of **3g** in $CDCl_3$ (500 MHz).

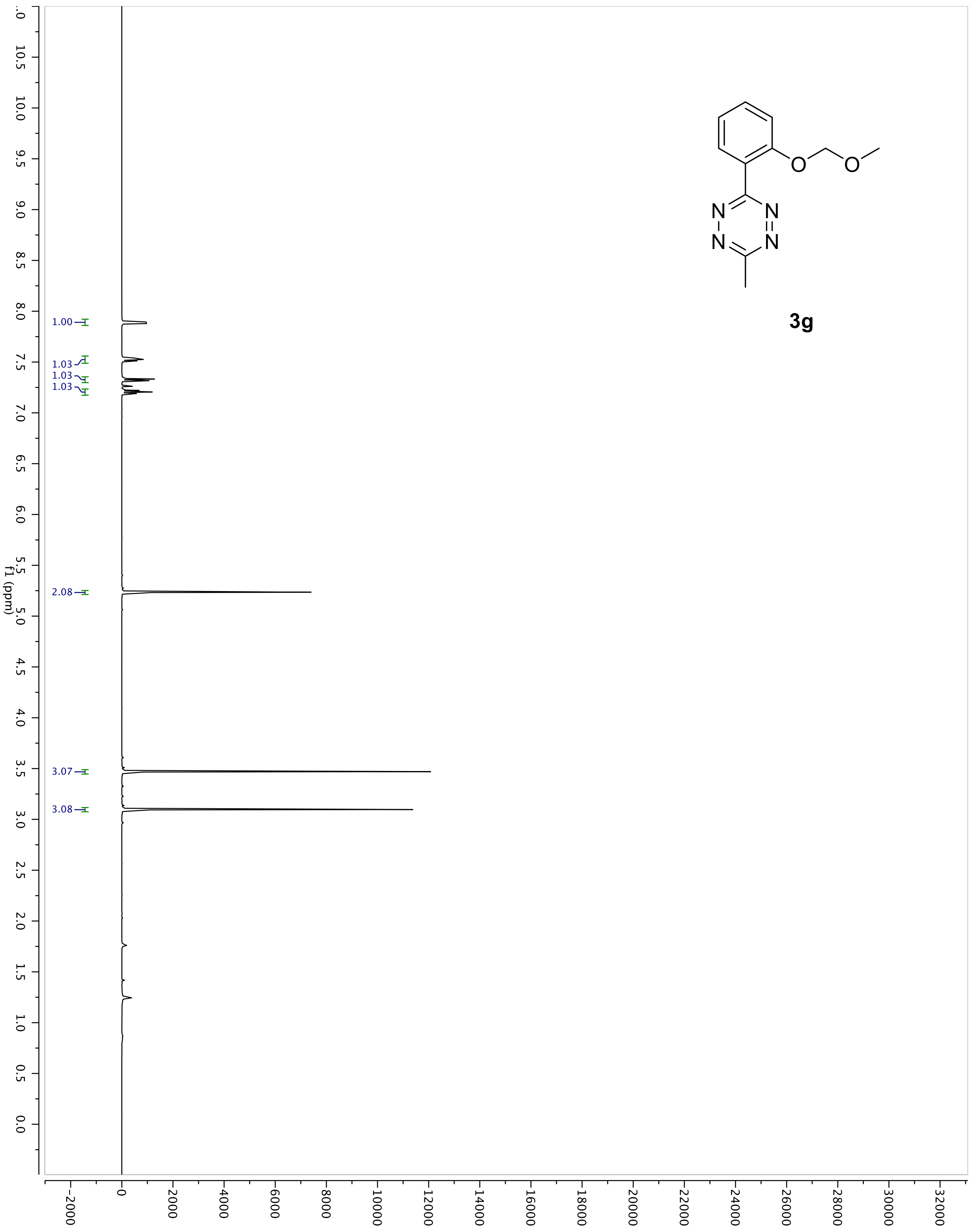

^{13}C NMR spectrum of **3g** in $CDCl_3$ (125 MHz).

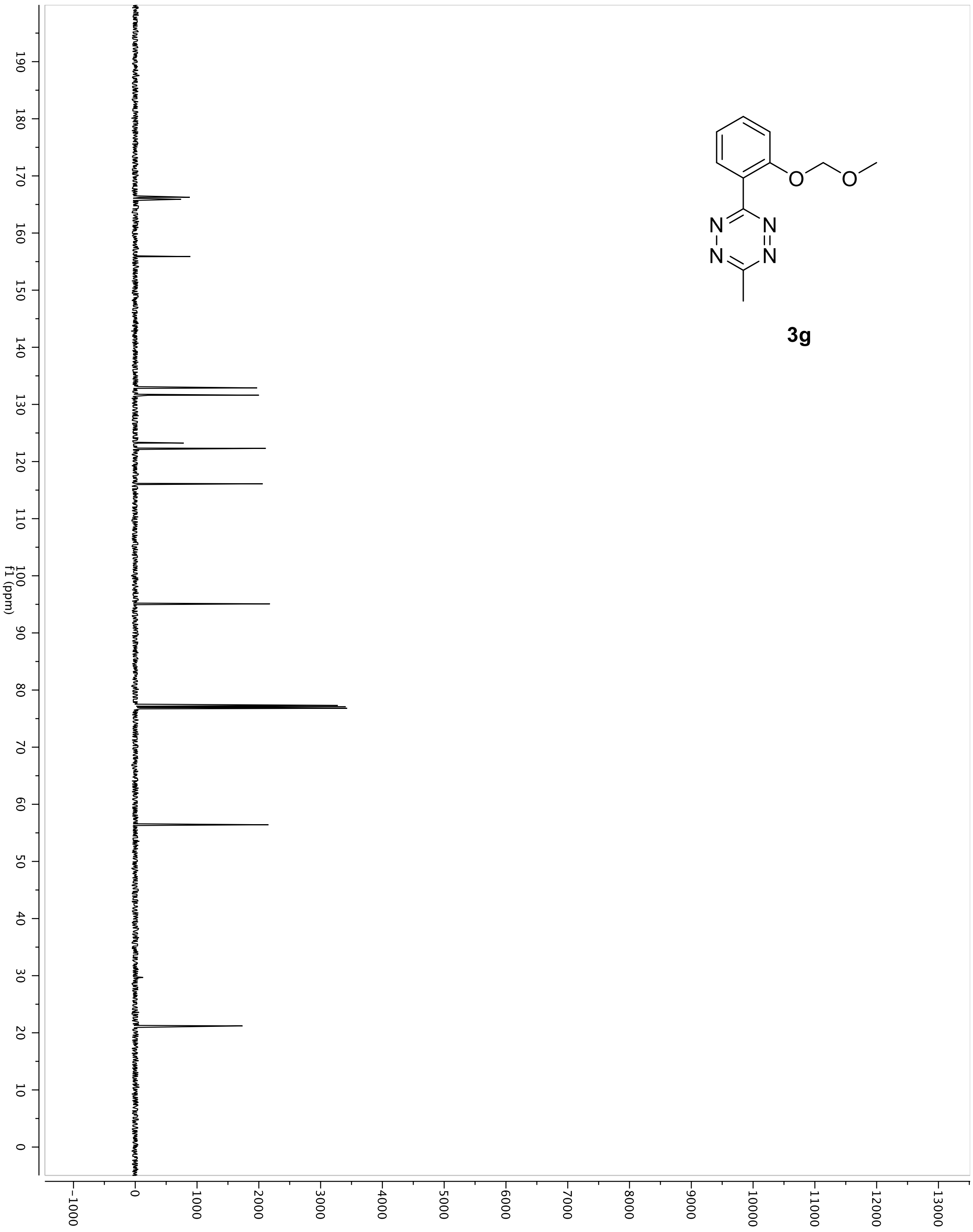

^{1}H NMR spectrum of **3h** in $CDCl_3$ (500 MHz).

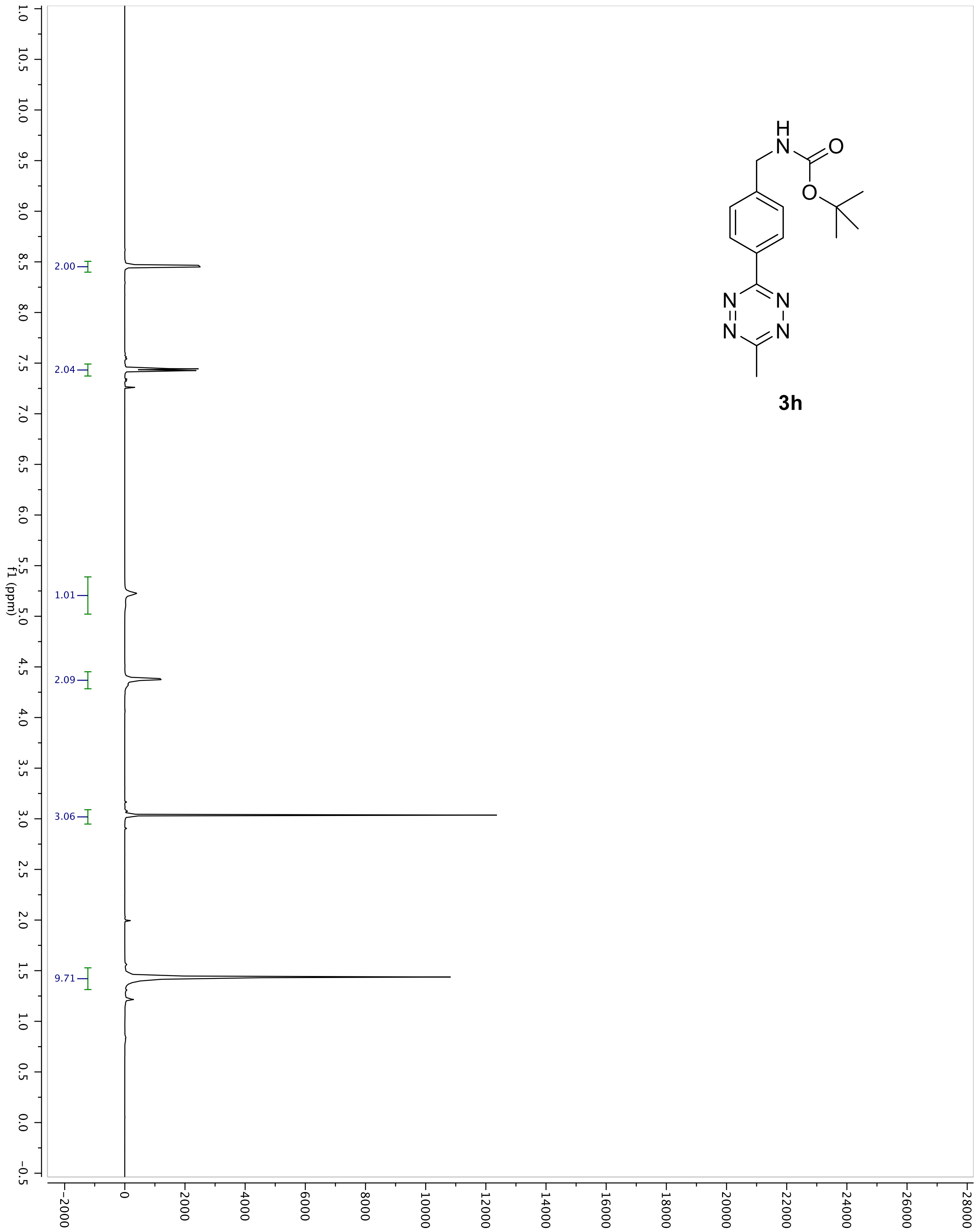

^{1}H NMR spectrum of **3i** in $CDCl_3$ (500 MHz).

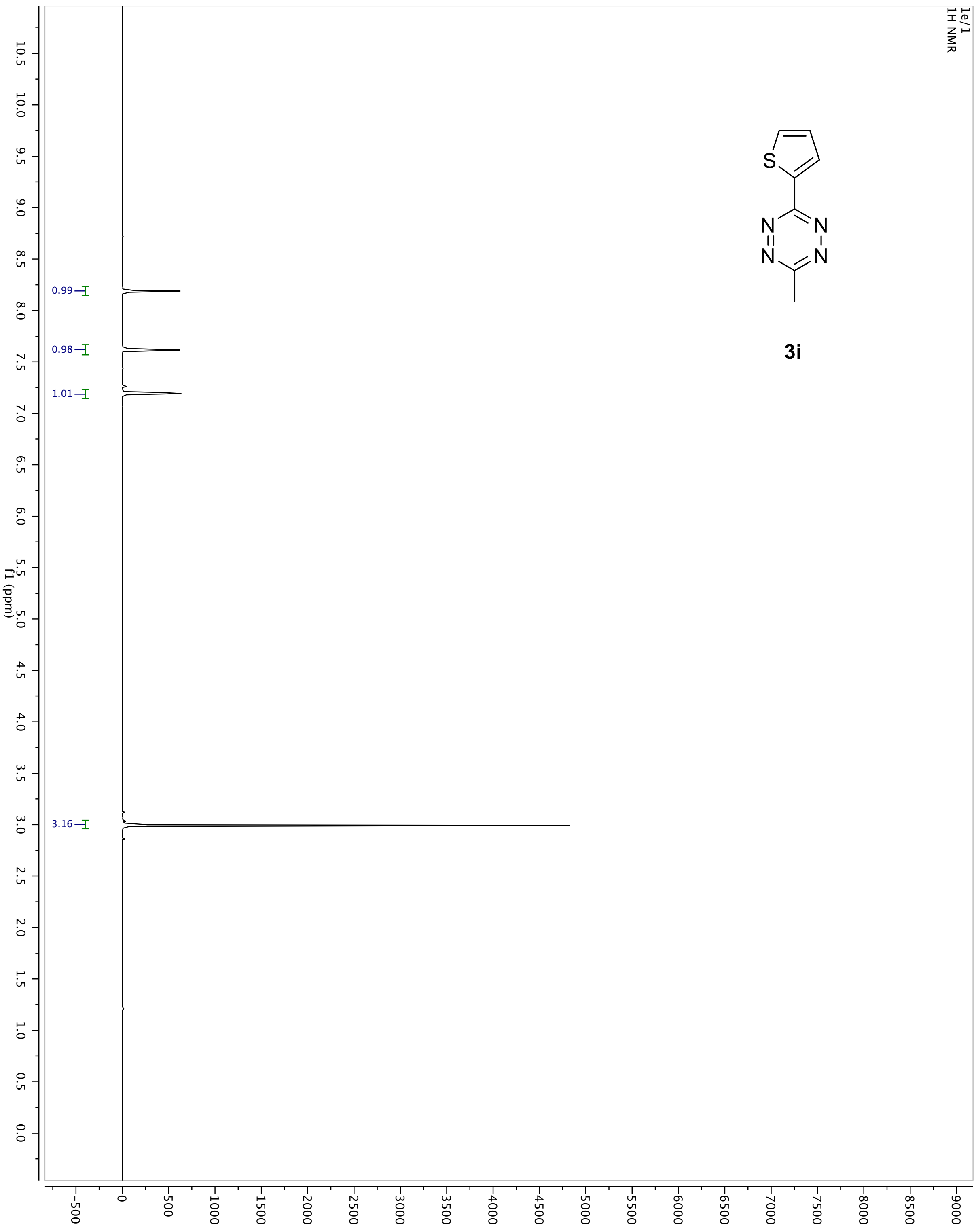

^{13}C NMR spectrum of **3i** in $CDCl_3$ (125 MHz).

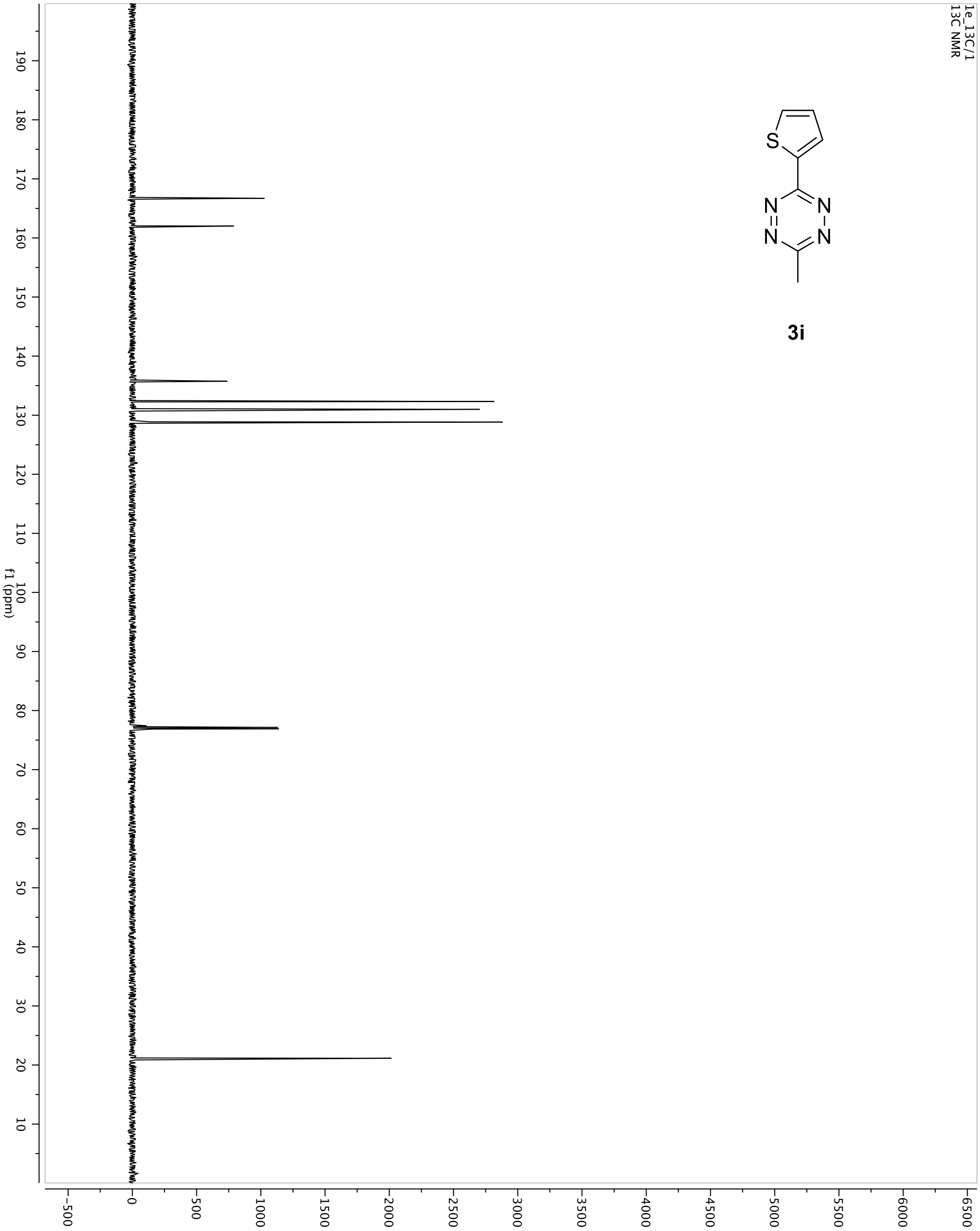

^{1}H NMR spectrum of **3j** in $CDCl_3$ (500 MHz).

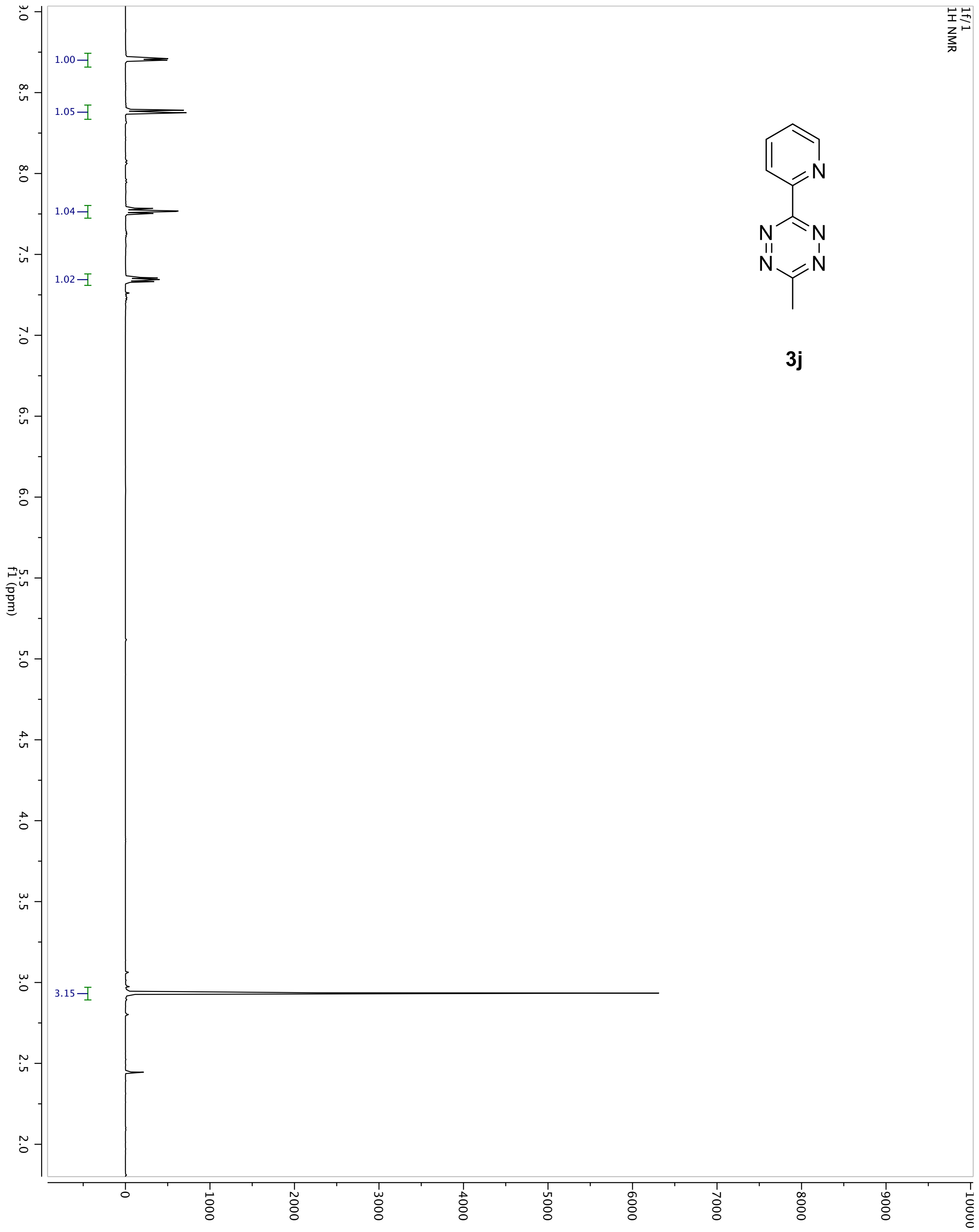

^{13}C NMR spectrum of **3j** in $CDCl_3$ (125 MHz).

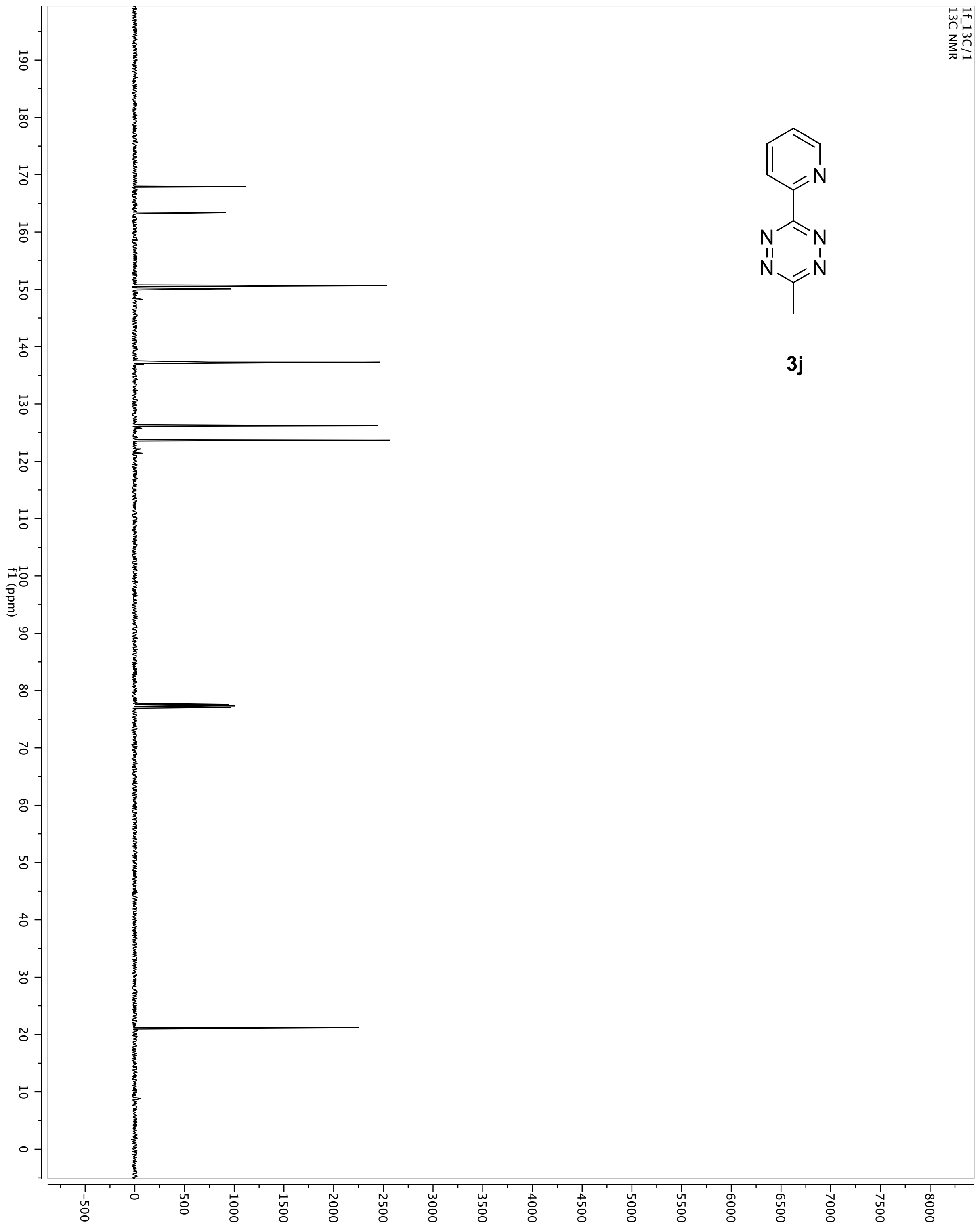

^{1}H NMR spectrum of **3k** in $CDCl_3$ (500 MHz).

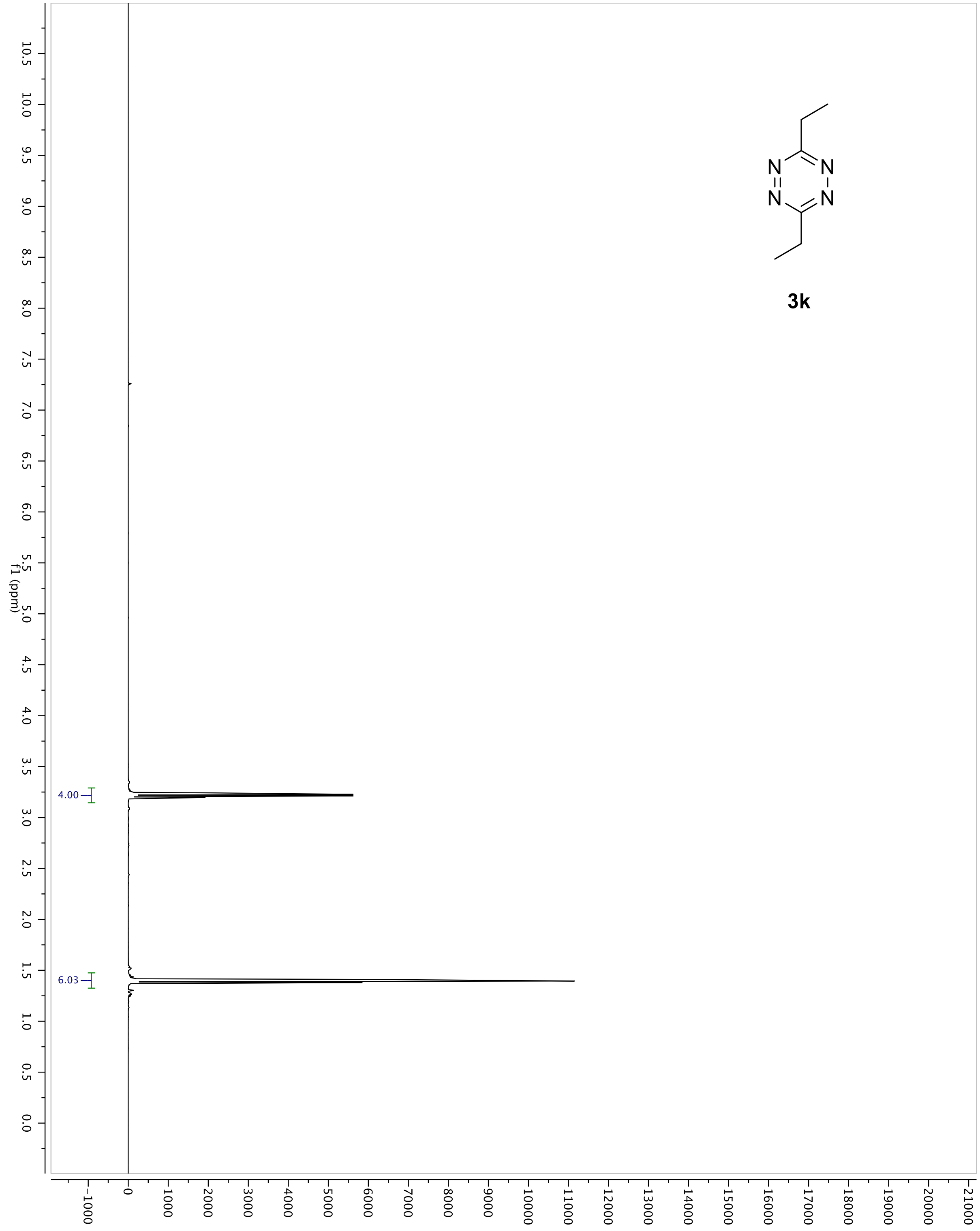

^{13}C NMR spectrum of **3k** in $CDCl_3$ (125 MHz).

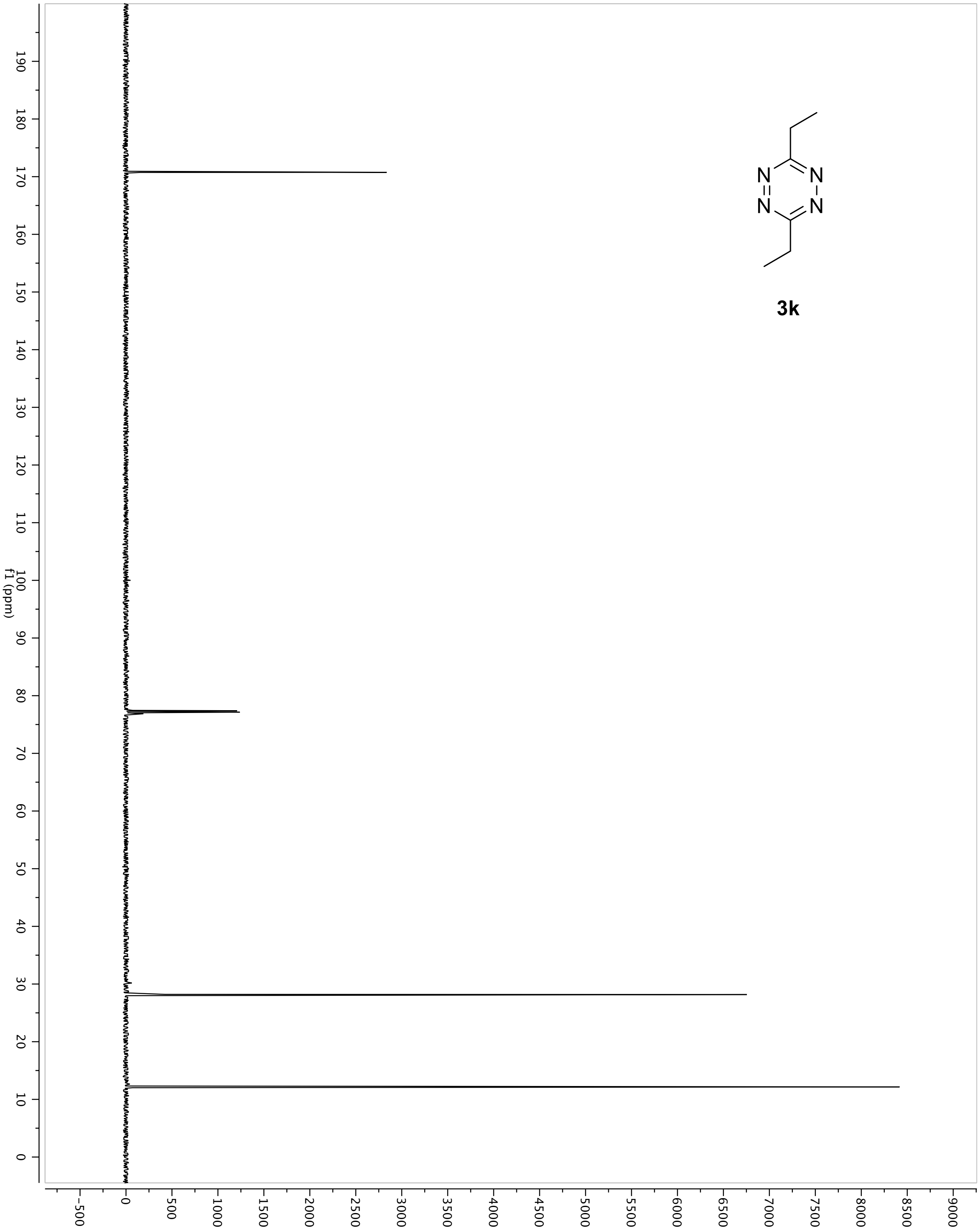

^{1}H NMR spectrum of **3l** in $CDCl_3$ (500 MHz).

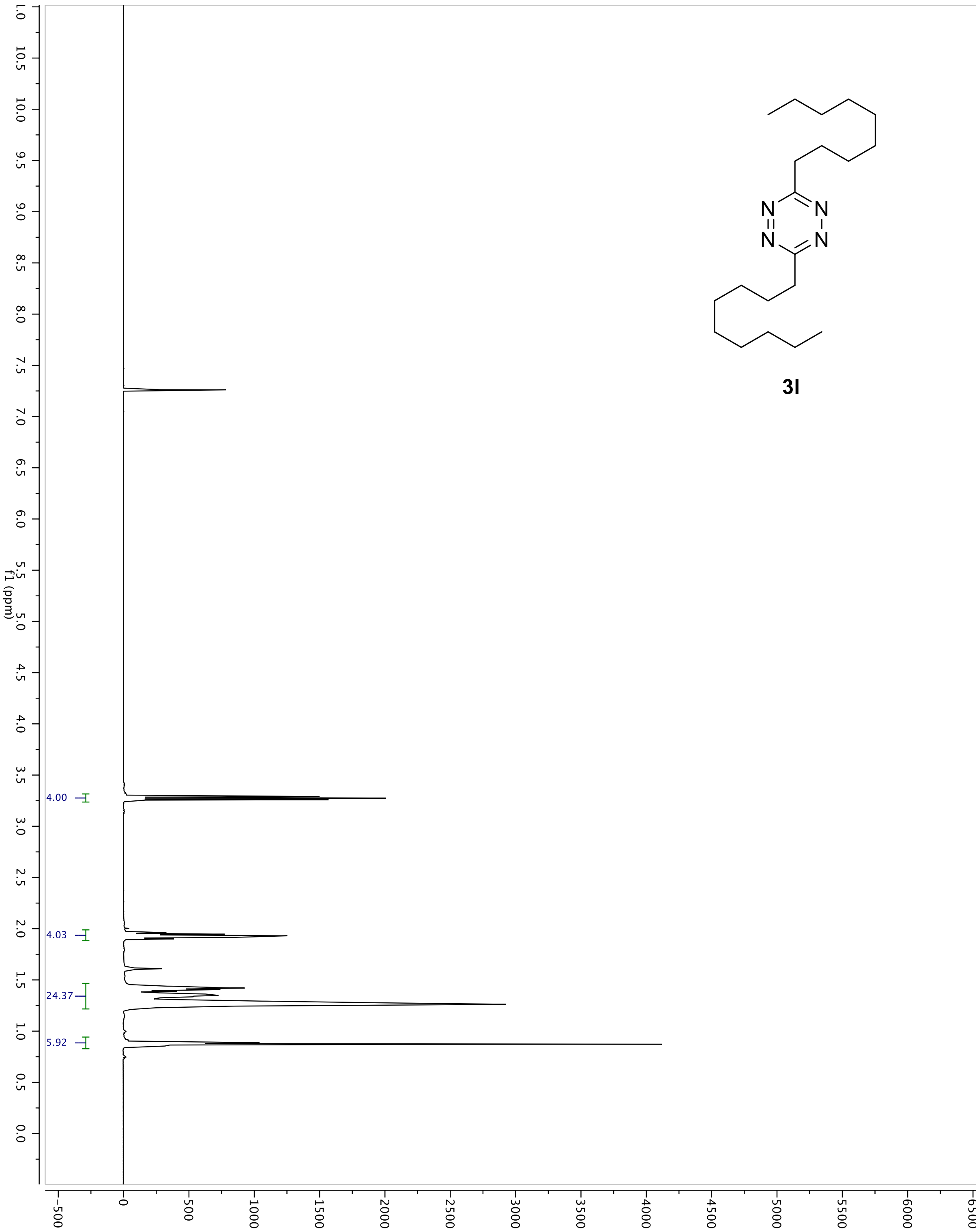

^{13}C NMR spectrum of **3l** in $CDCl_3$ (125 MHz).

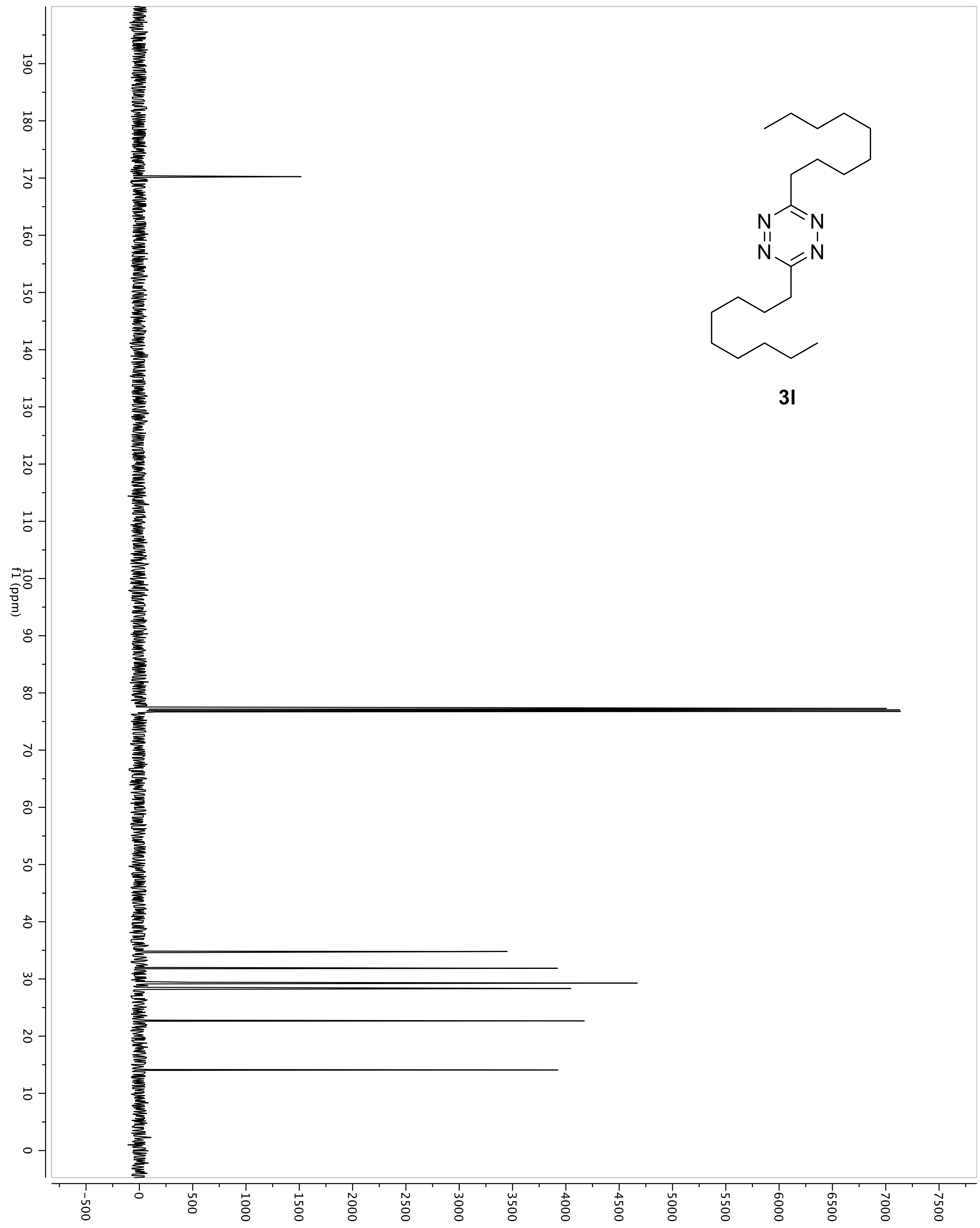

^1H NMR spectrum of **3m** in $CDCl_3$ (500 MHz).

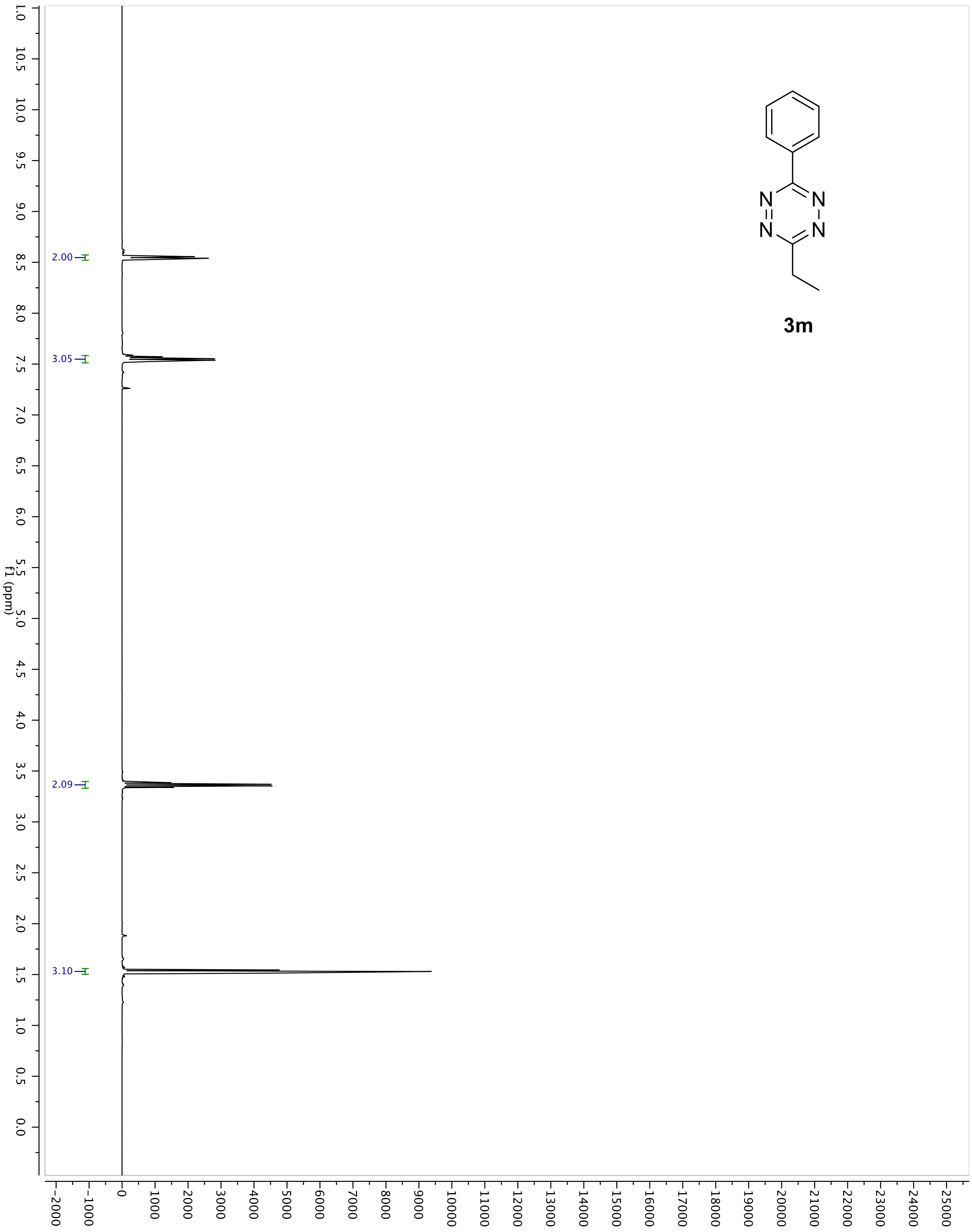

^{13}C NMR spectrum of **3m** in $CDCl_3$ (125 MHz).

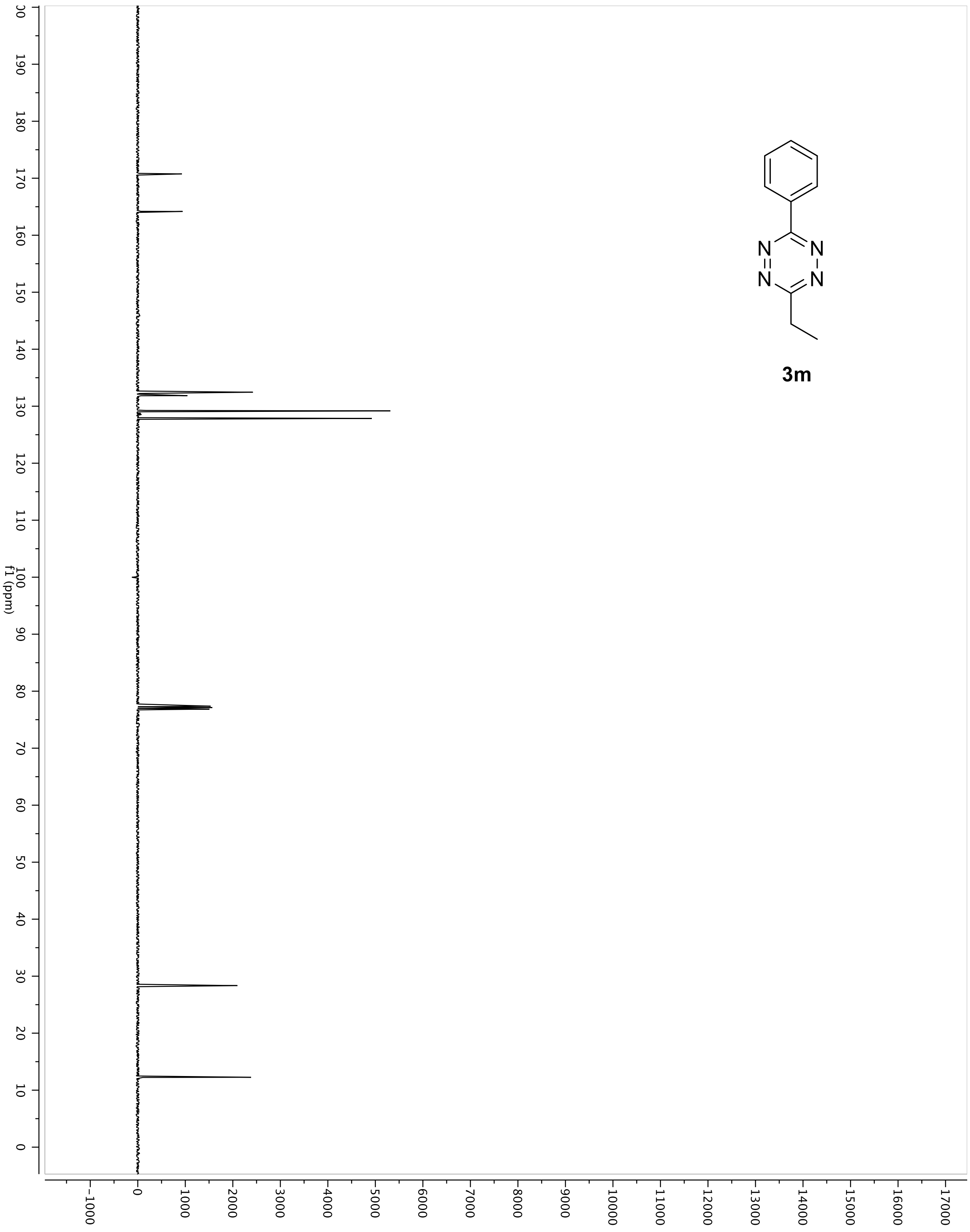

^{1}H NMR spectrum of **3n** in $CDCl_3$ (500 MHz).

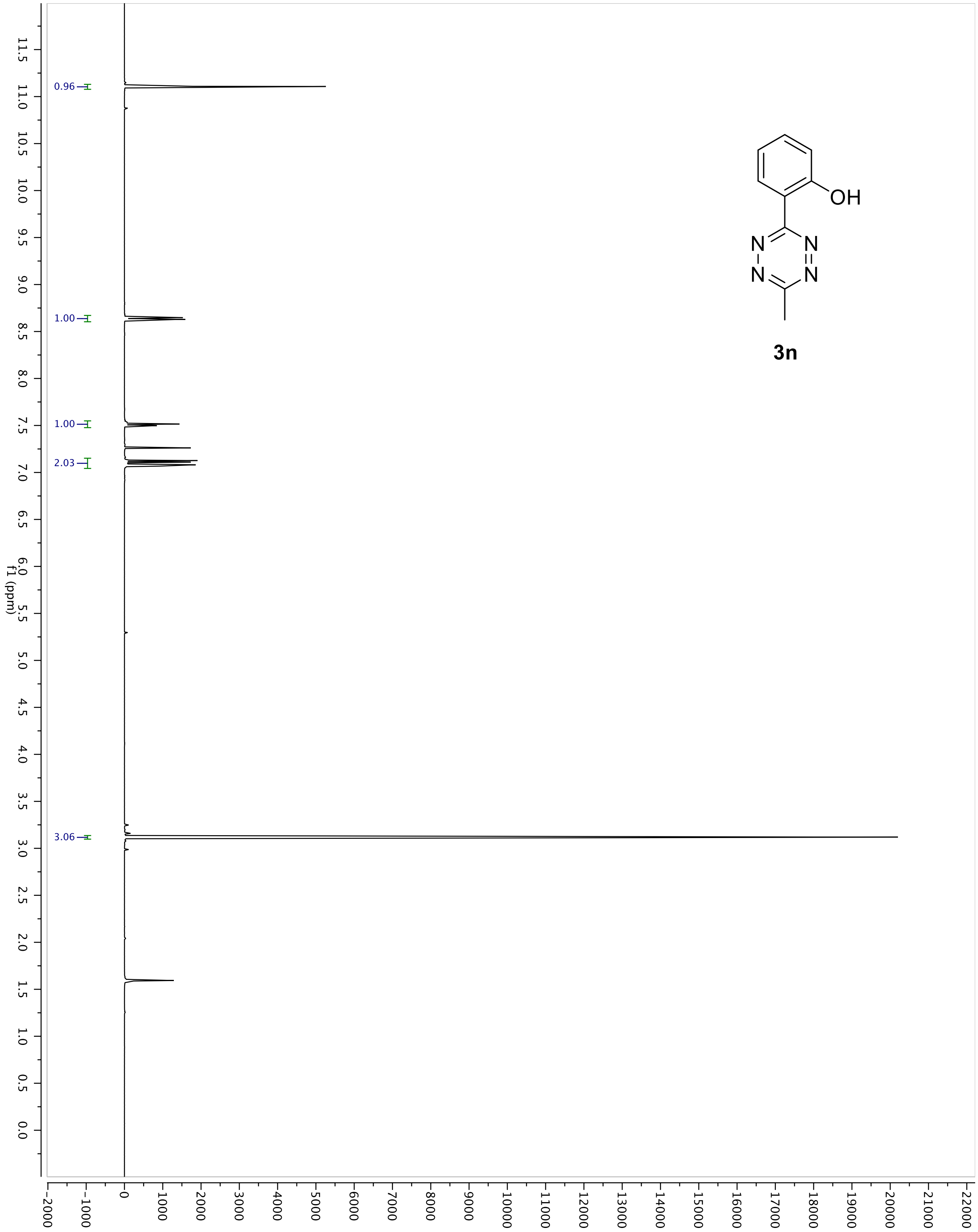

13C NMR spectrum of **3n** in $CDCl_3$ (125 MHz).

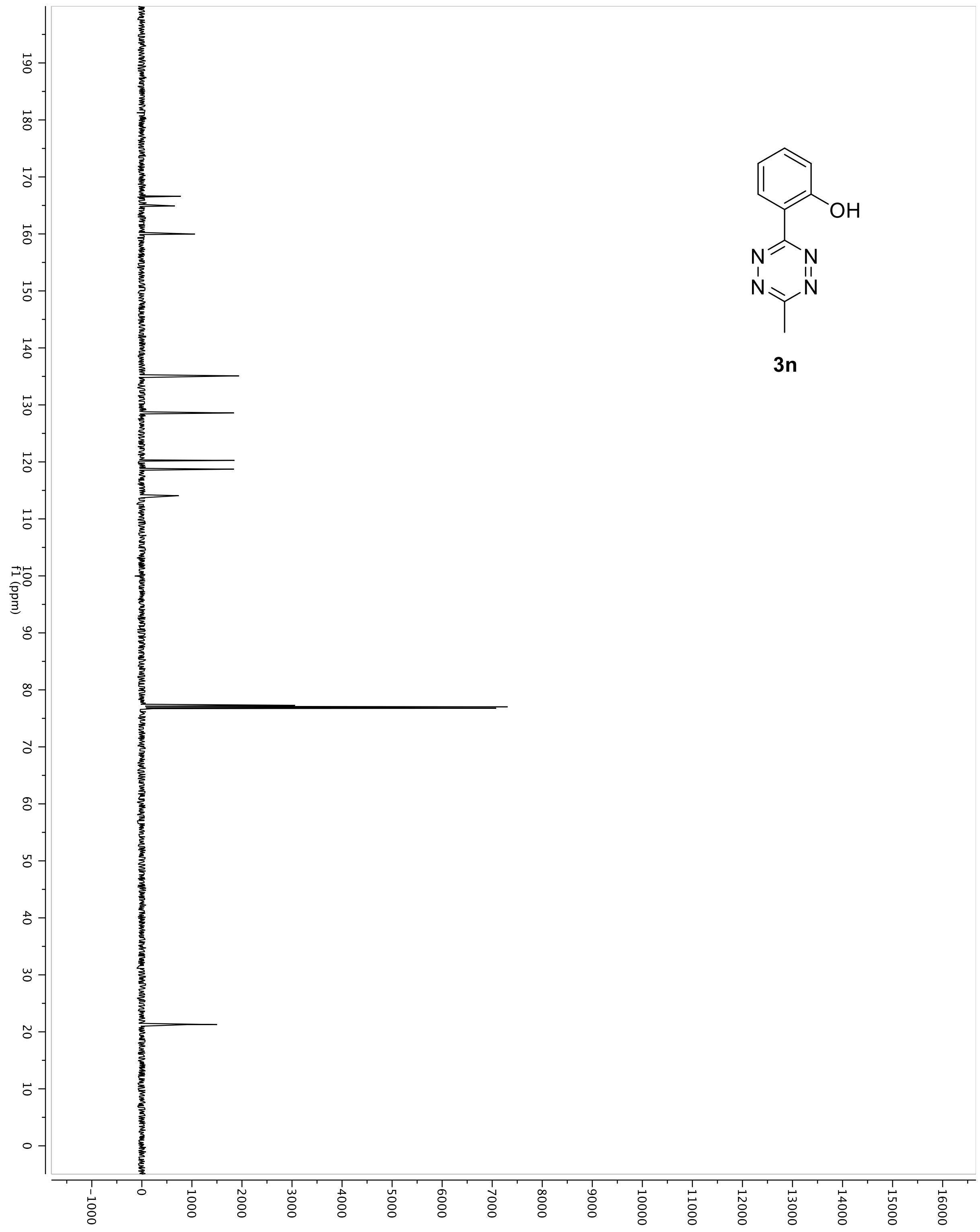

^{1}H NMR spectrum of **4b** in $CDCl_3$ (500 MHz).

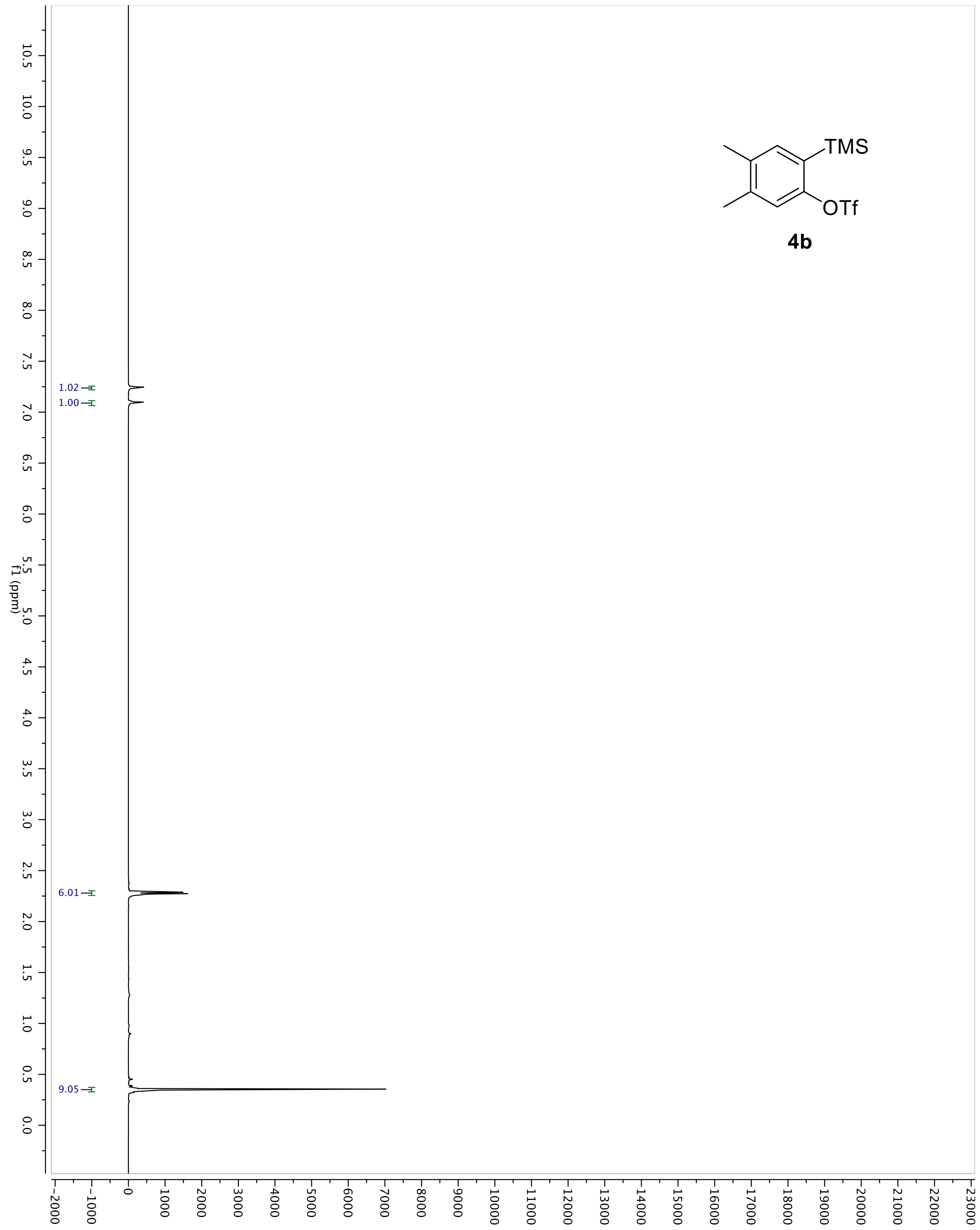

^{1}H NMR spectrum of **5a** in CD_2Cl_2 (500 MHz).

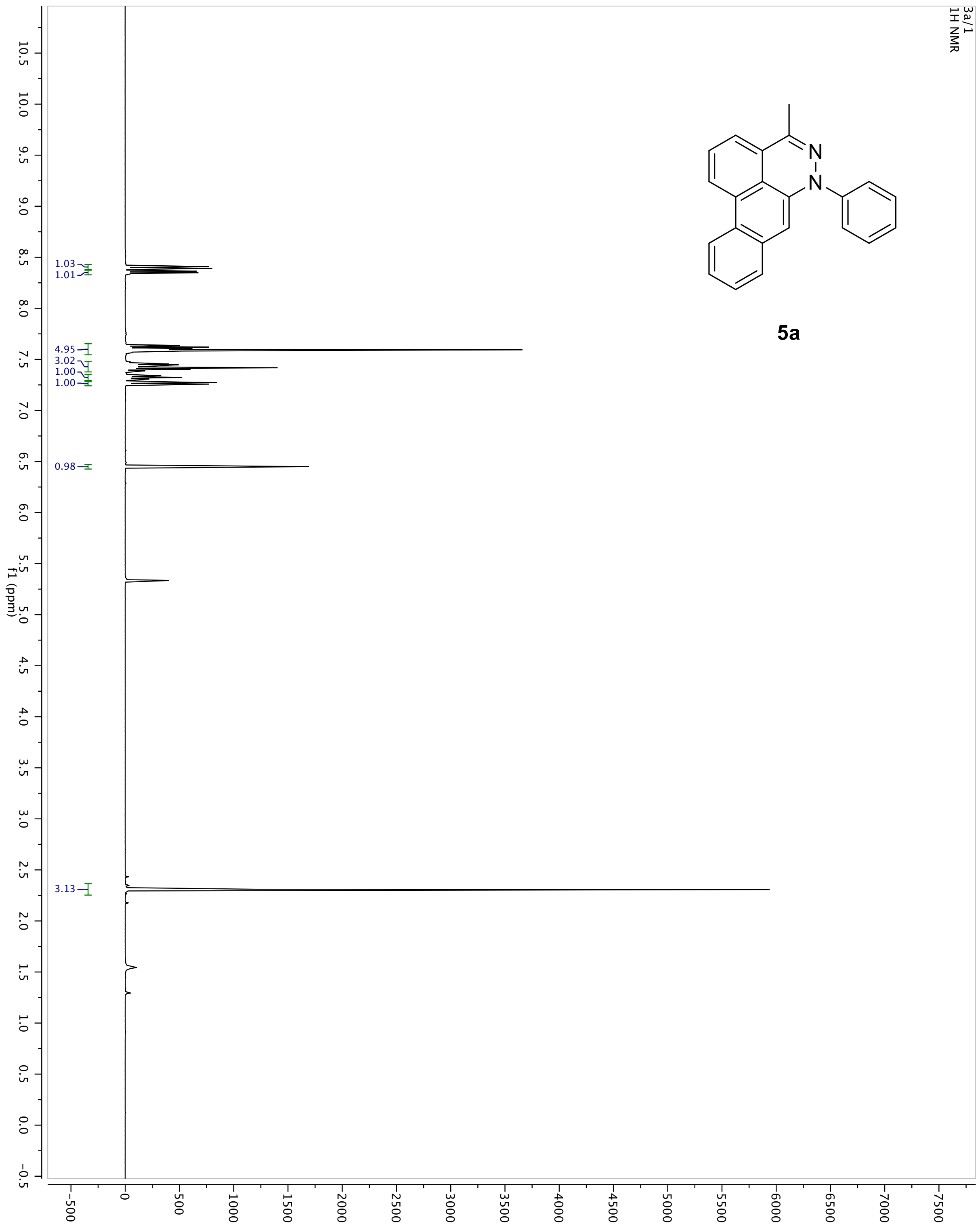

^{13}C NMR spectrum of **5a** in CD_2Cl_2 (125 MHz).

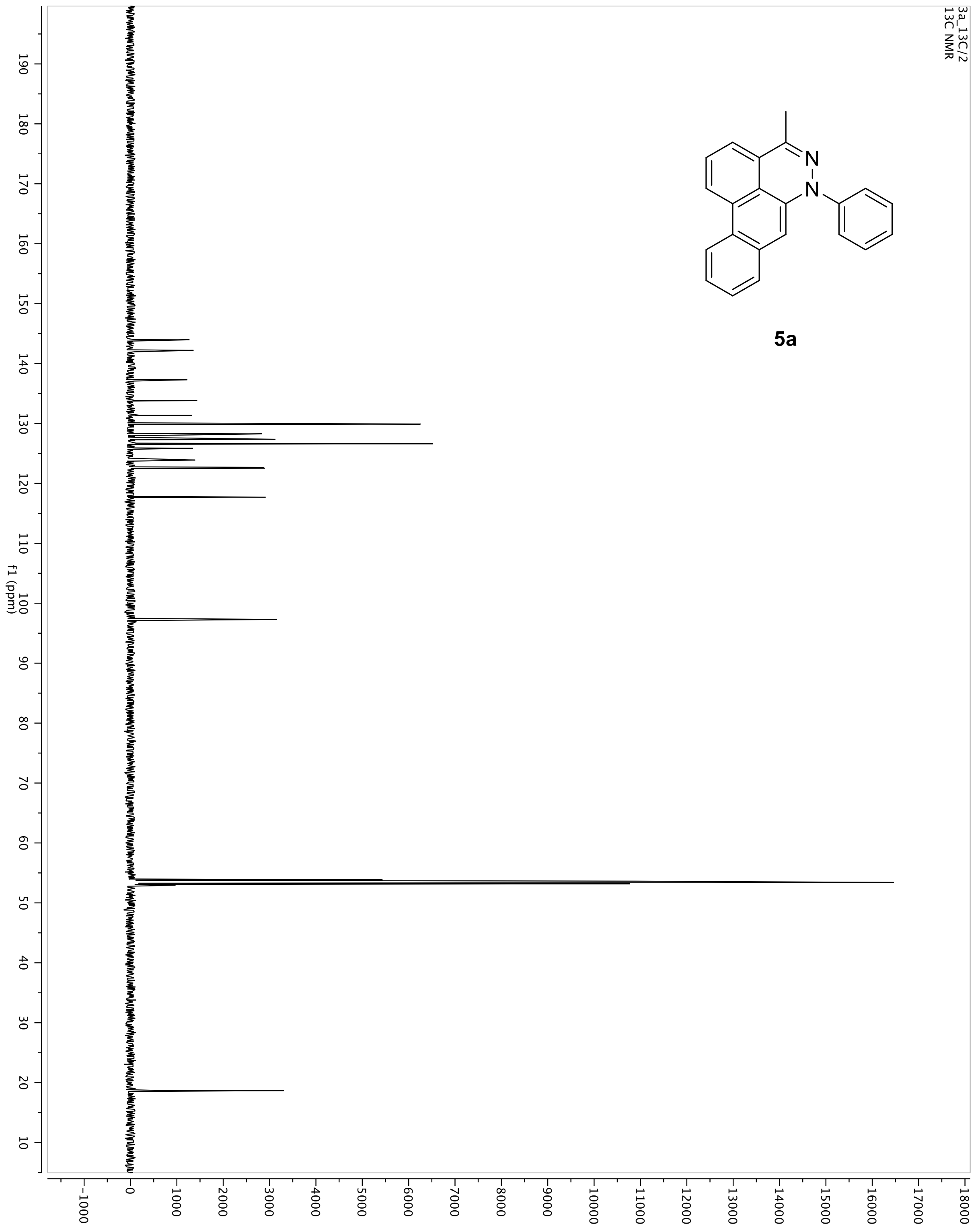

^{1}H NMR spectrum of **5b** in CD_2Cl_2 (500 MHz).

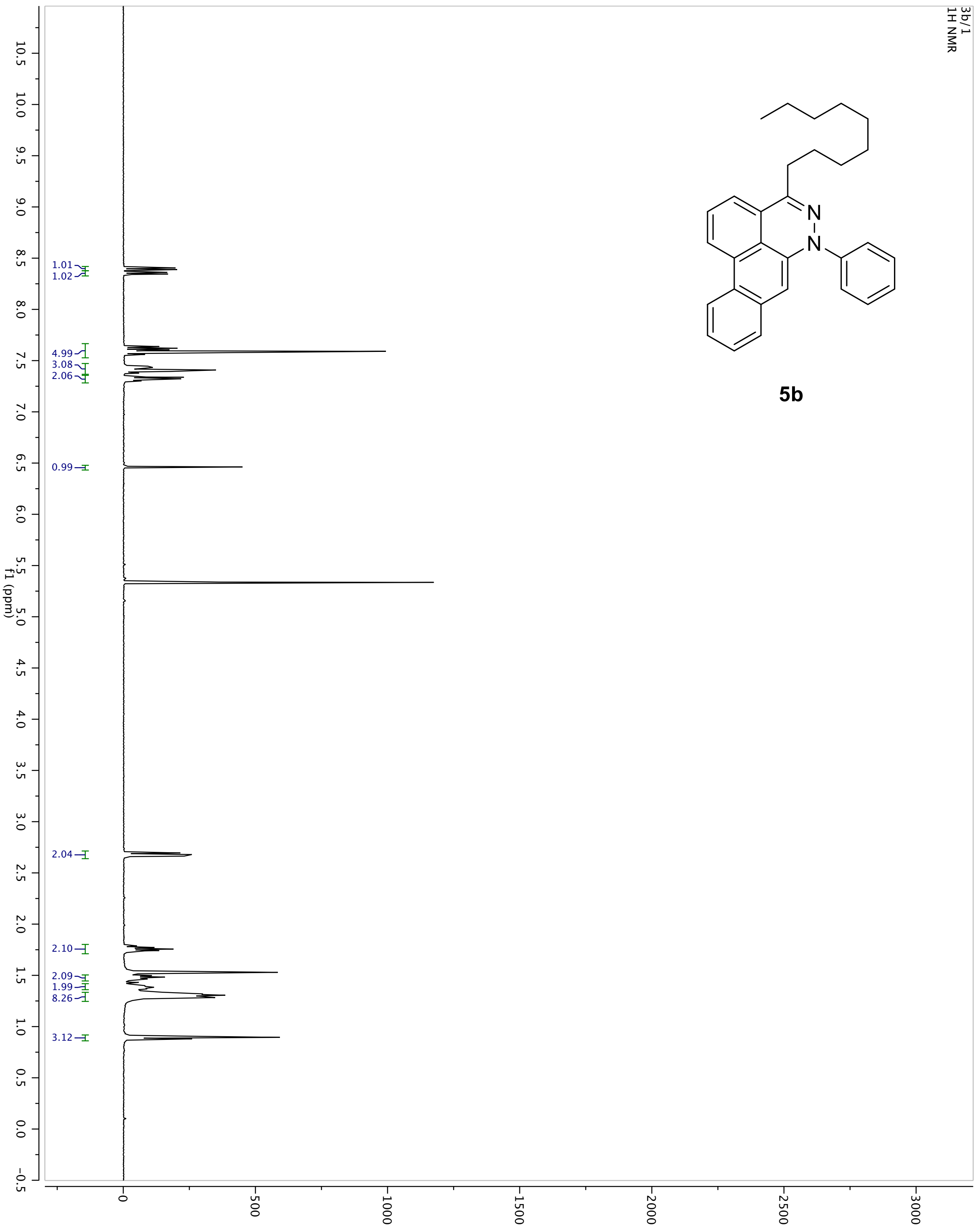

^{13}C NMR spectrum of **5b** in CD_2Cl_2 (125 MHz).

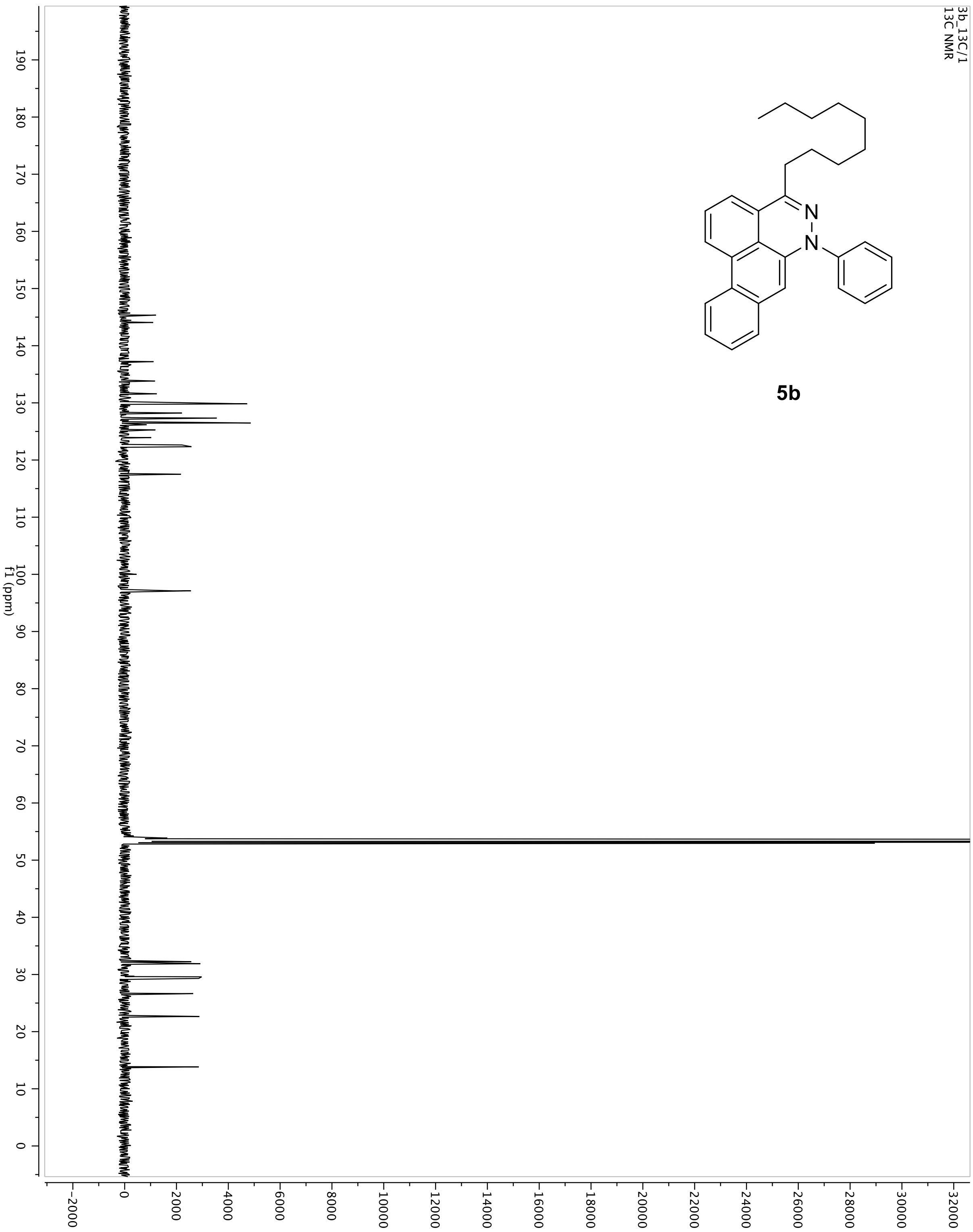

^{1}H NMR spectrum of **5c** in CD_2Cl_2 (500 MHz).

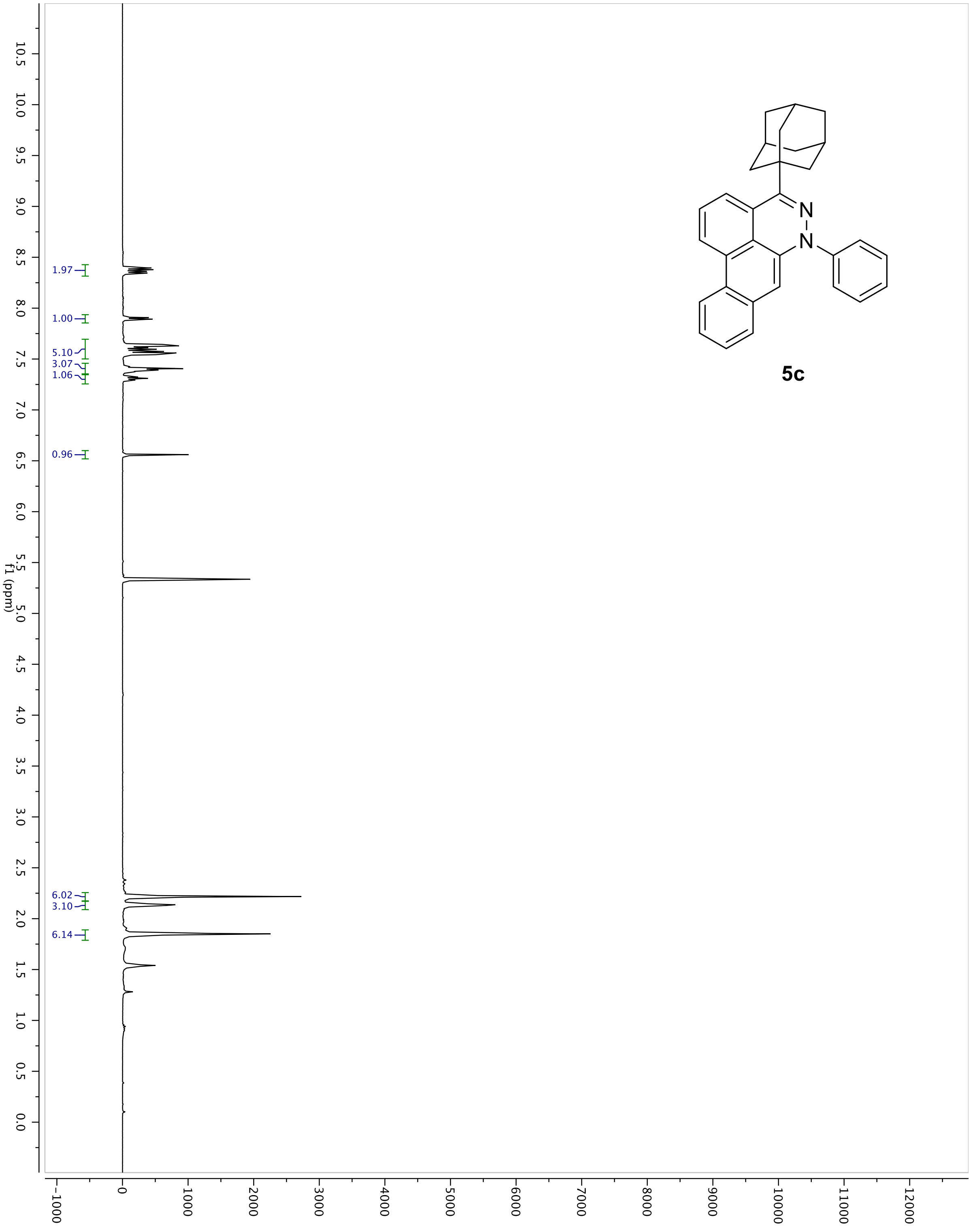

^{13}C NMR spectrum of **5c** in CD_2Cl_2 (125 MHz).

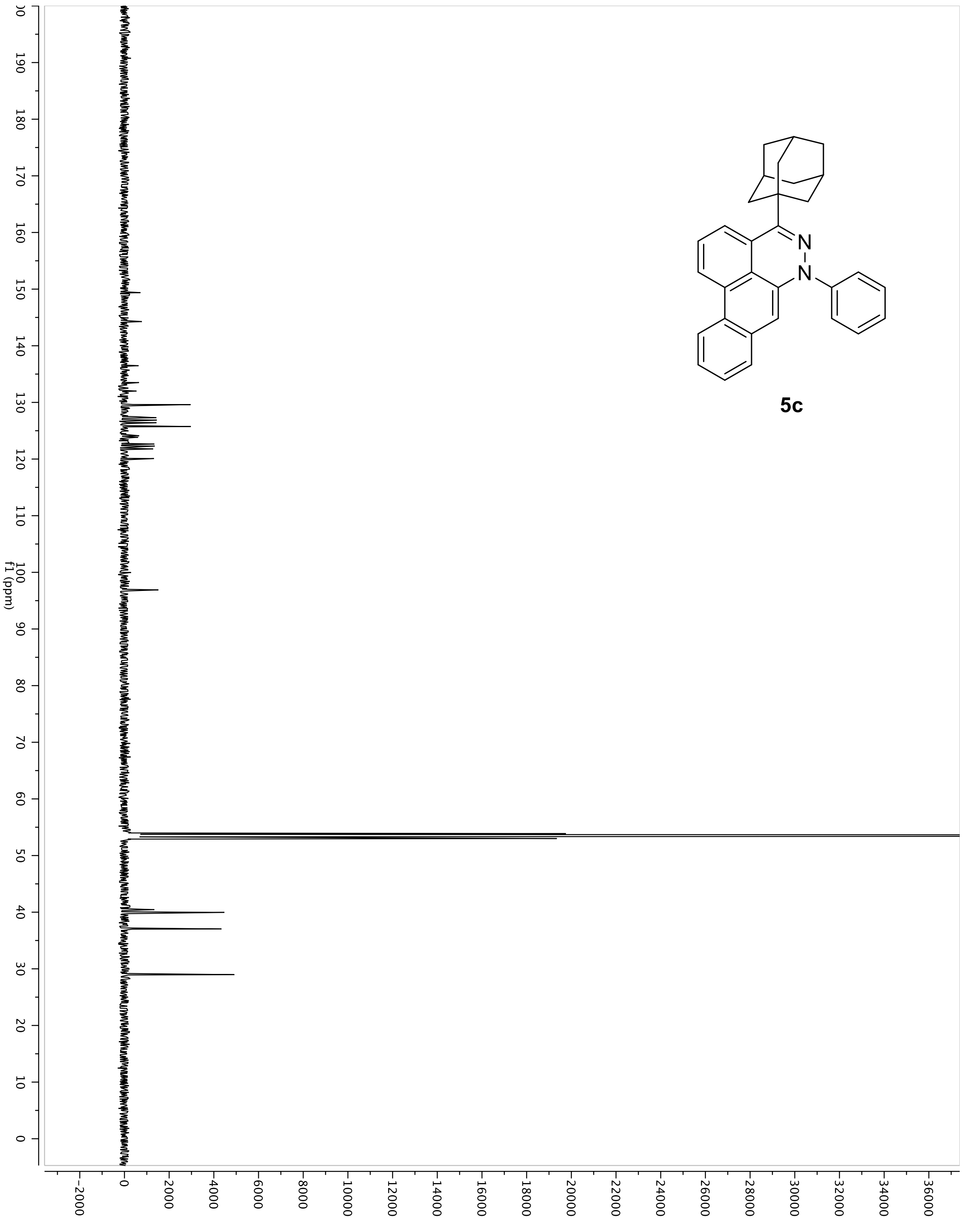

^{1}H NMR spectrum of **5d** in CD_2Cl_2 (500 MHz).

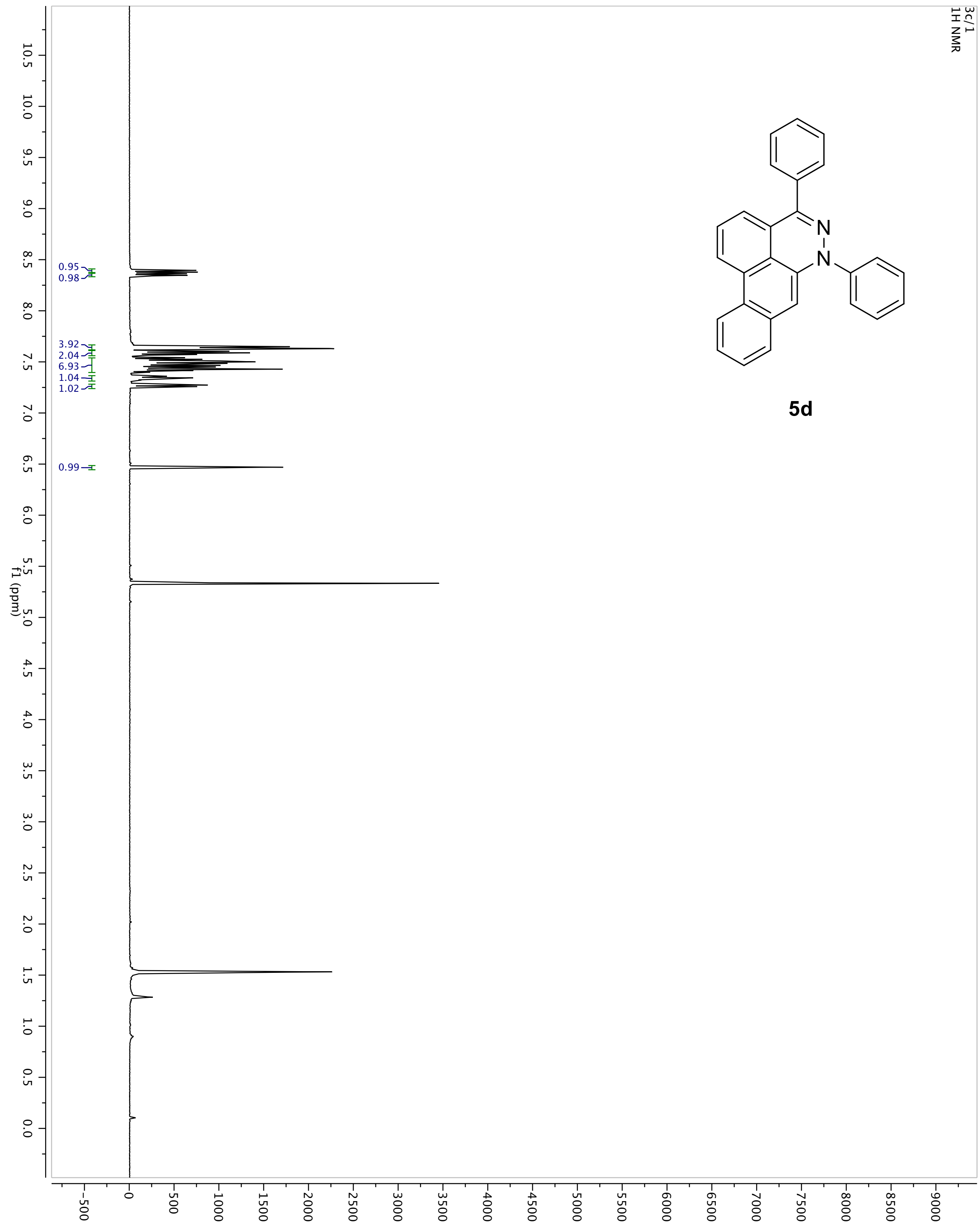

^{13}C NMR spectrum of **5d** in CD_2Cl_2 (125 MHz).

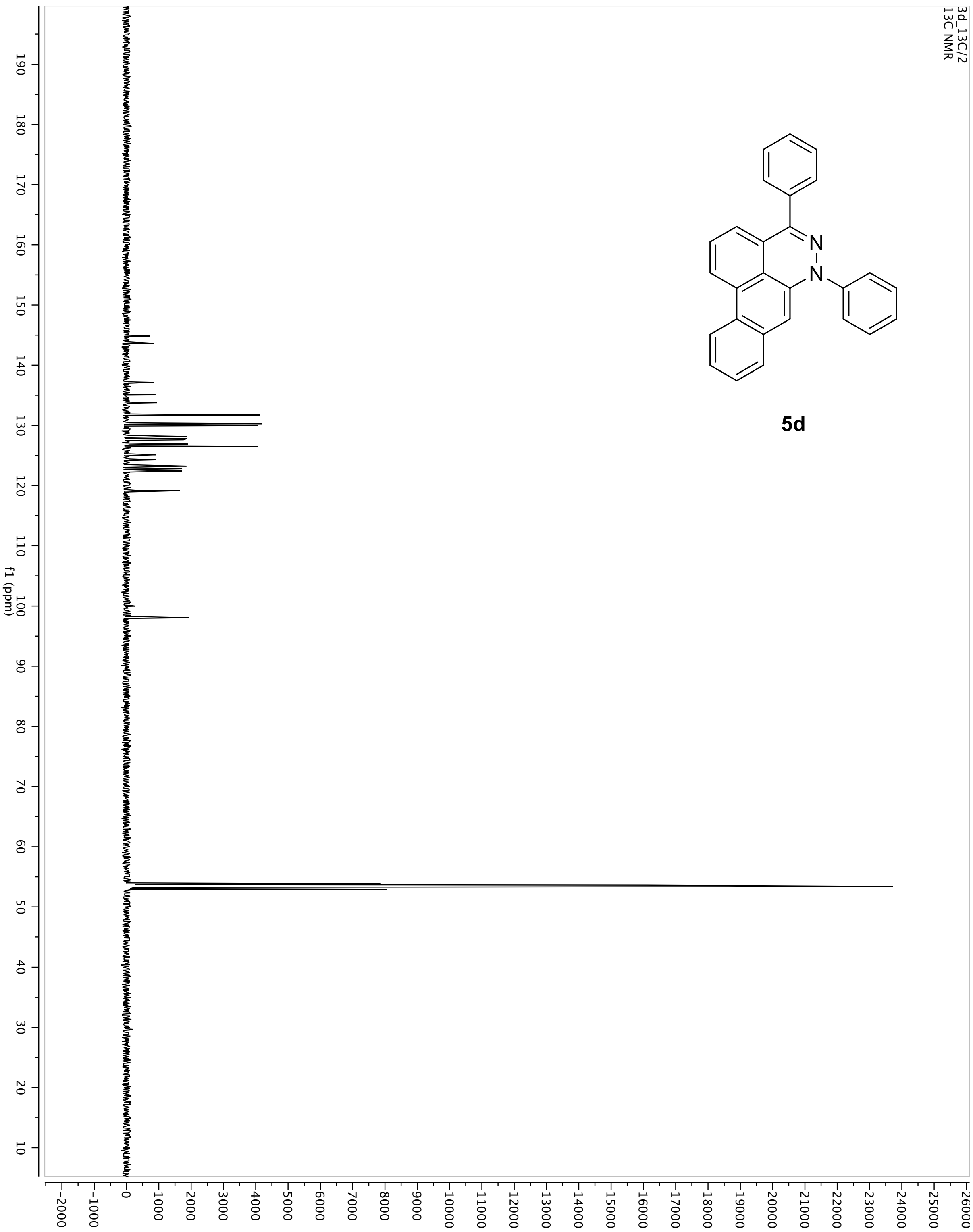

^{1}H NMR spectrum of **5e** in CD_2Cl_2-d_2 (500 MHz).

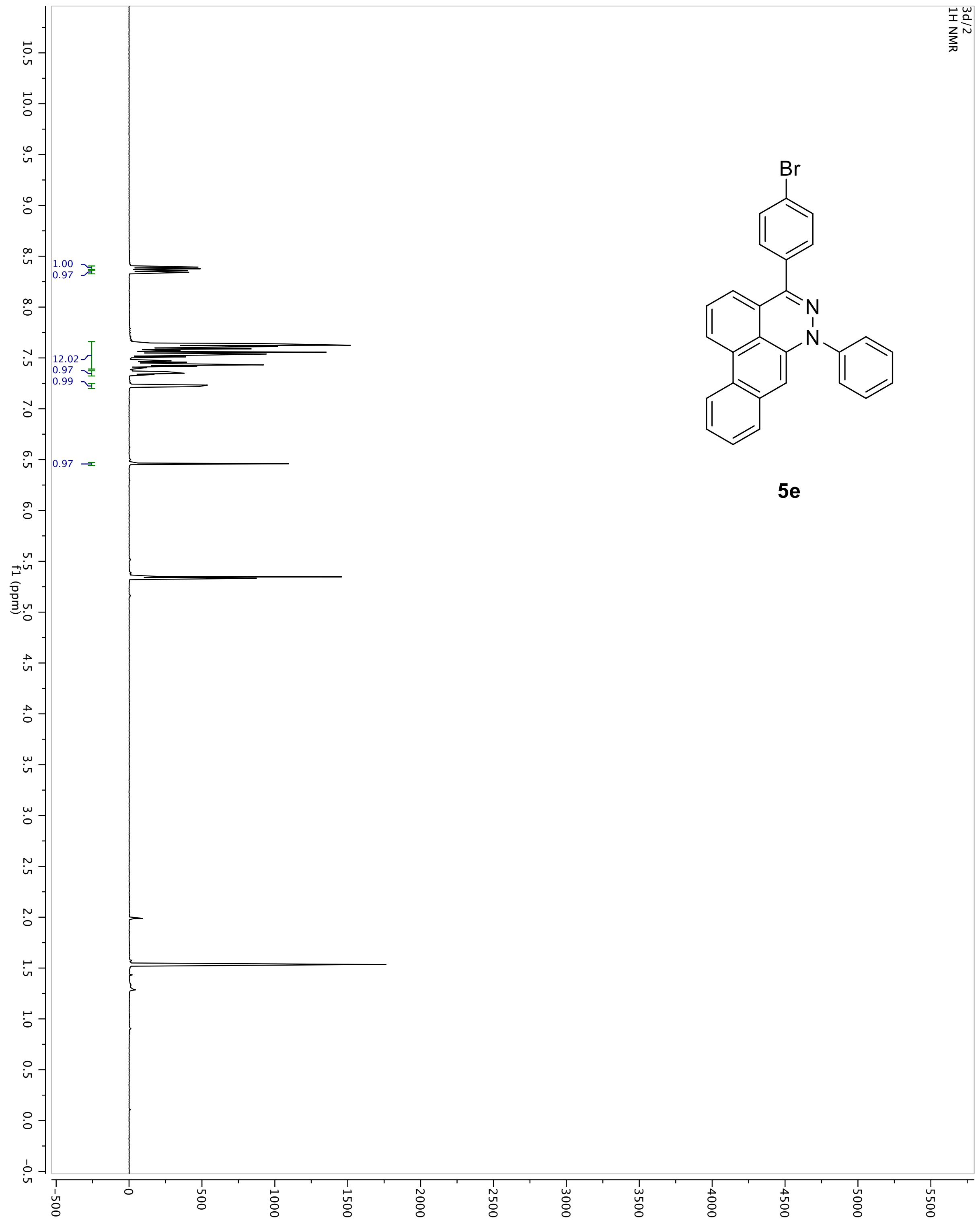

^{13}C NMR spectrum of 5e in CD_2Cl_2 (125 MHz).

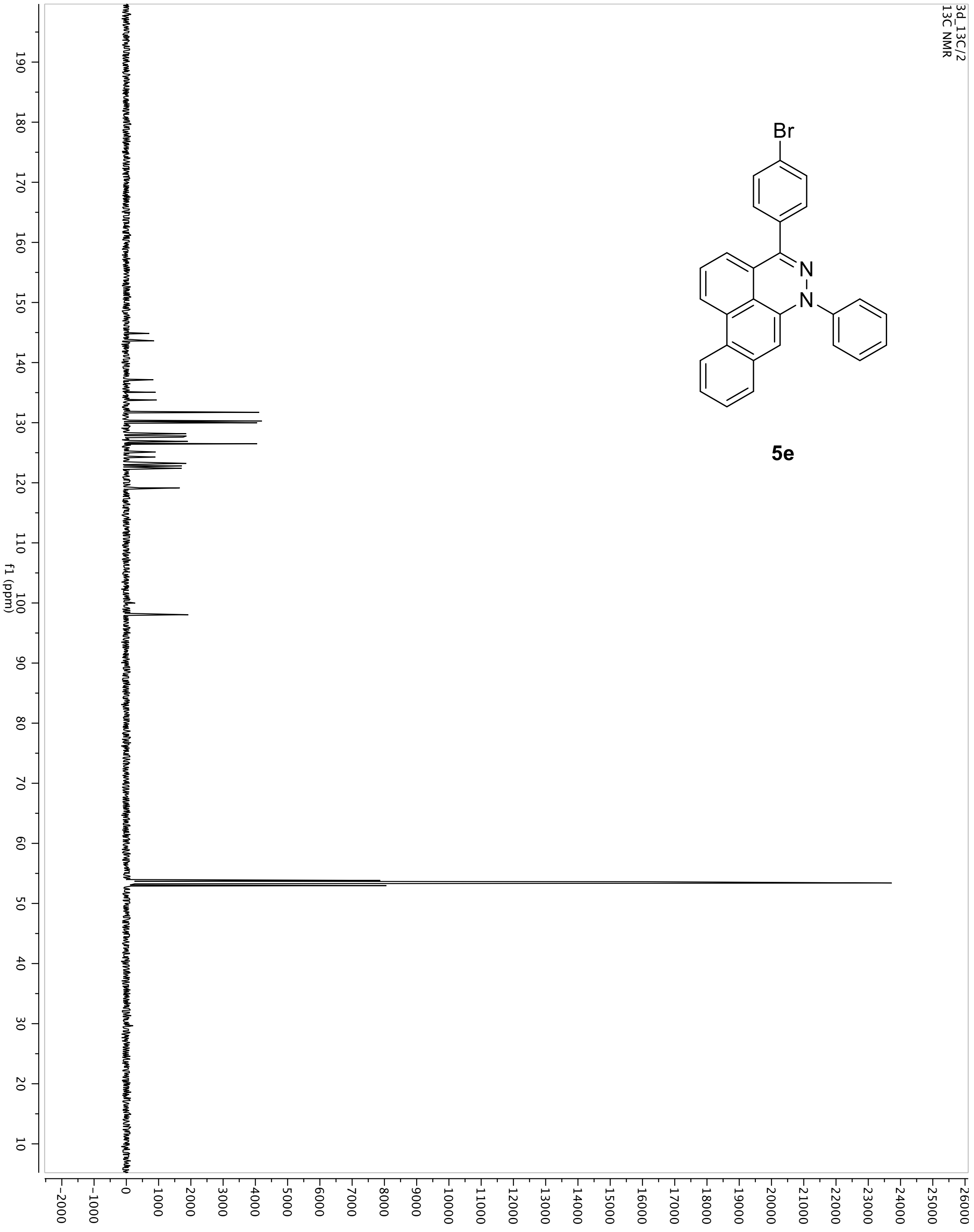

^{1}H NMR spectrum of **5f** in CD_2Cl_2 (500 MHz).

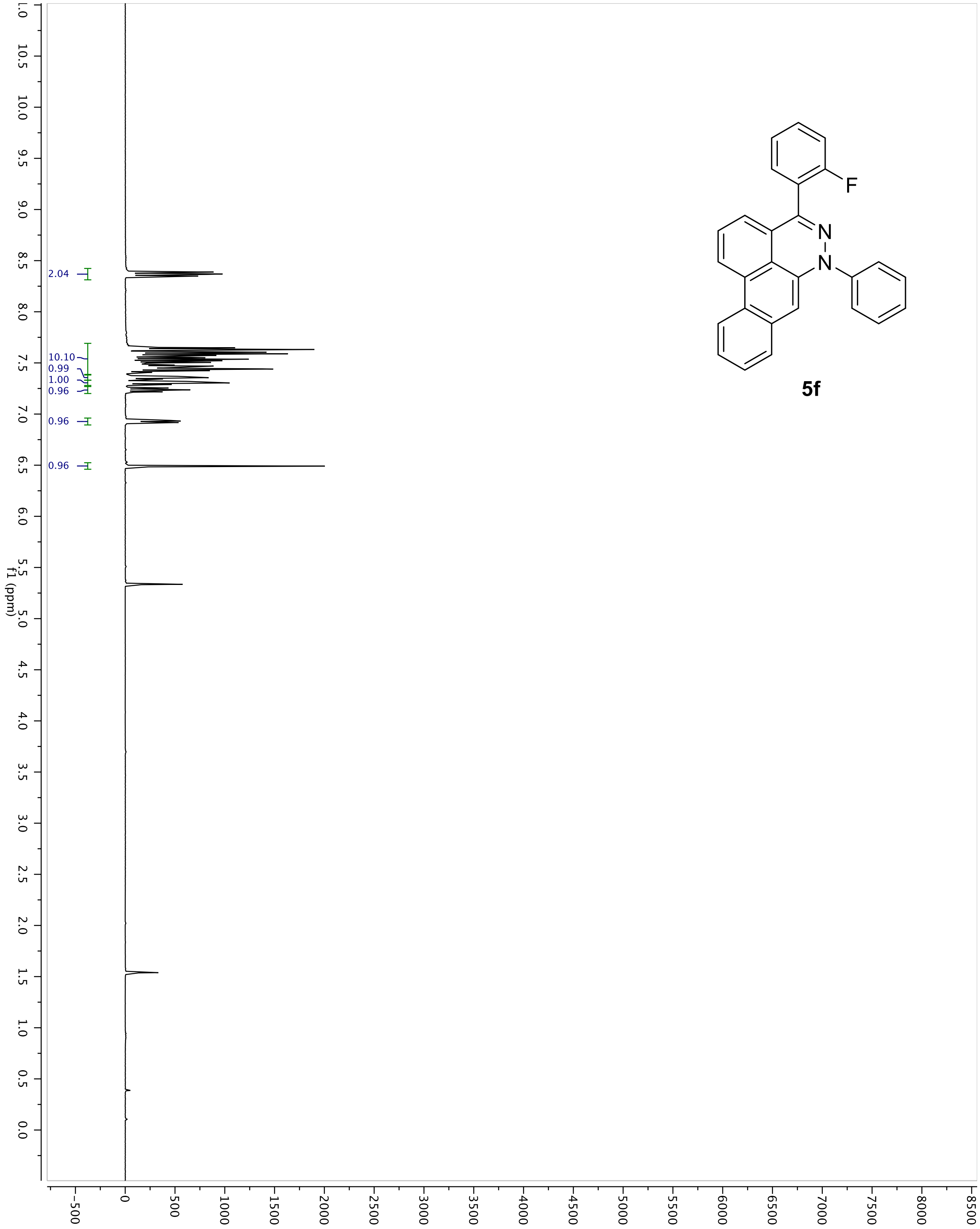

^{13}C NMR spectrum of **5f** in CD_2Cl_2 (125 MHz).

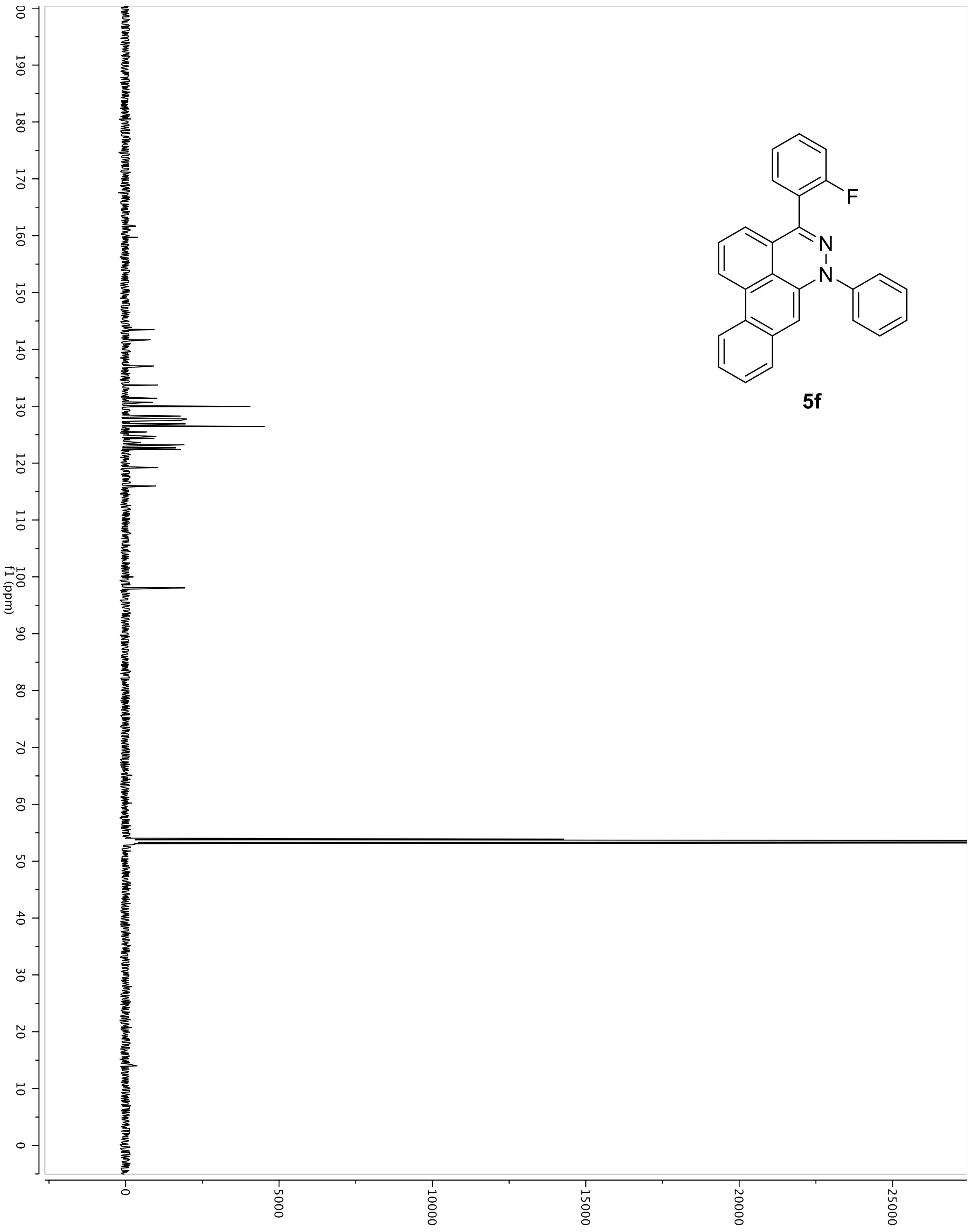

^{19}F NMR spectrum of **5f** in CD_2Cl_2 (338 MHz).

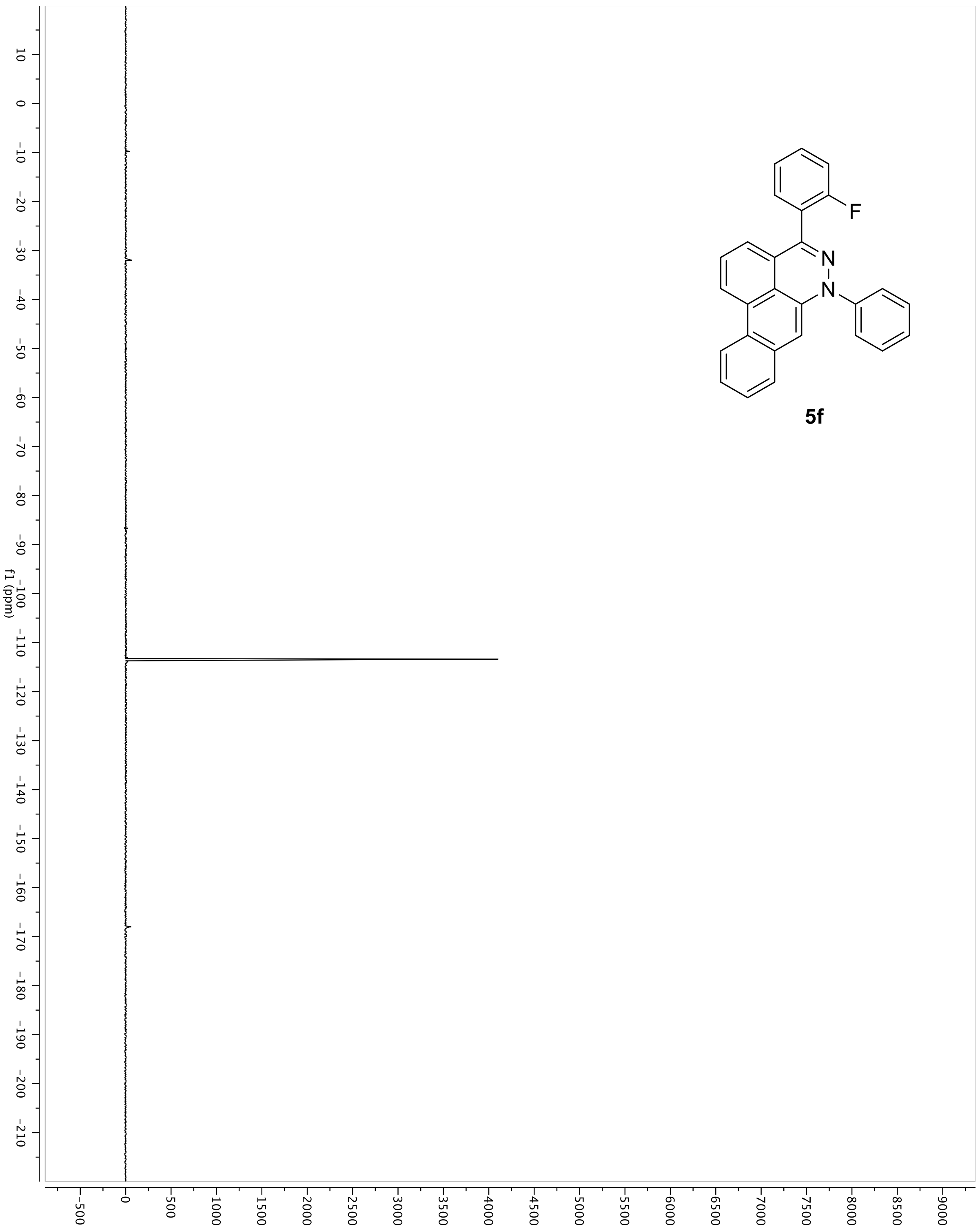

^{1}H NMR spectrum of **5g** in CD_2Cl_2 (500 MHz).

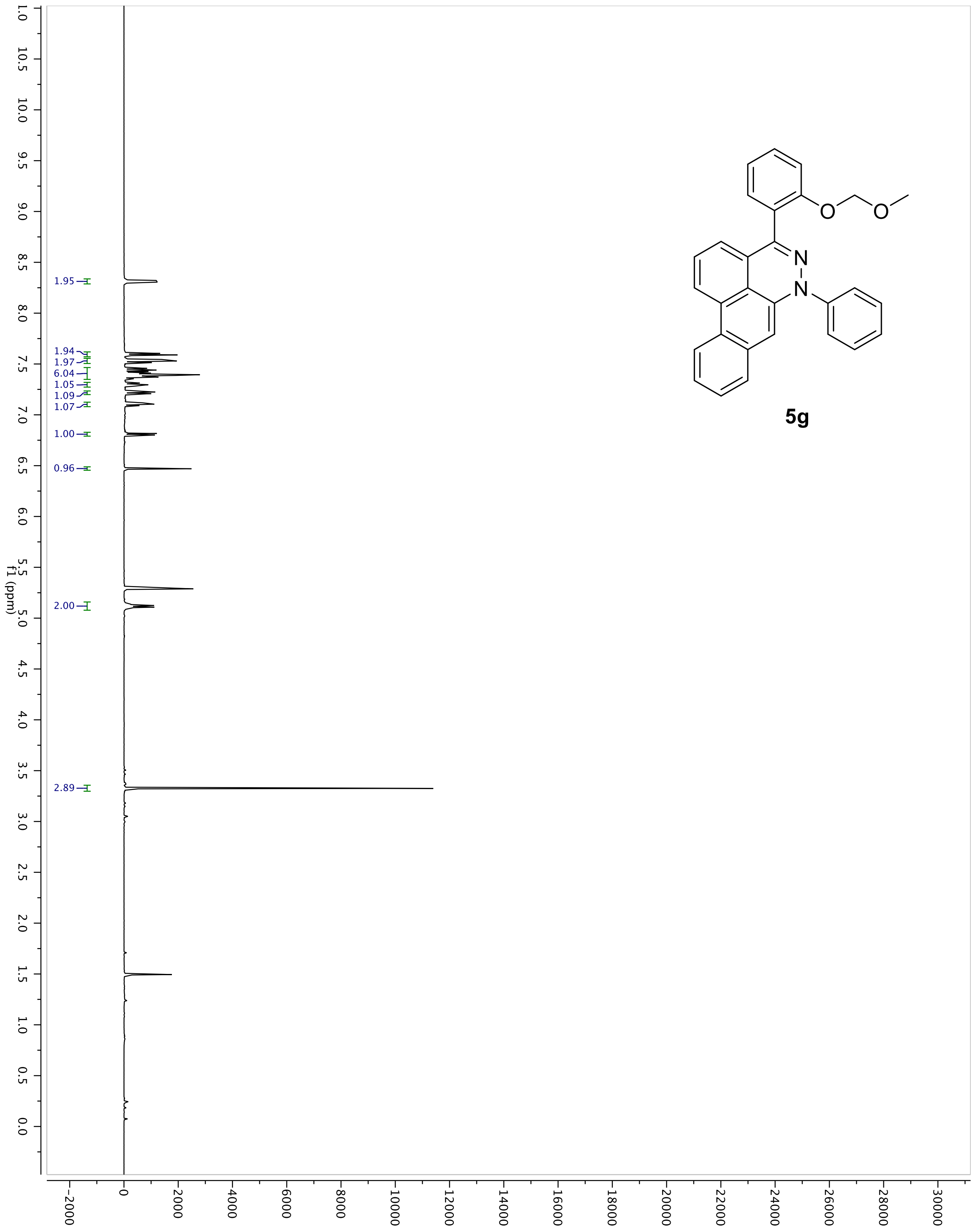

^{13}C NMR spectrum of **5g** in CD_2Cl_2 (125 MHz).

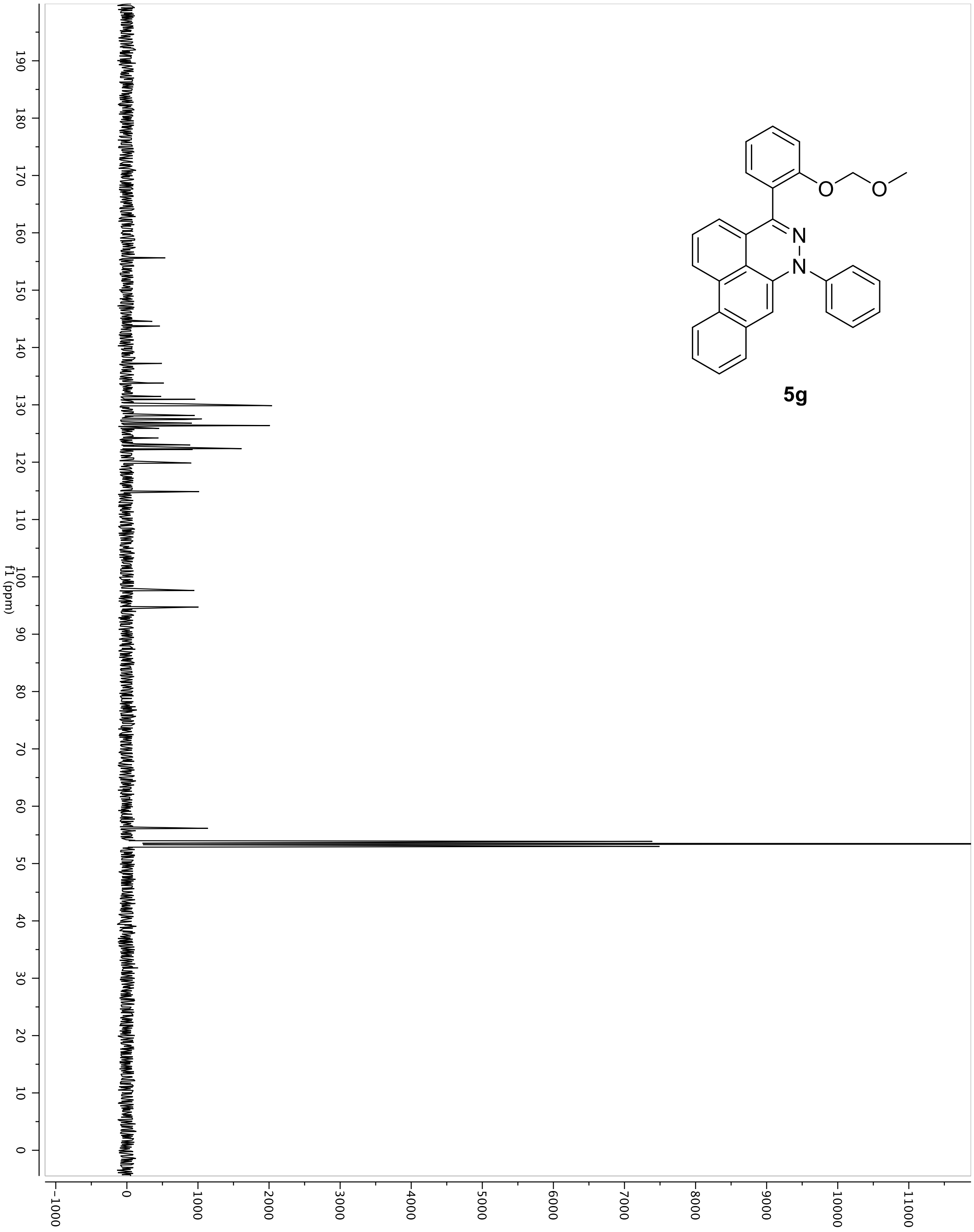

^{1}H NMR spectrum of **5h** in CD_2Cl_2 (500 MHz).

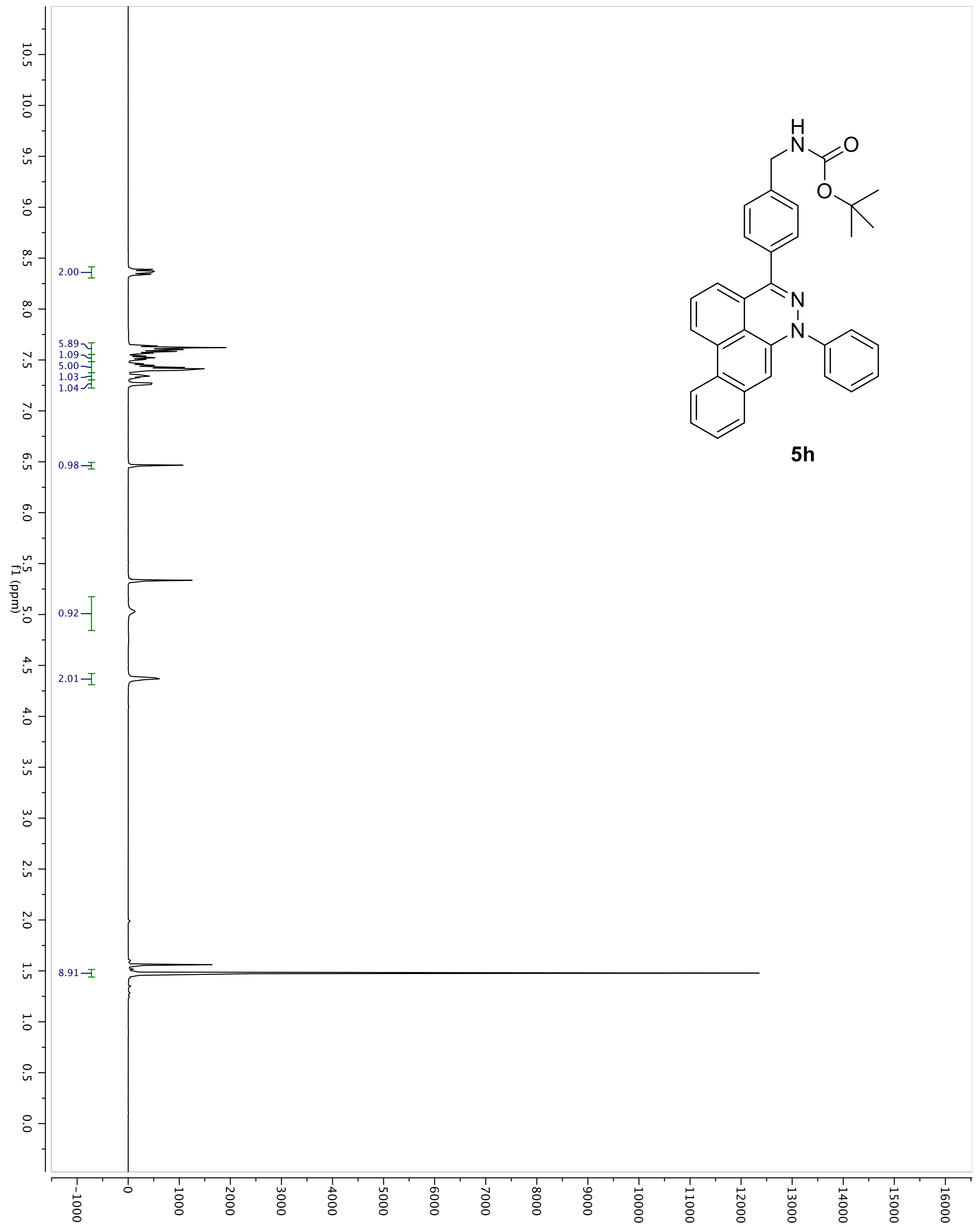

^{13}C NMR spectrum of **5h** in CD_2Cl_2 (125 MHz).

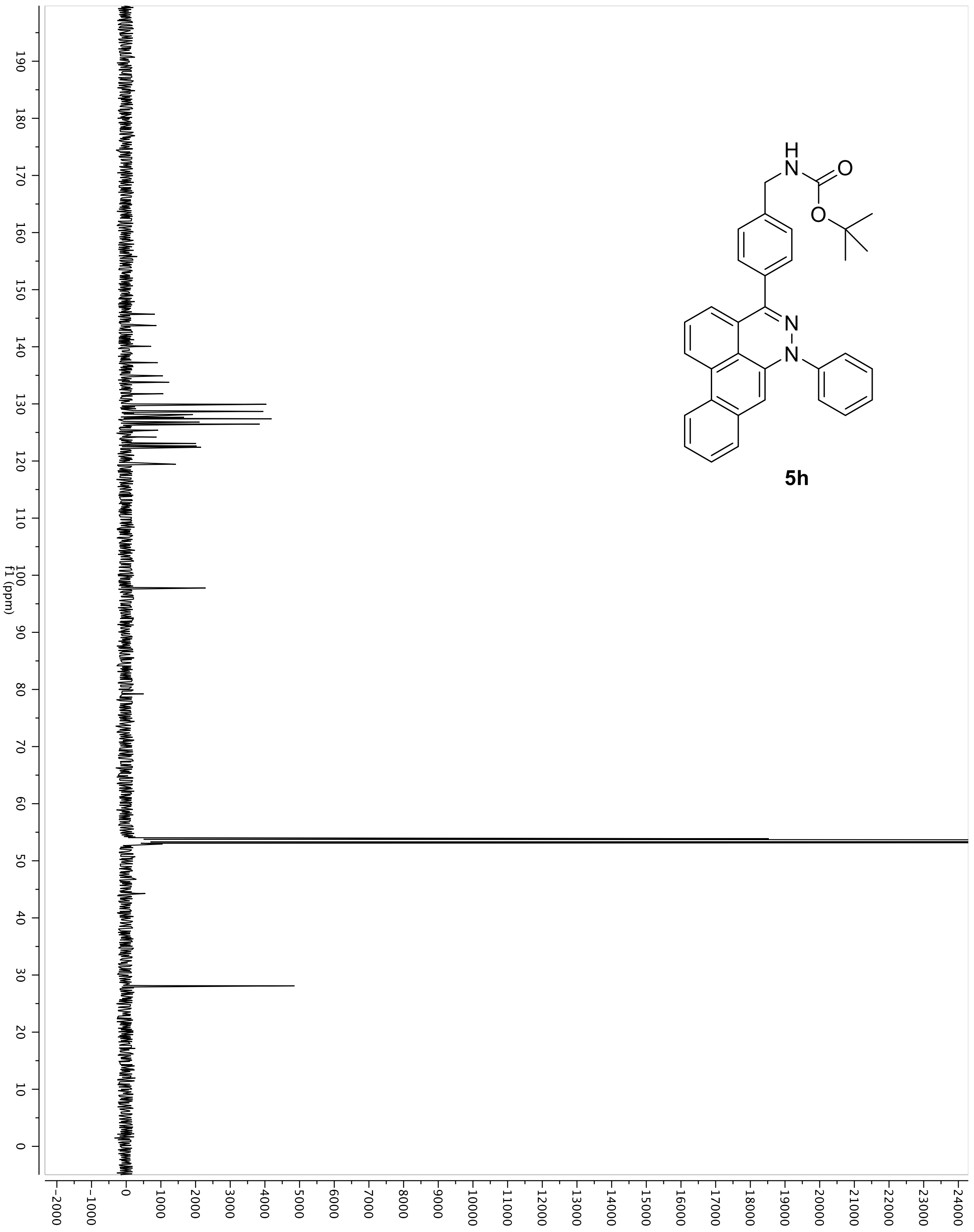

^{1}H NMR spectrum of **5i** in CD_2Cl_2 (500 MHz).

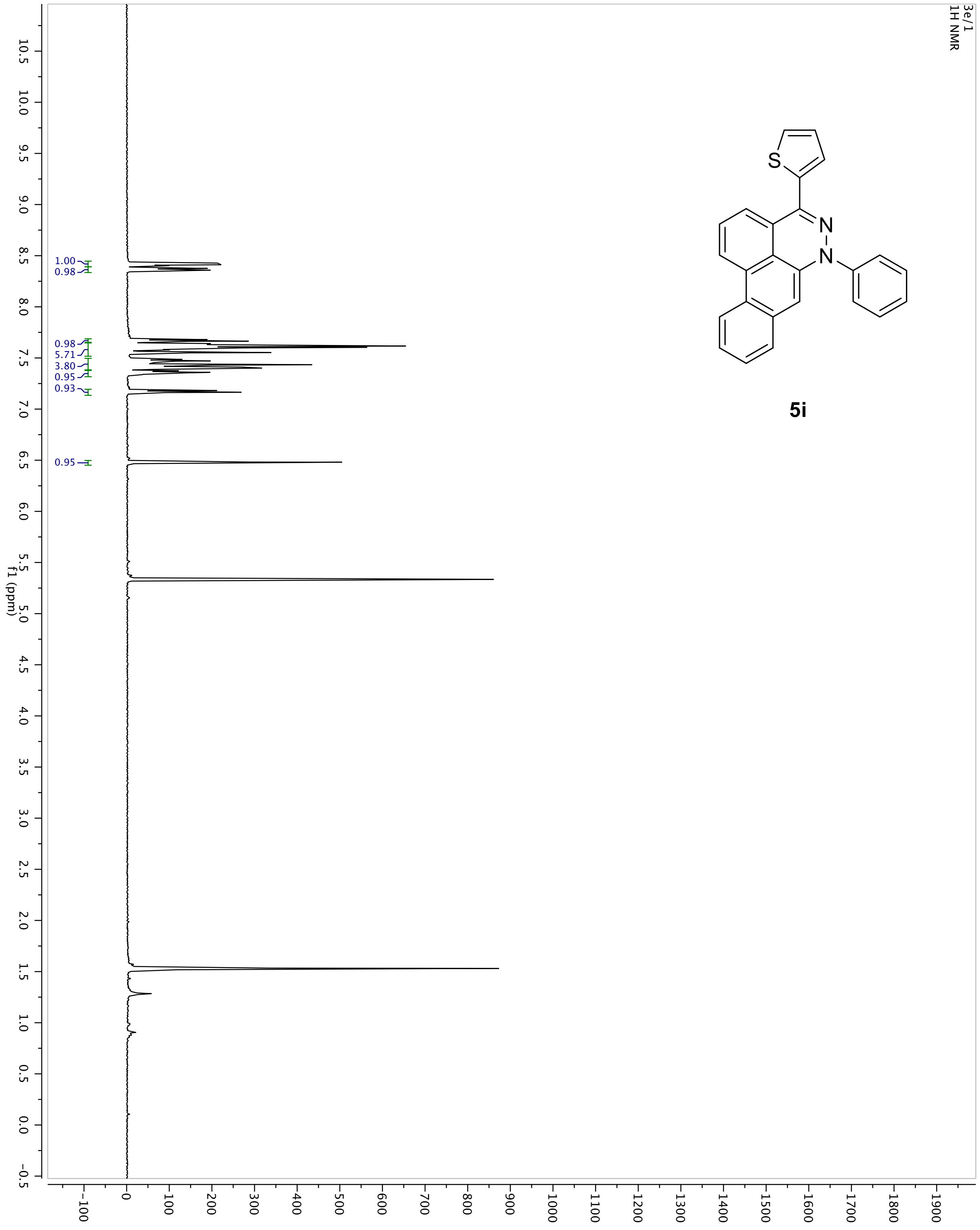

^{13}C NMR spectrum of **5i** in CD_2Cl_2 (125 MHz).

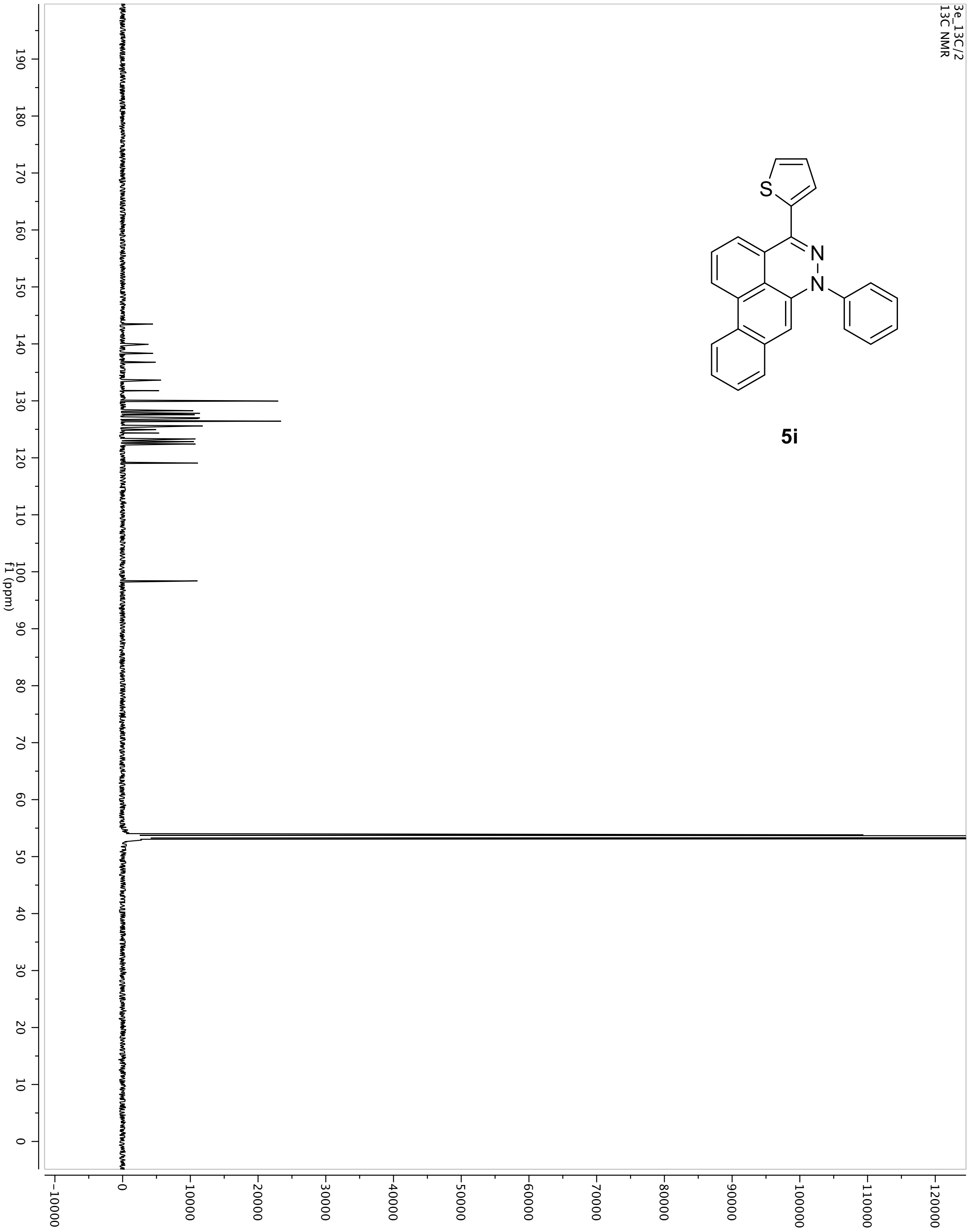

^{1}H NMR spectrum of **5j** in CD_2Cl_2 (500 MHz).

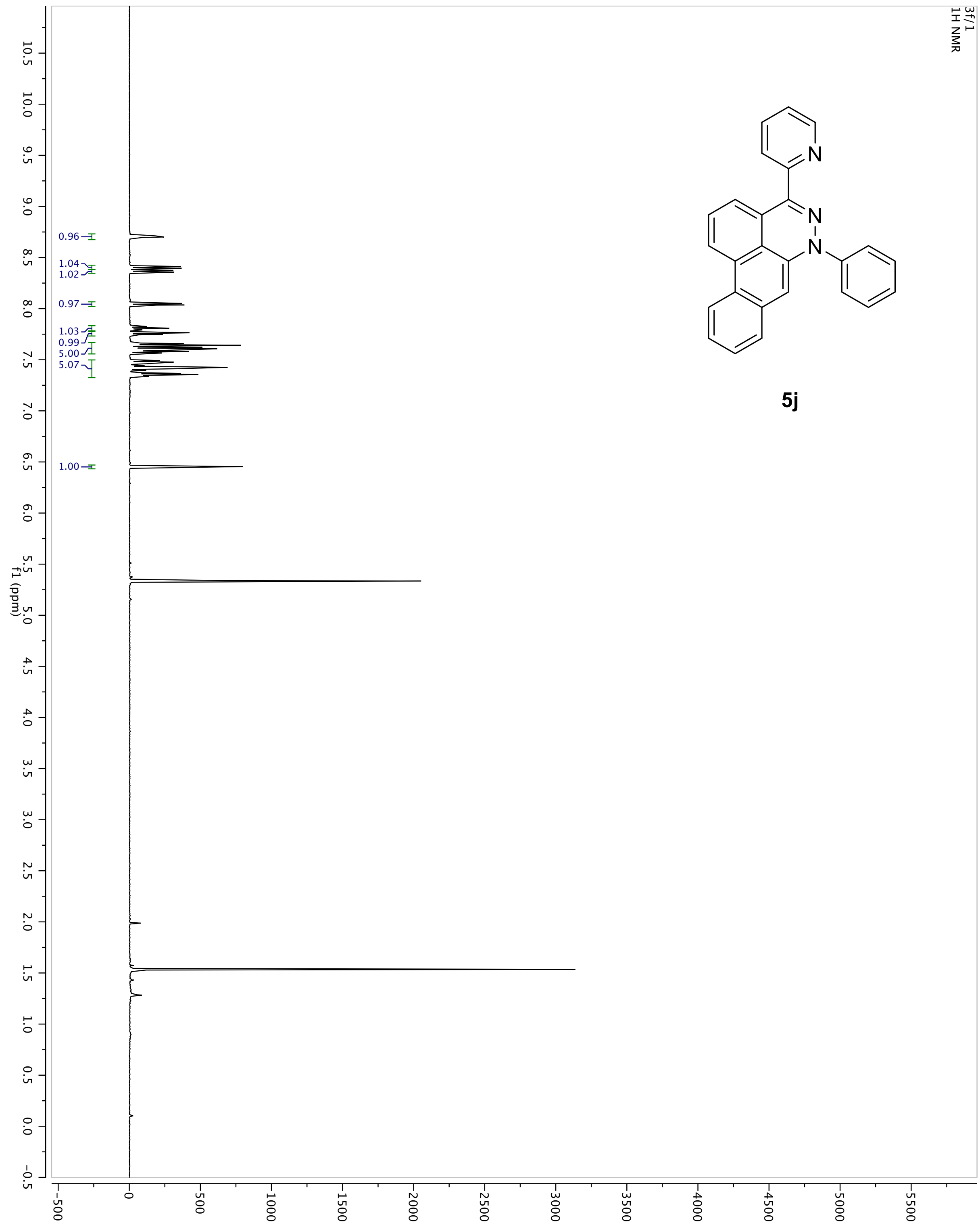

^{13}C NMR spectrum of **5j** in CD_2Cl_2 (125 MHz).

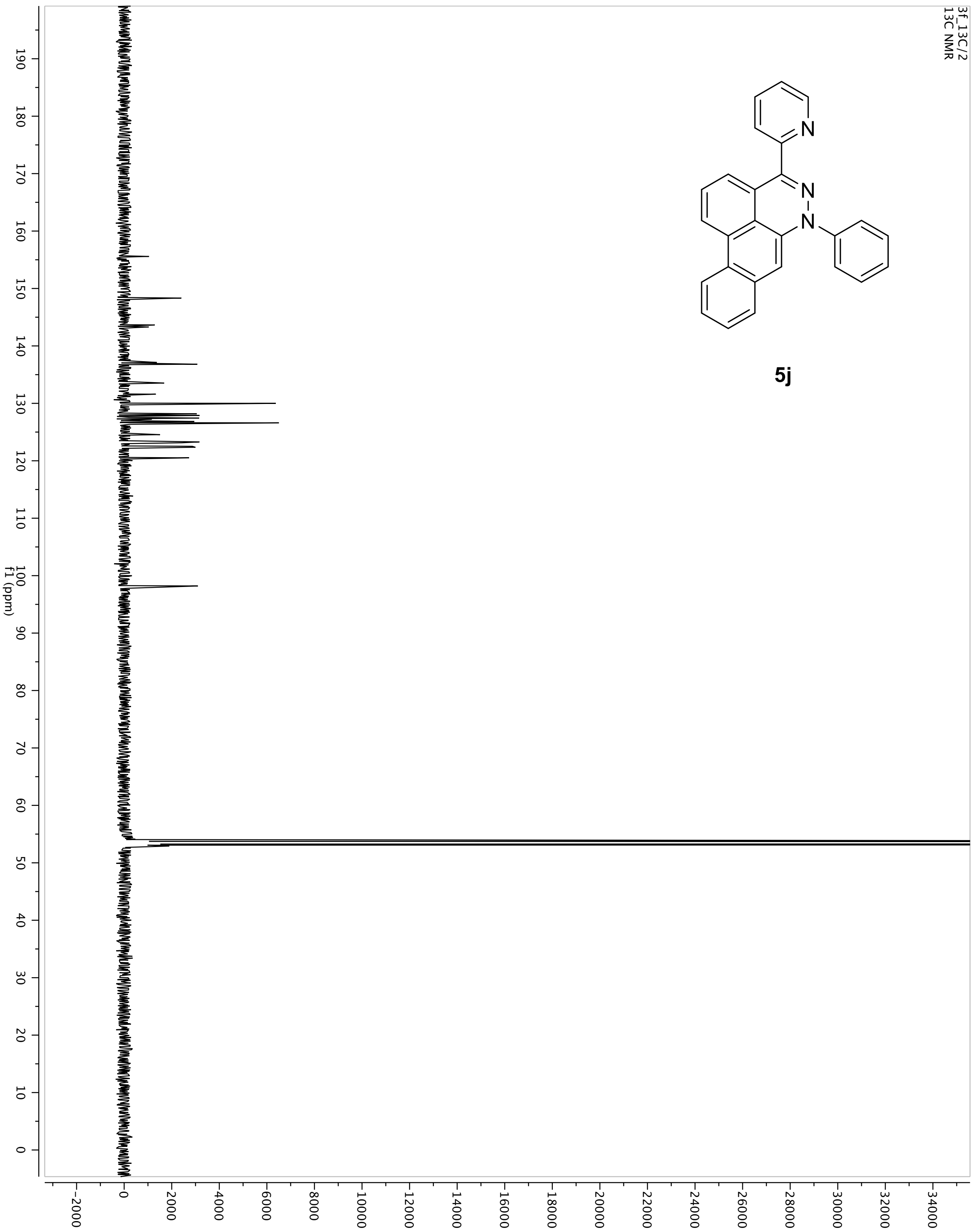

^{1}H NMR spectrum of **5k** in CD_2Cl_2 (500 MHz).

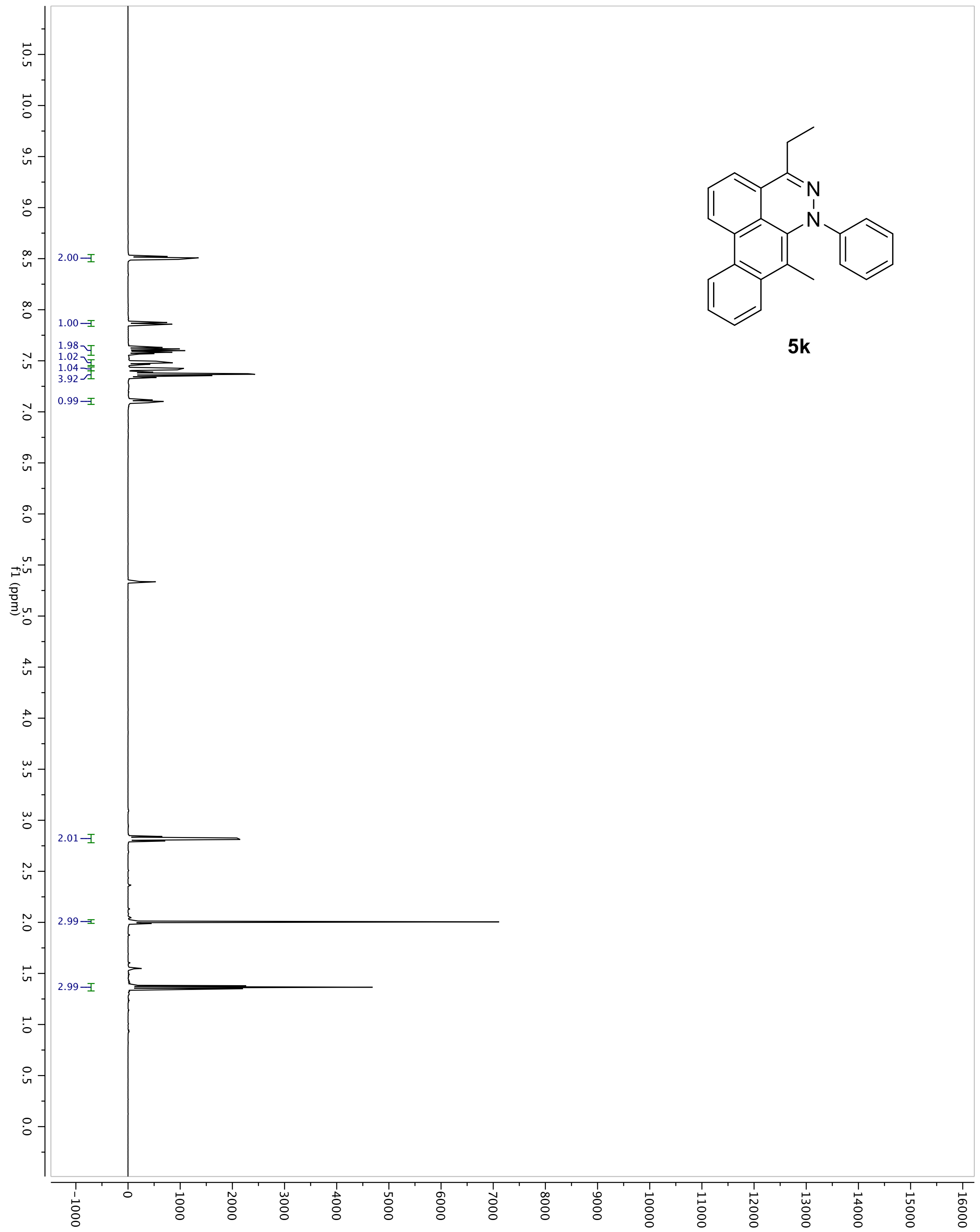

^{13}C NMR spectrum of **5k** in CD_2Cl_2 (125 MHz).

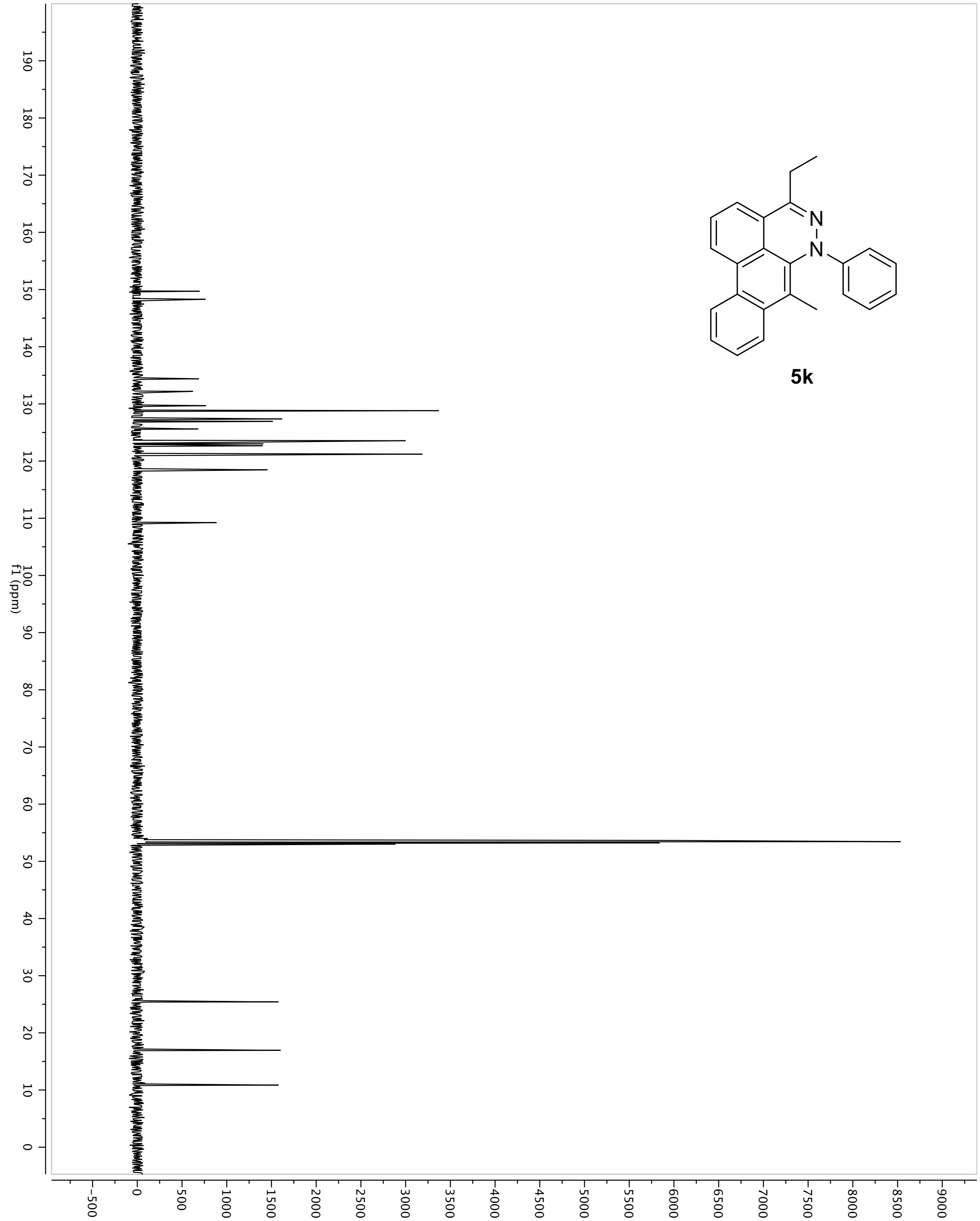

^{1}H NMR spectrum of **5l** in CD_2Cl_2 (500 MHz).

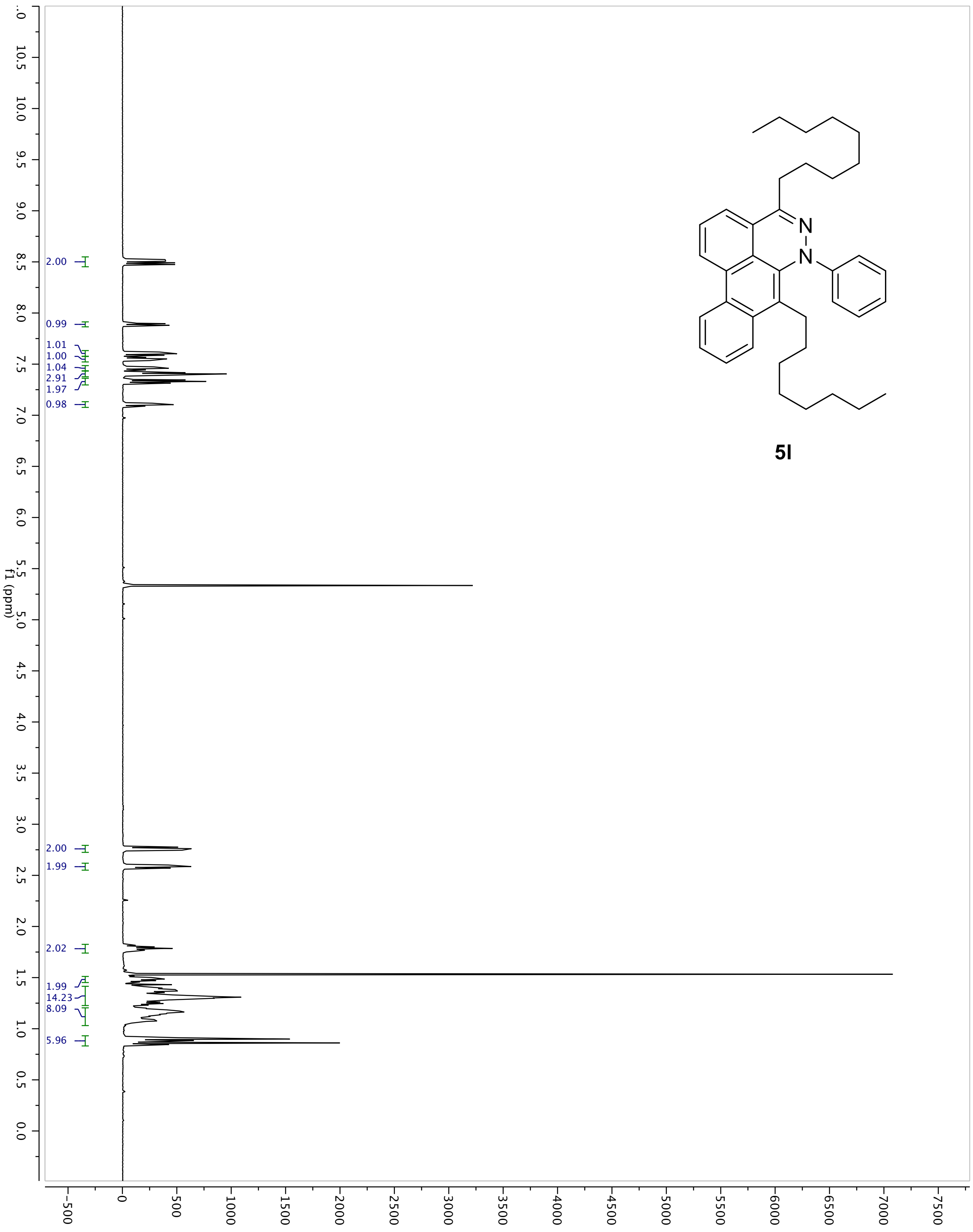

^{13}C NMR spectrum of **5l** in CD_2Cl_2 (125 MHz).

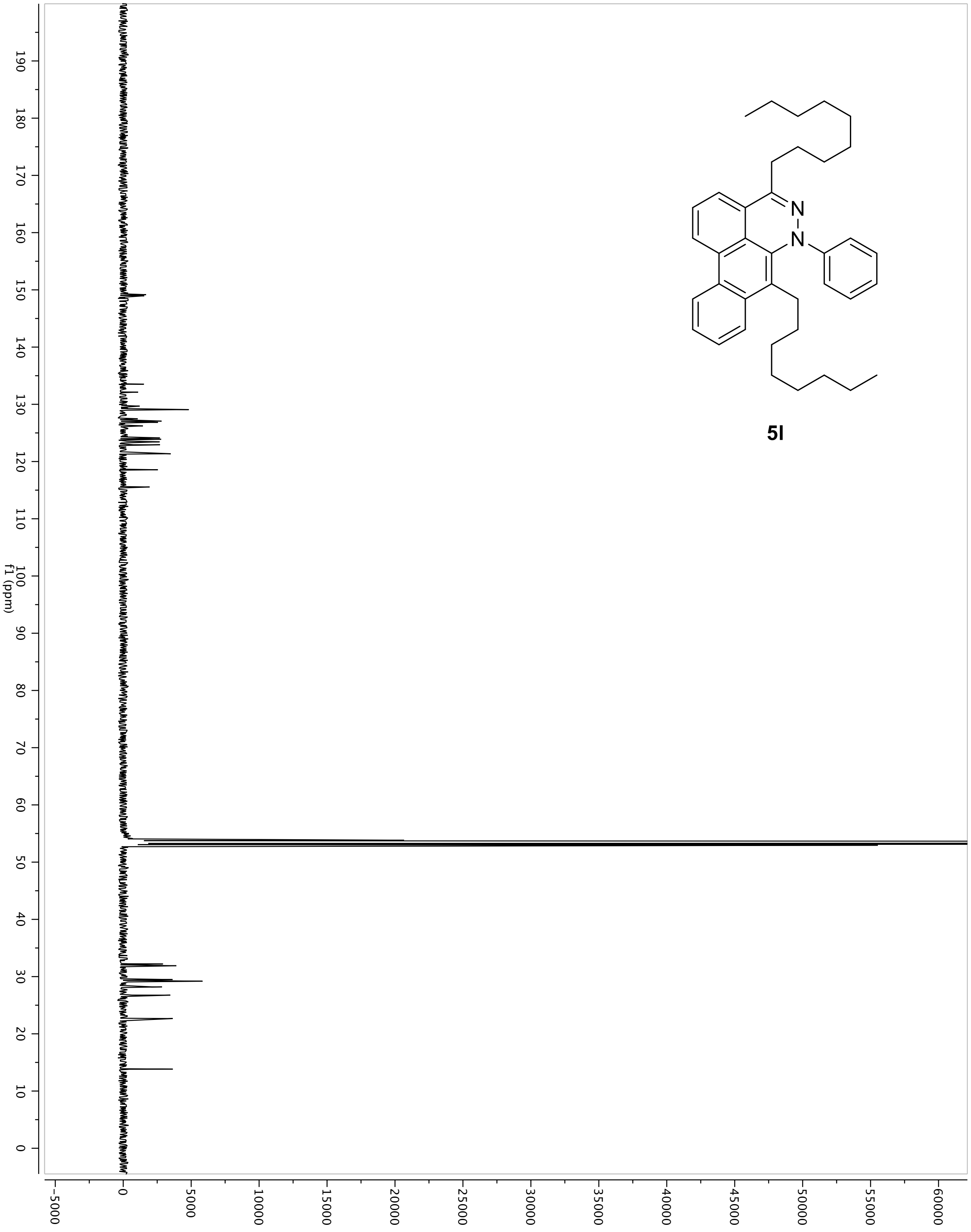

^{1}H NMR spectrum of **5m** in CD_2Cl_2 (500 MHz).

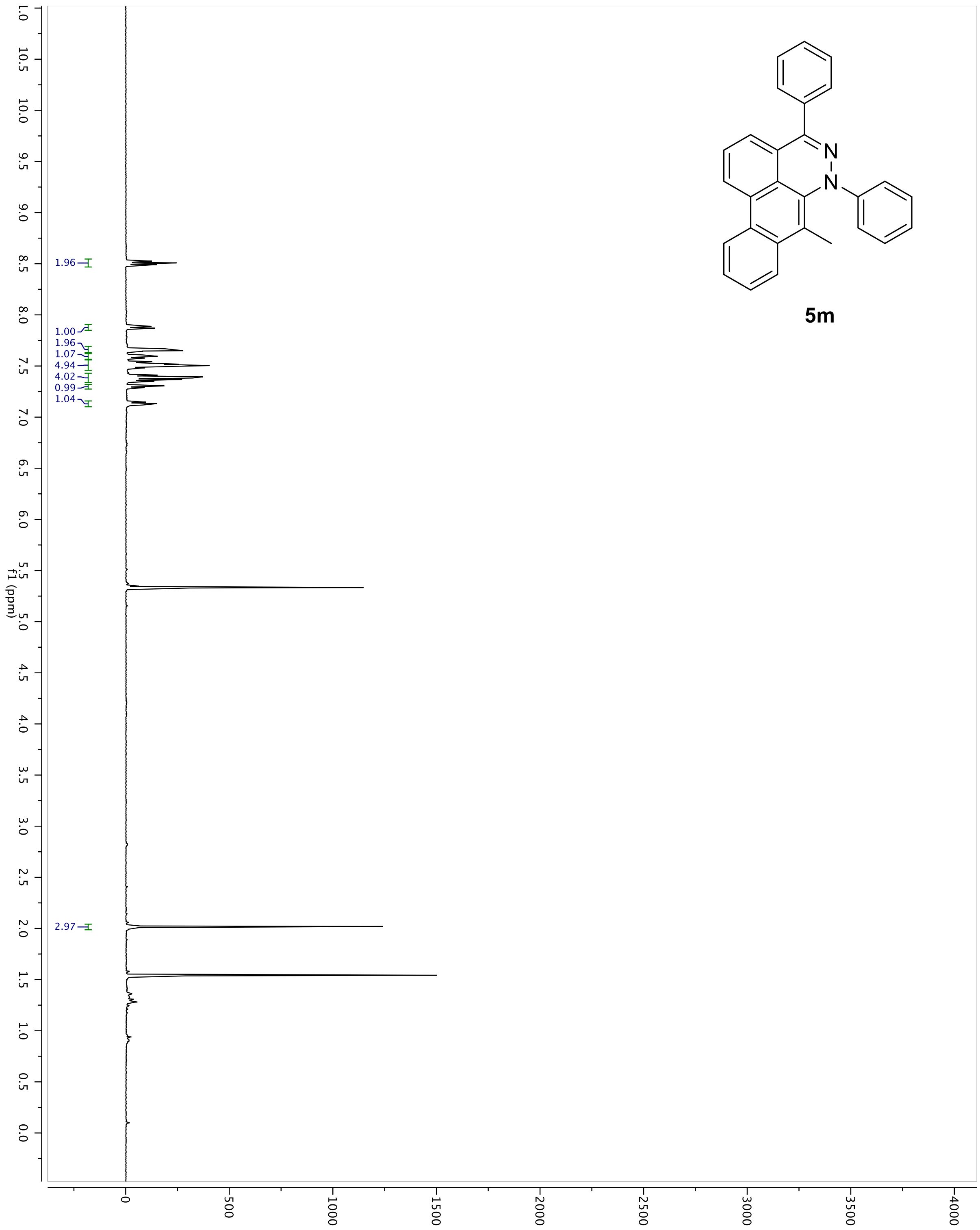

^{13}C NMR spectrum of **5m** in CD_2Cl_2 (125 MHz).

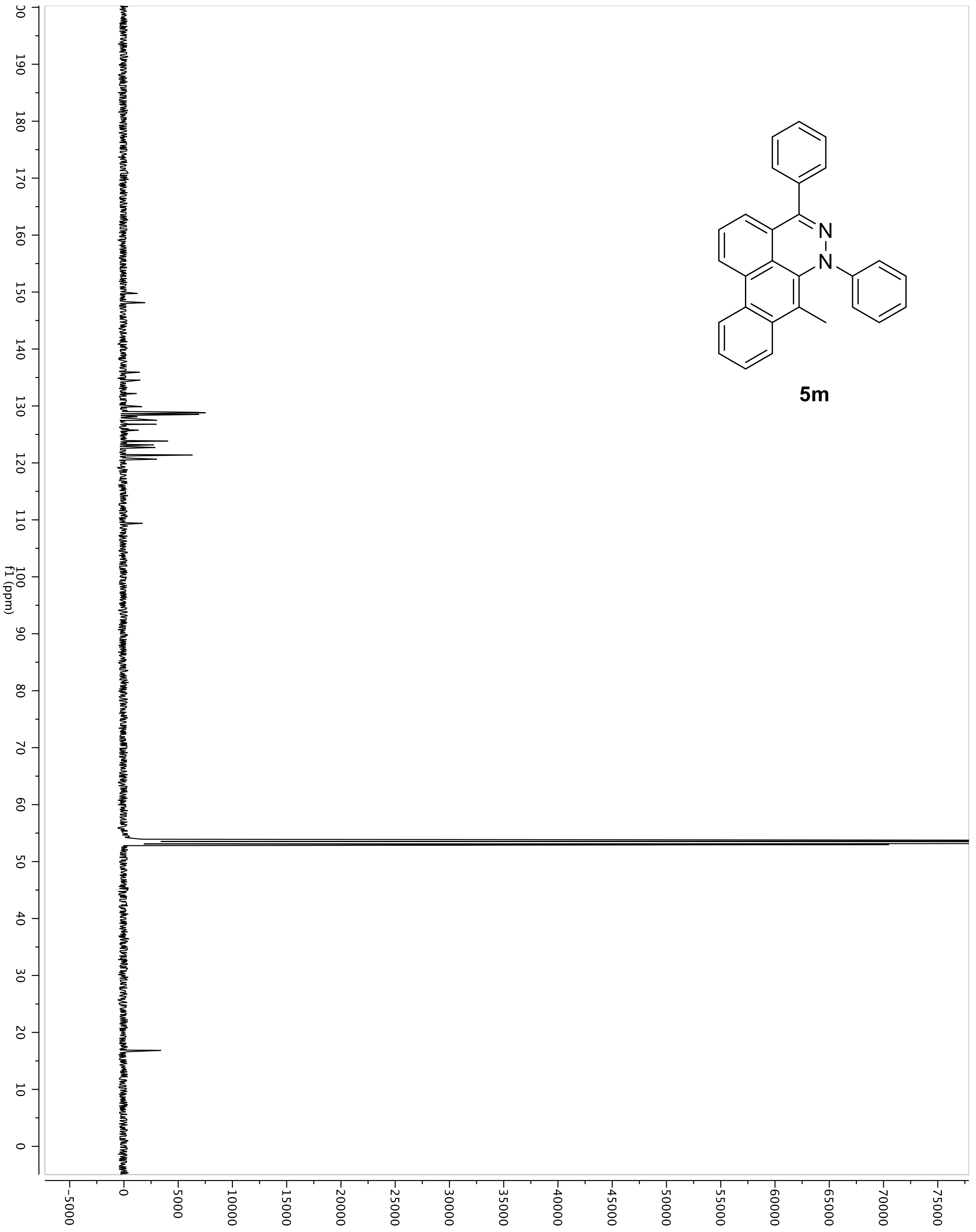

^{1}H NMR spectrum of **5n** in CD_2Cl_2 (500 MHz).

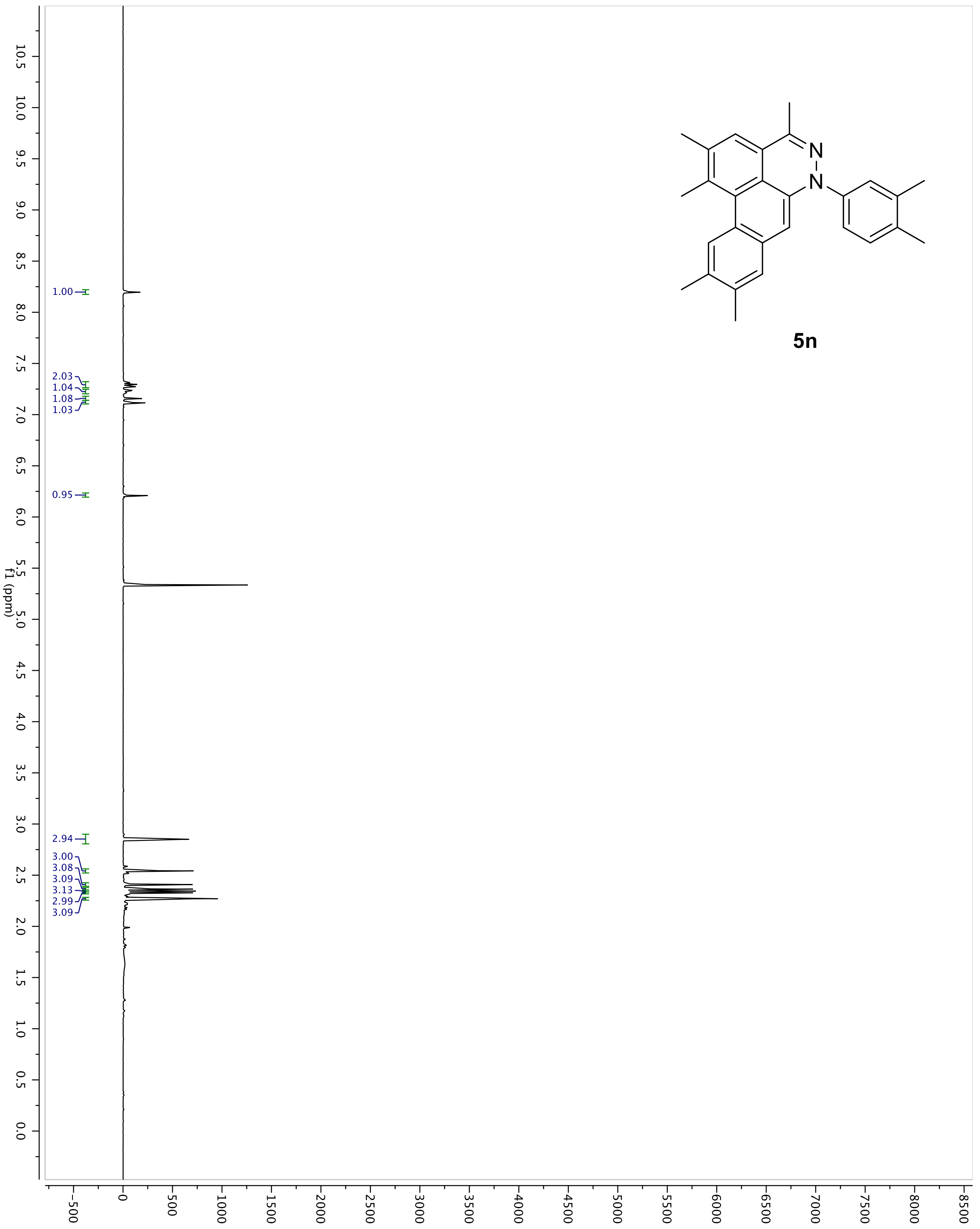

^{13}C NMR spectrum of **5n** in CD_2Cl_2 (125 MHz).

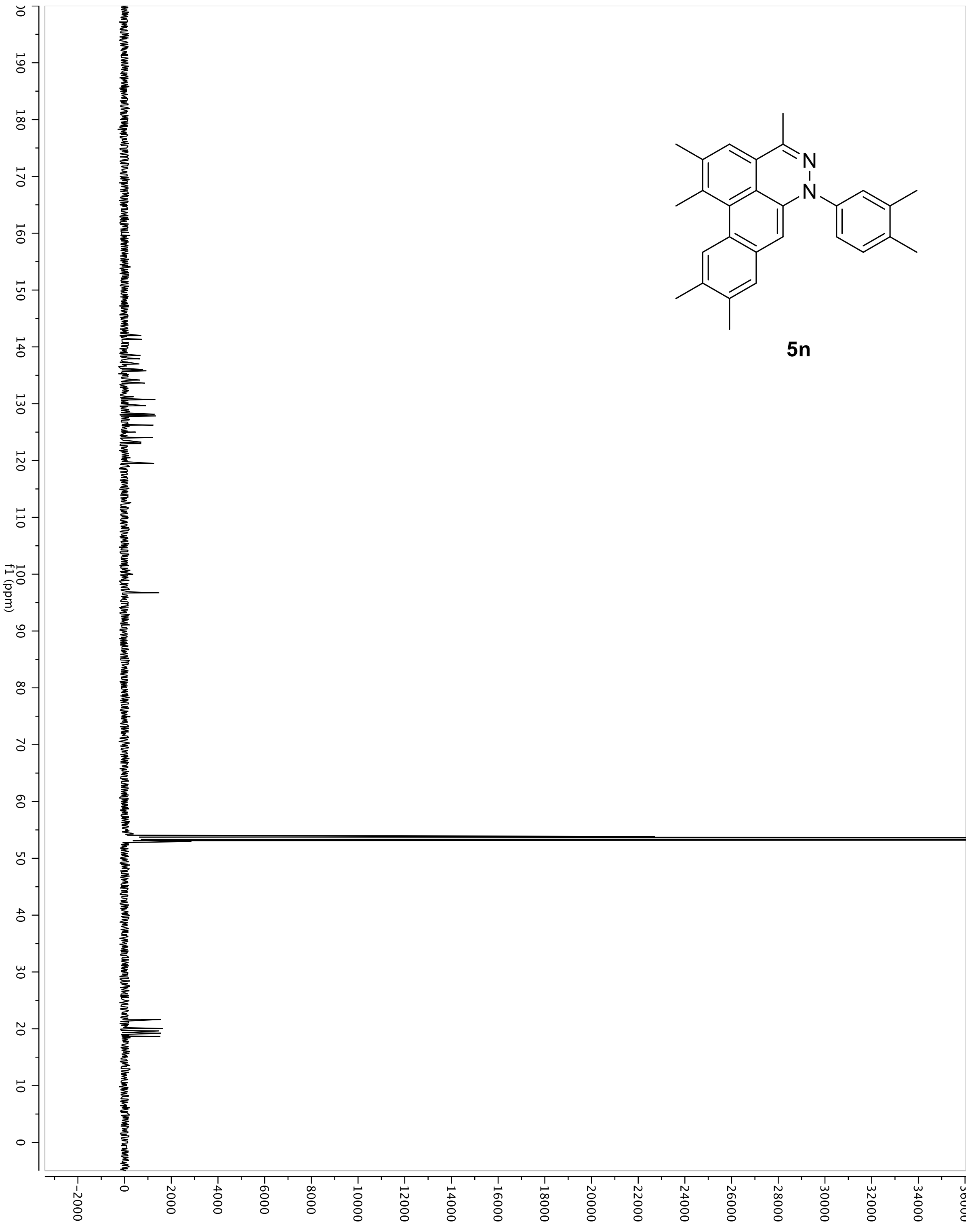

^{1}H NMR spectrum of **5o** in CD_2Cl_2 (500 MHz).

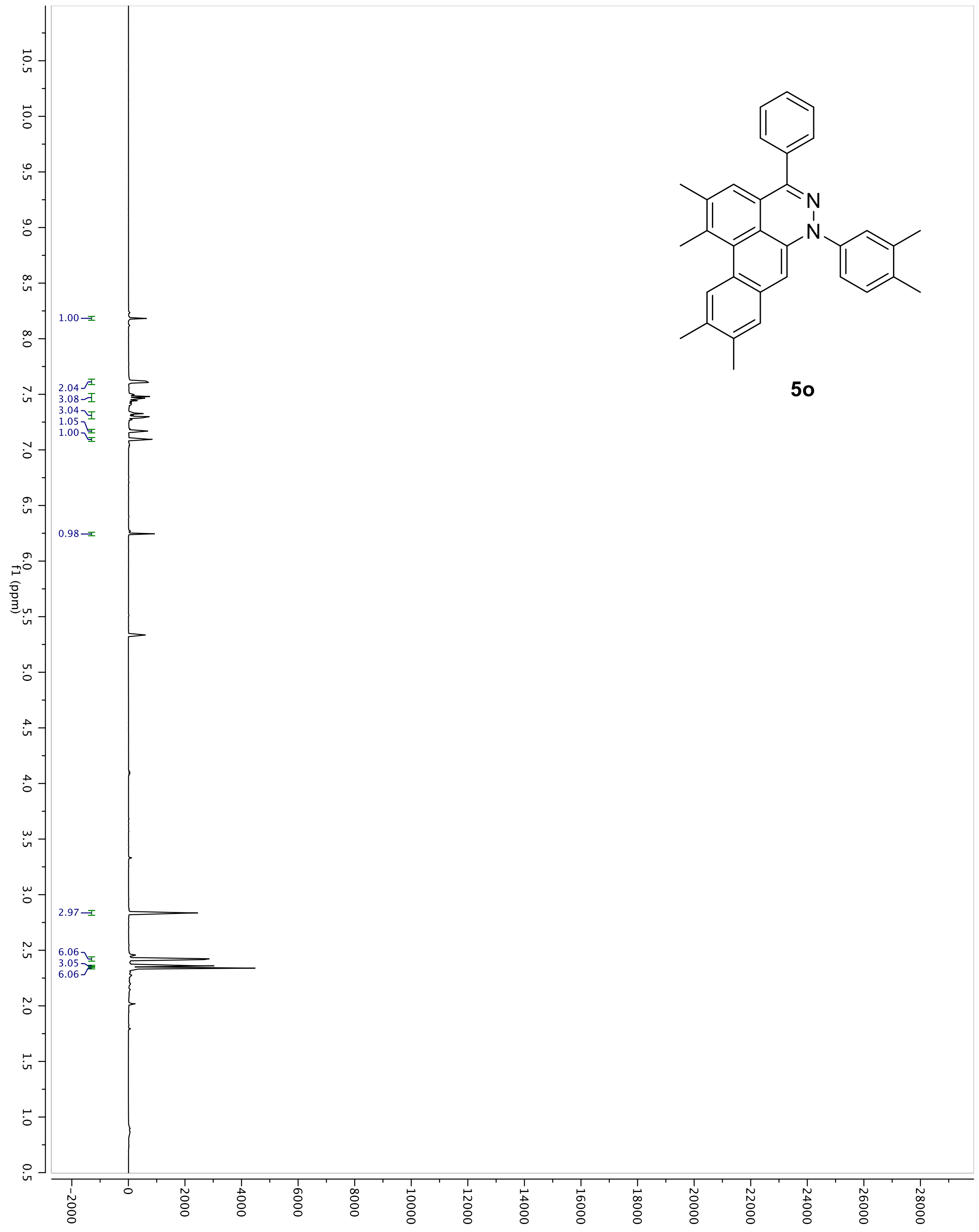

^{13}C NMR spectrum of **5o** in CD_2Cl_2 (125 MHz).

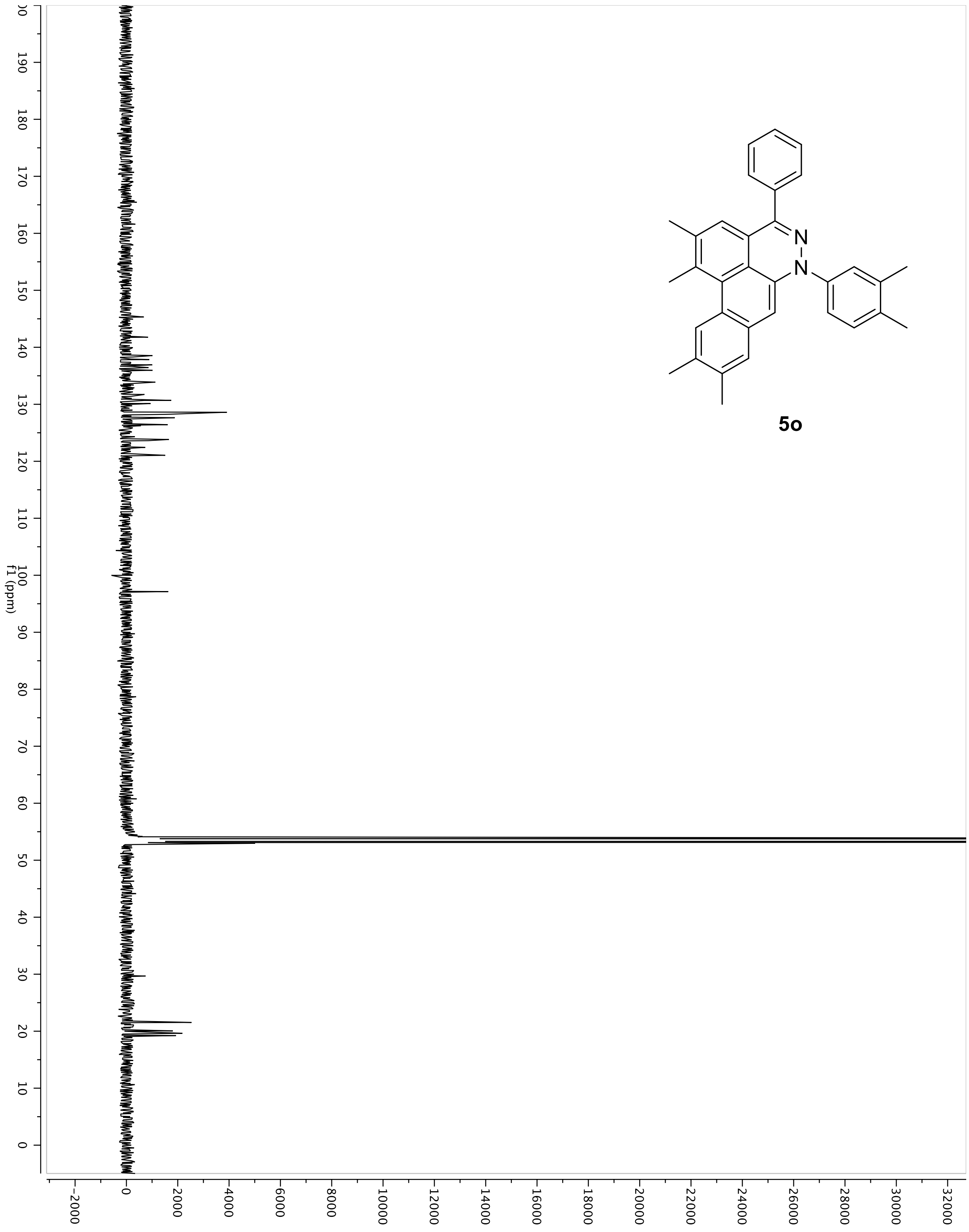

^{1}H NMR spectrum of **5p** in CD_2Cl_2 (500 MHz).

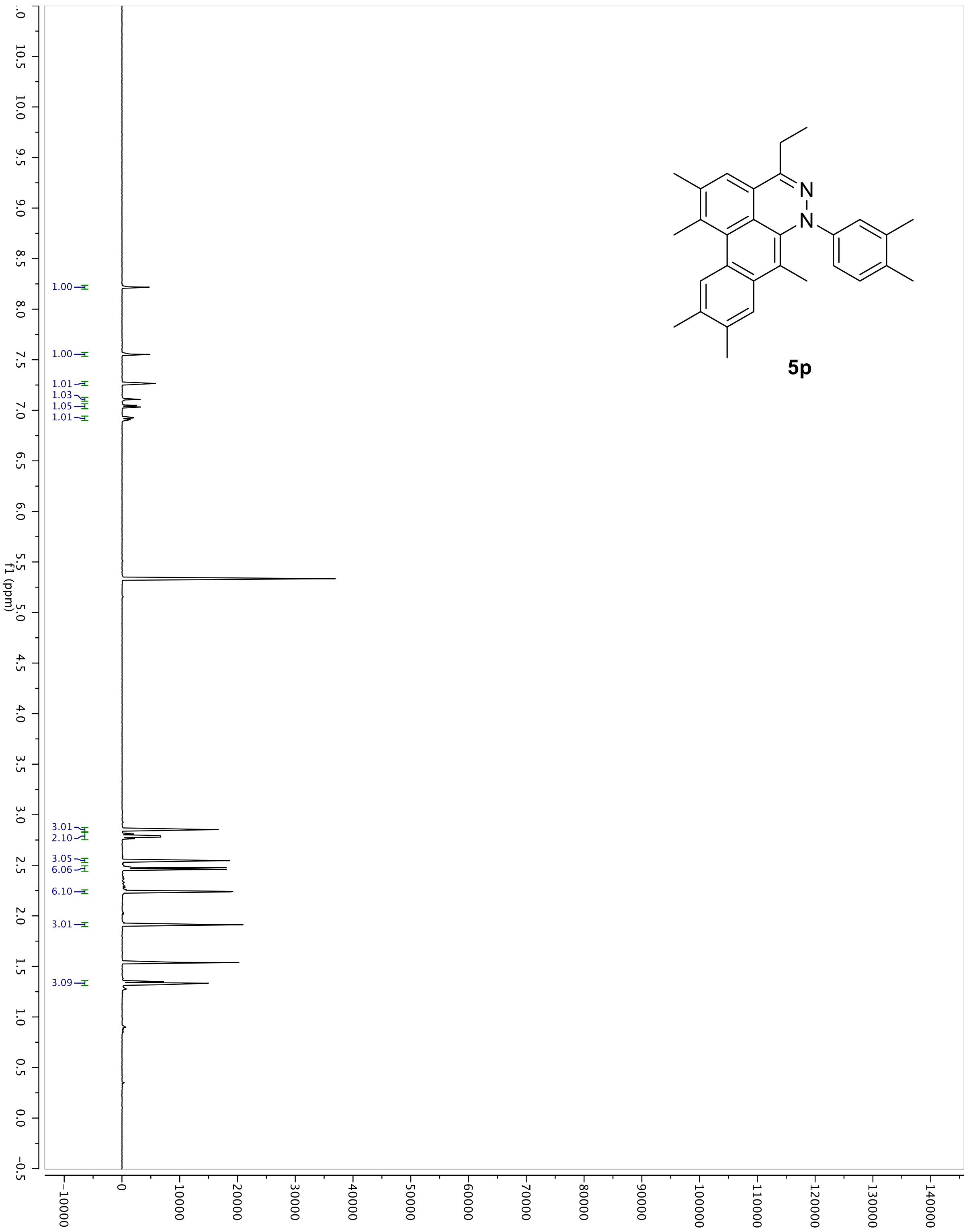

^{13}C NMR spectrum of **5p** in CD_2Cl_2 (125 MHz).

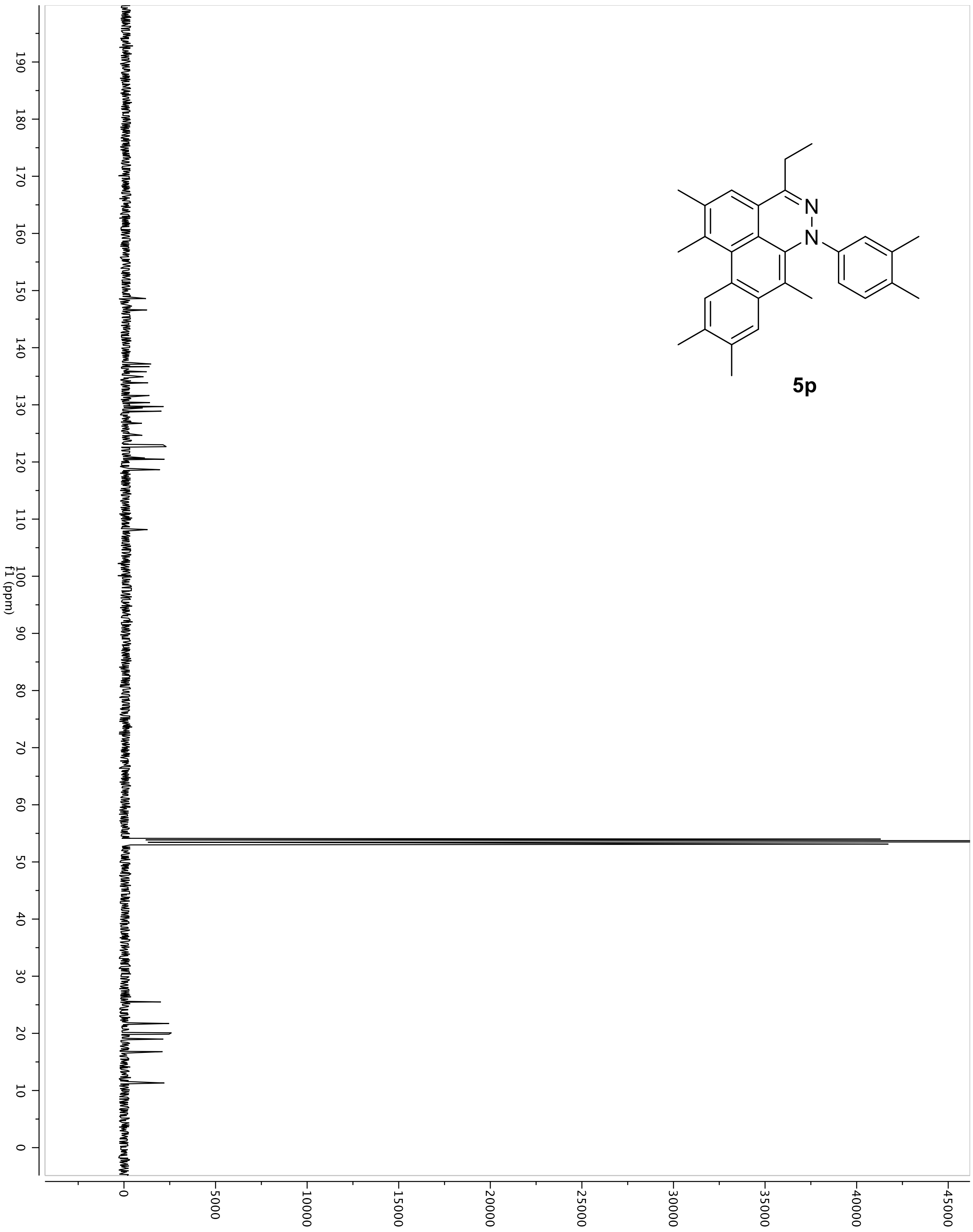

^{1}H NMR spectrum of **3m** in CD_2Cl_2 (500 MHz).

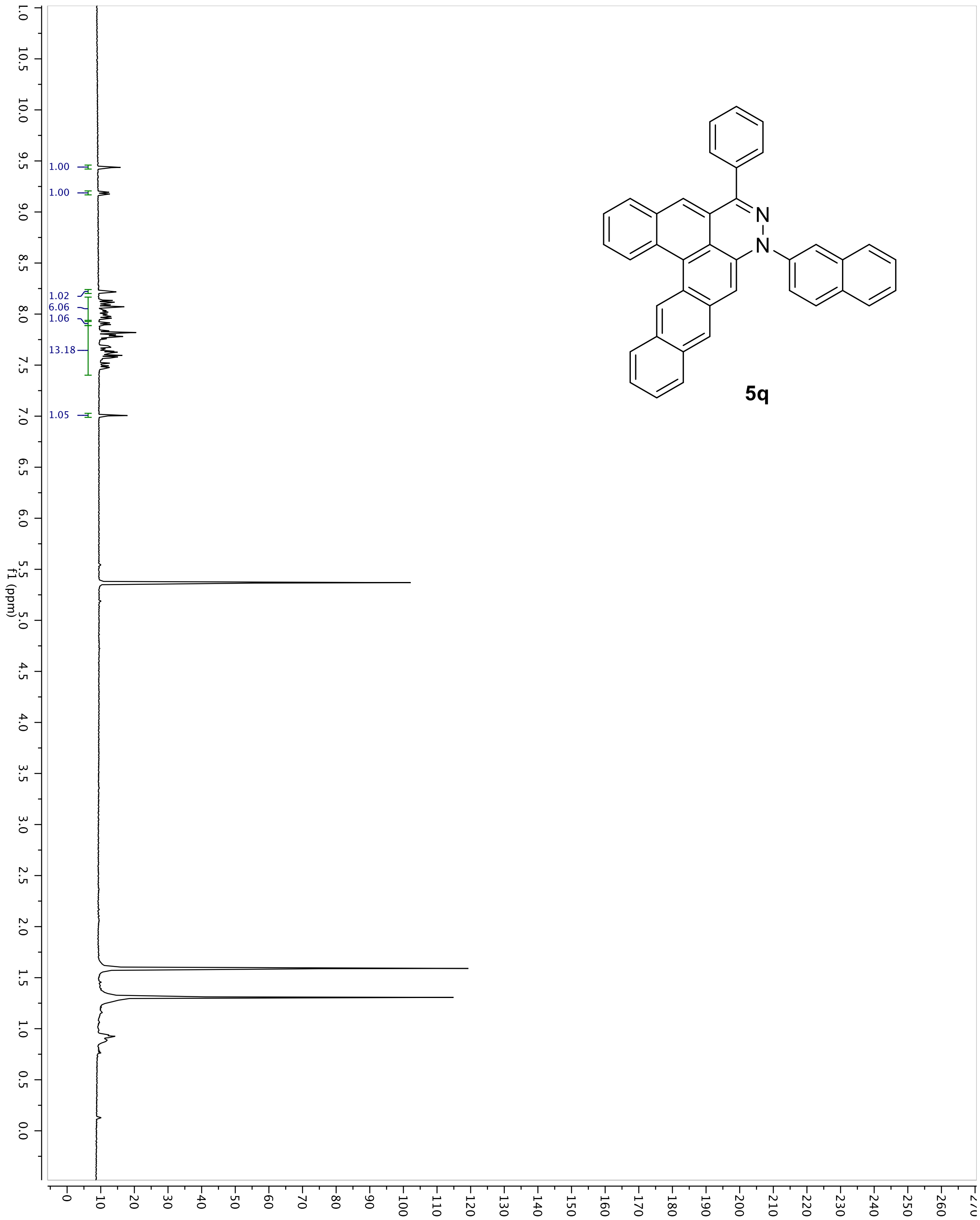

^{13}C NMR spectrum of **3m** in CD_2Cl_2 (125 MHz).

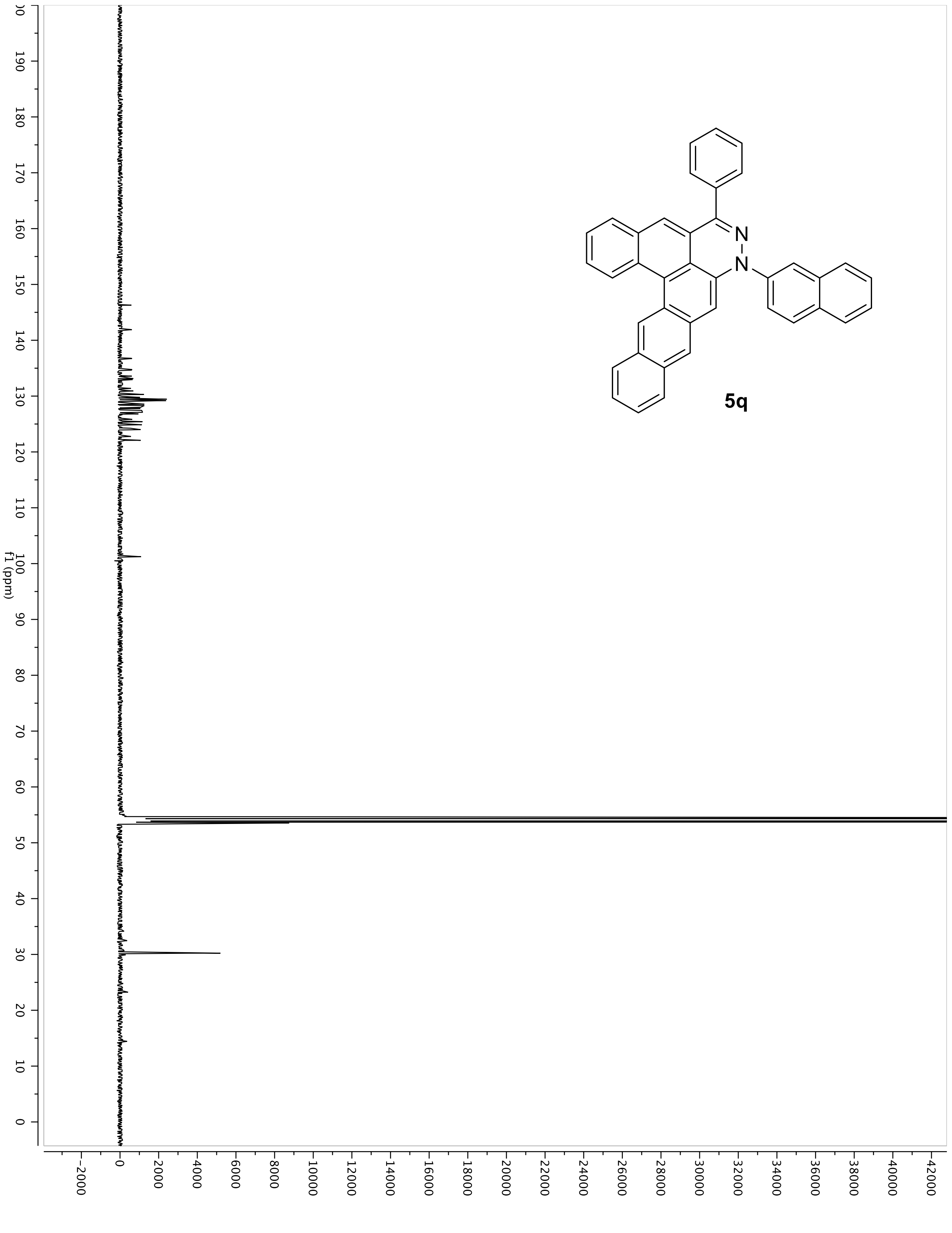

HSQC spectrum of **3m** in CD_2Cl_2.

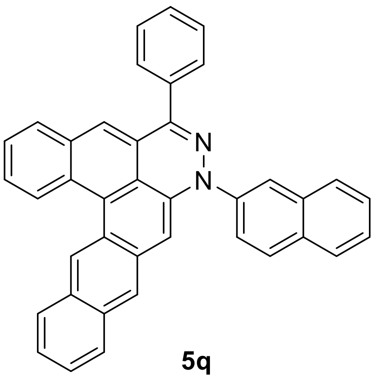

5q

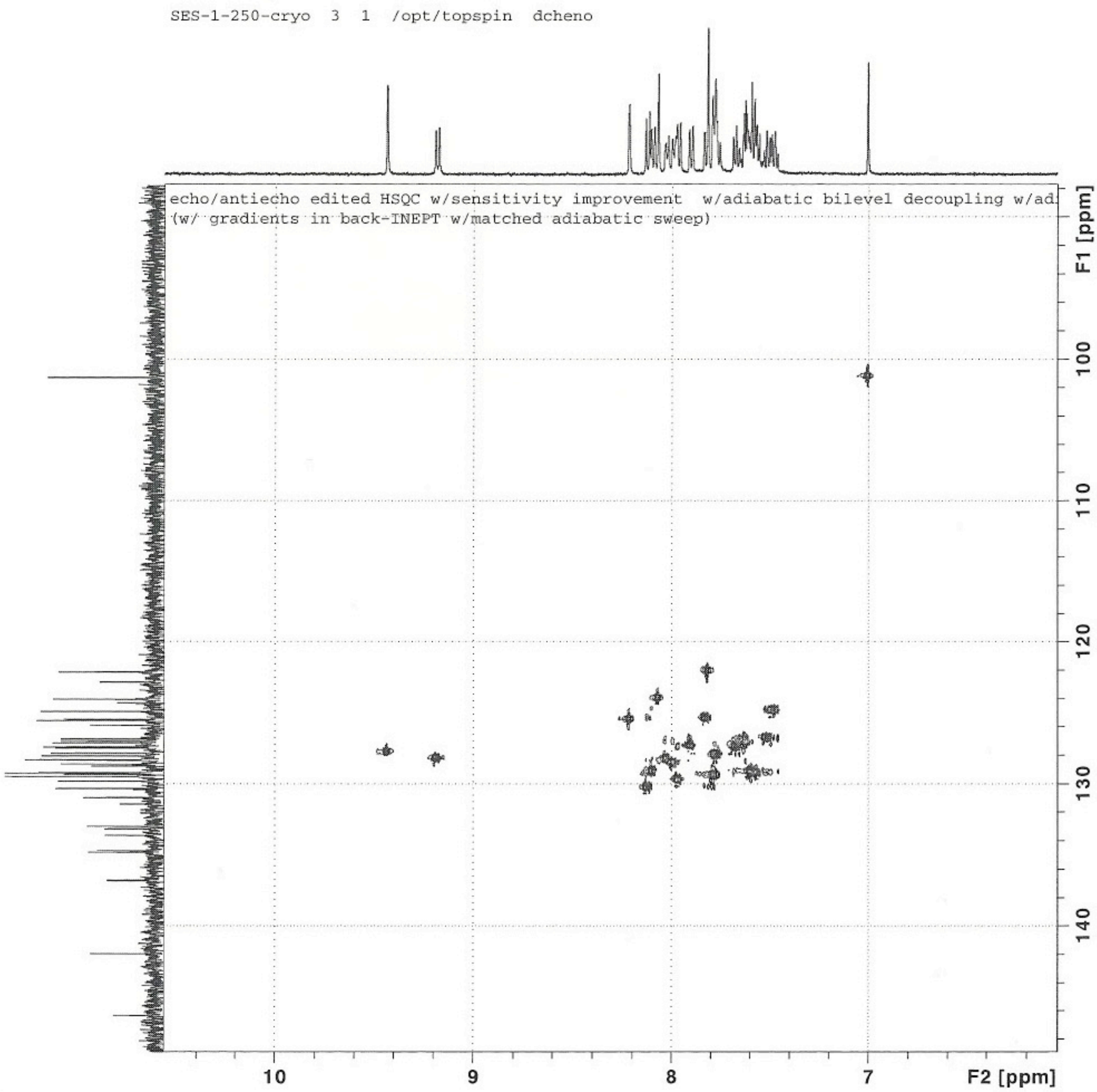

Expanded partial HSQC spectrum of **3m** in CD_2Cl_2.

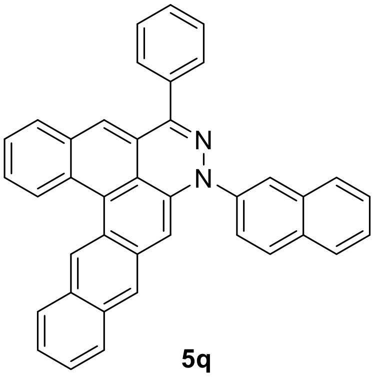

5q

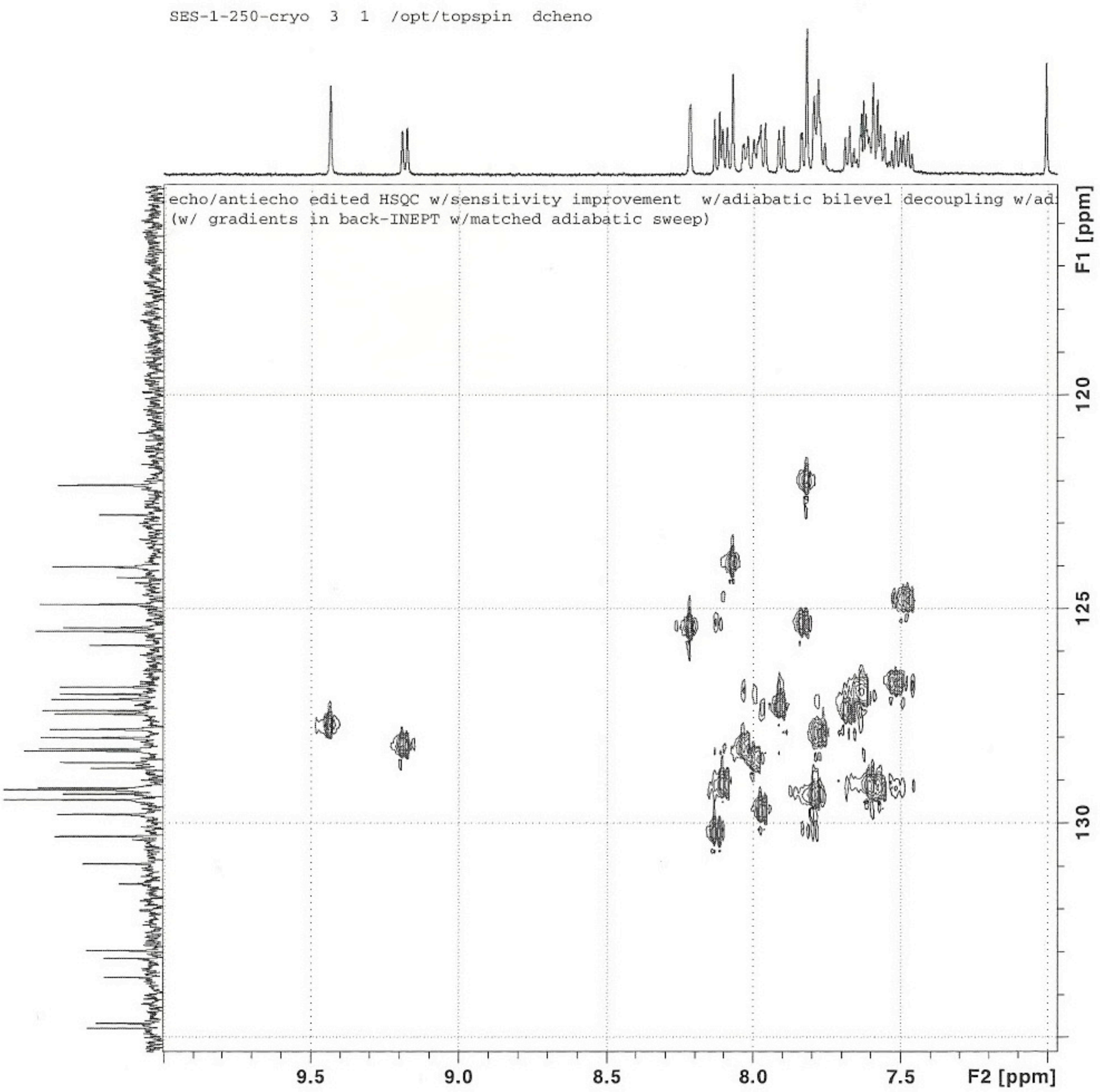

HMBC spectrum of **3m** in CD_2Cl_2.

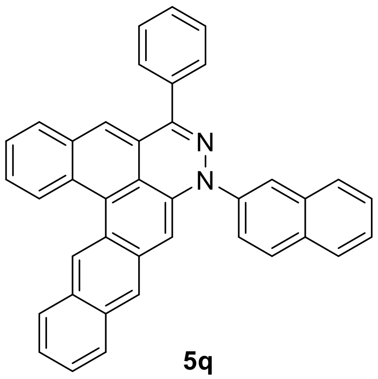

5q

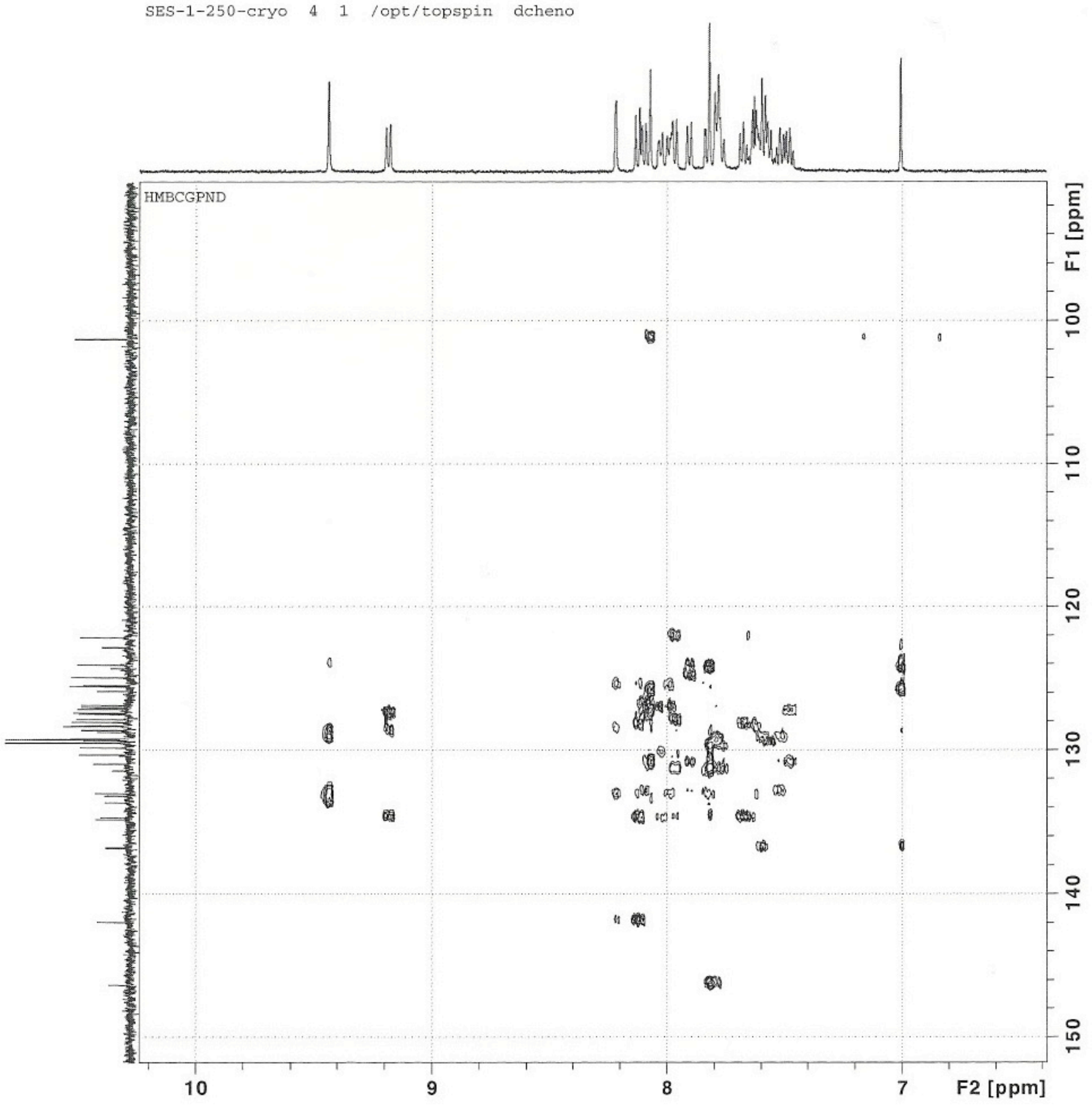

Expanded partial HMBC spectrum of **3m** in CD_2Cl_2.

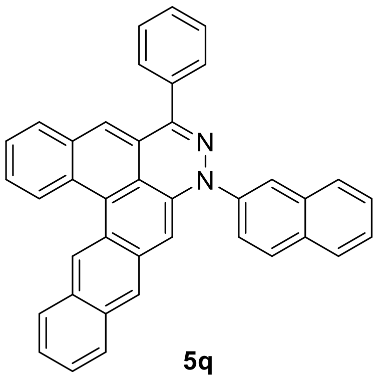

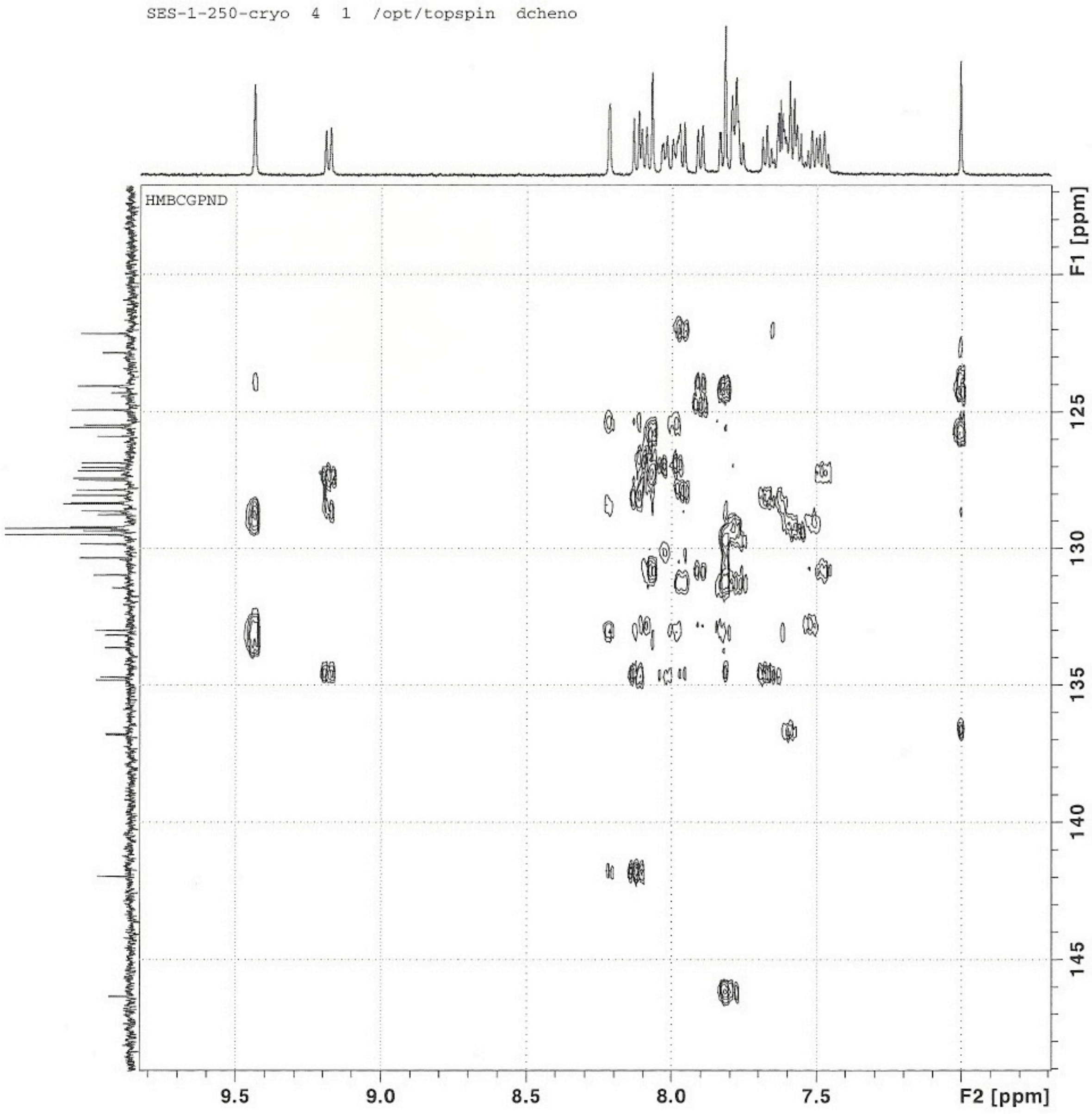

TOCSY spectrum of **3m** in CD_2Cl_2.

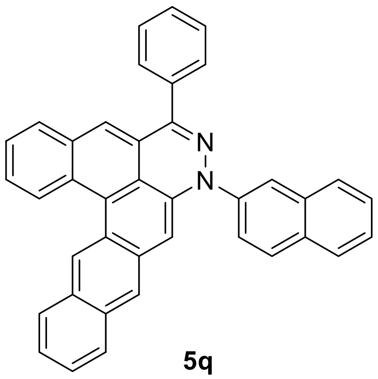

5q

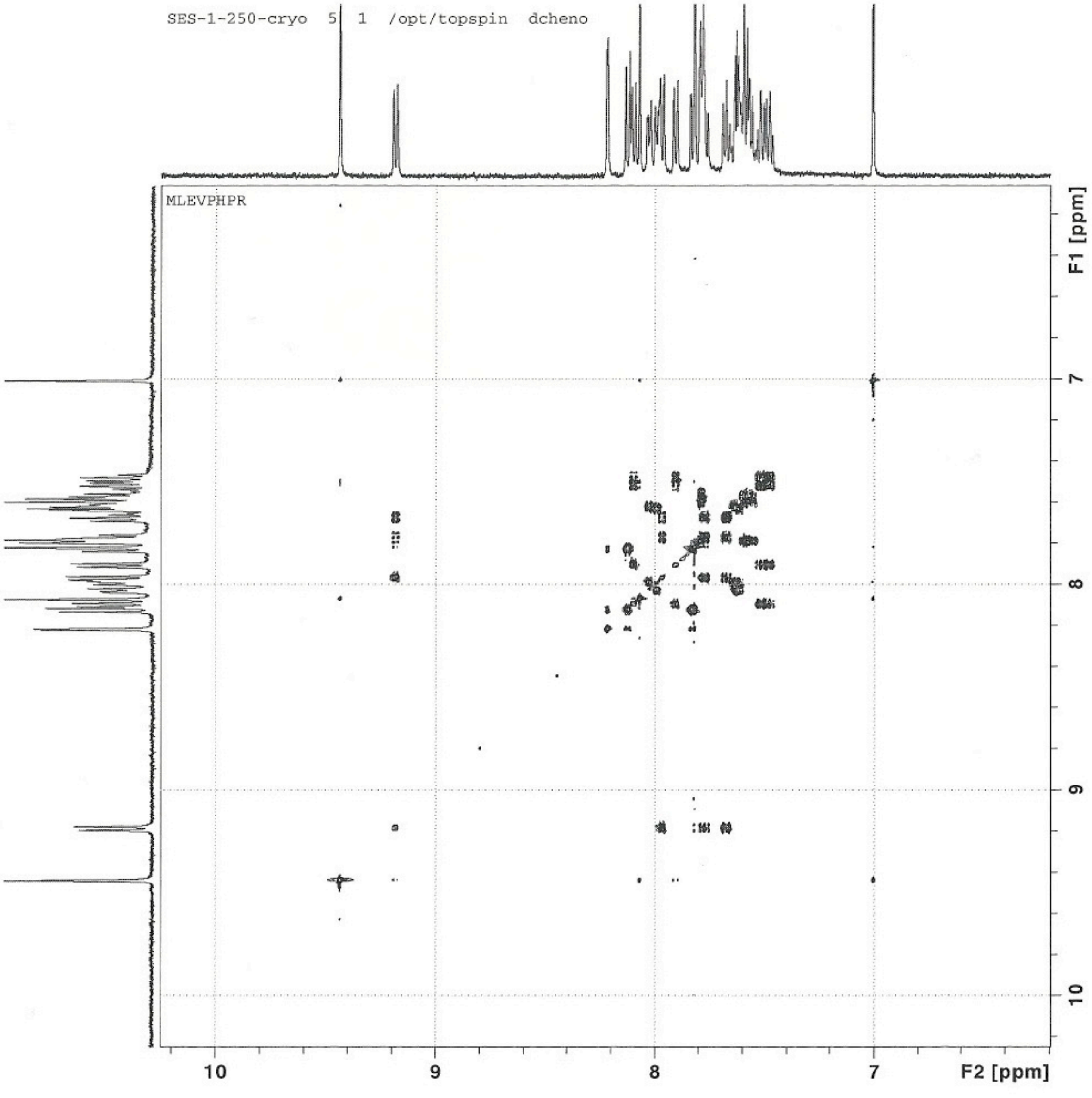

1st Expanded partial TOCSY spectrum of **3m** in CD_2Cl_2.

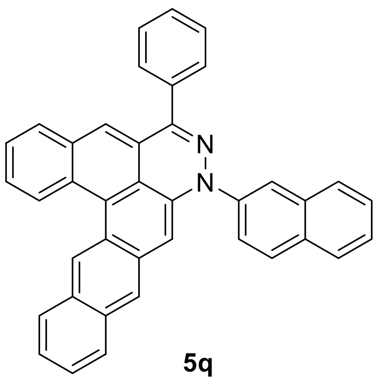

5q

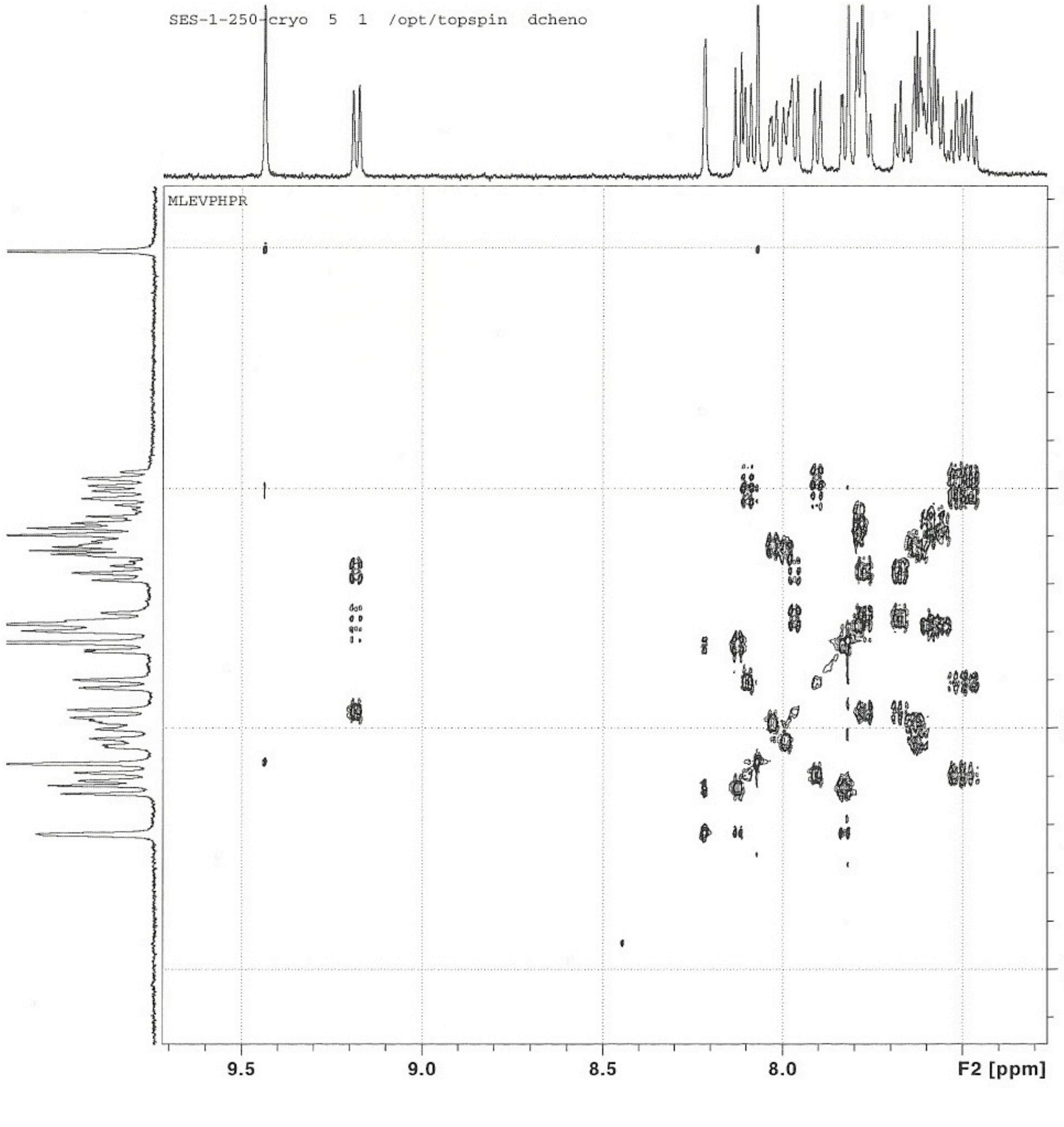

2nd Expanded partial TOCSY spectrum of **3m** in CD_2Cl_2.

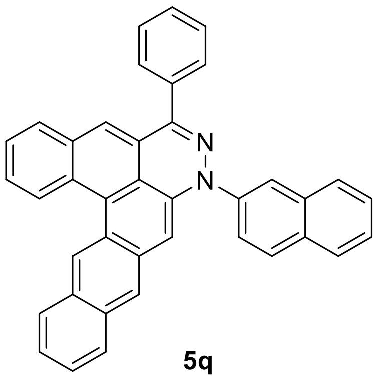

5q

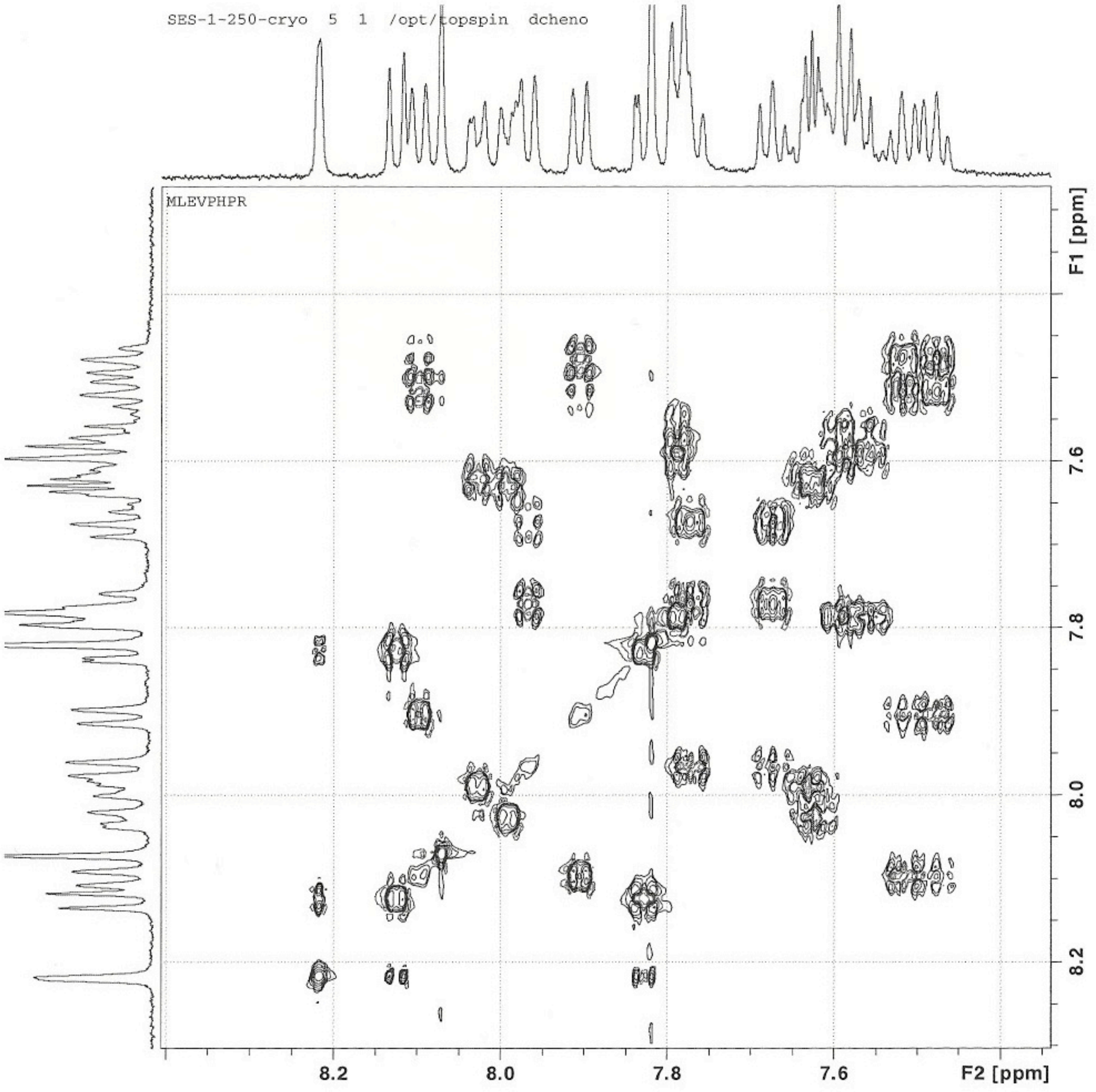

COSY spectrum of **3m** in CD_2Cl_2.

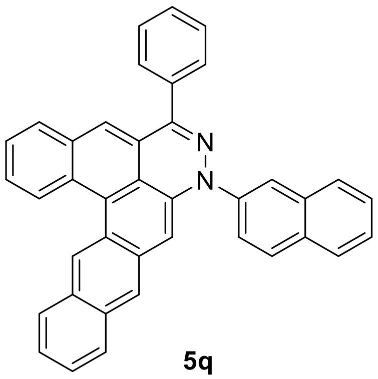

5q

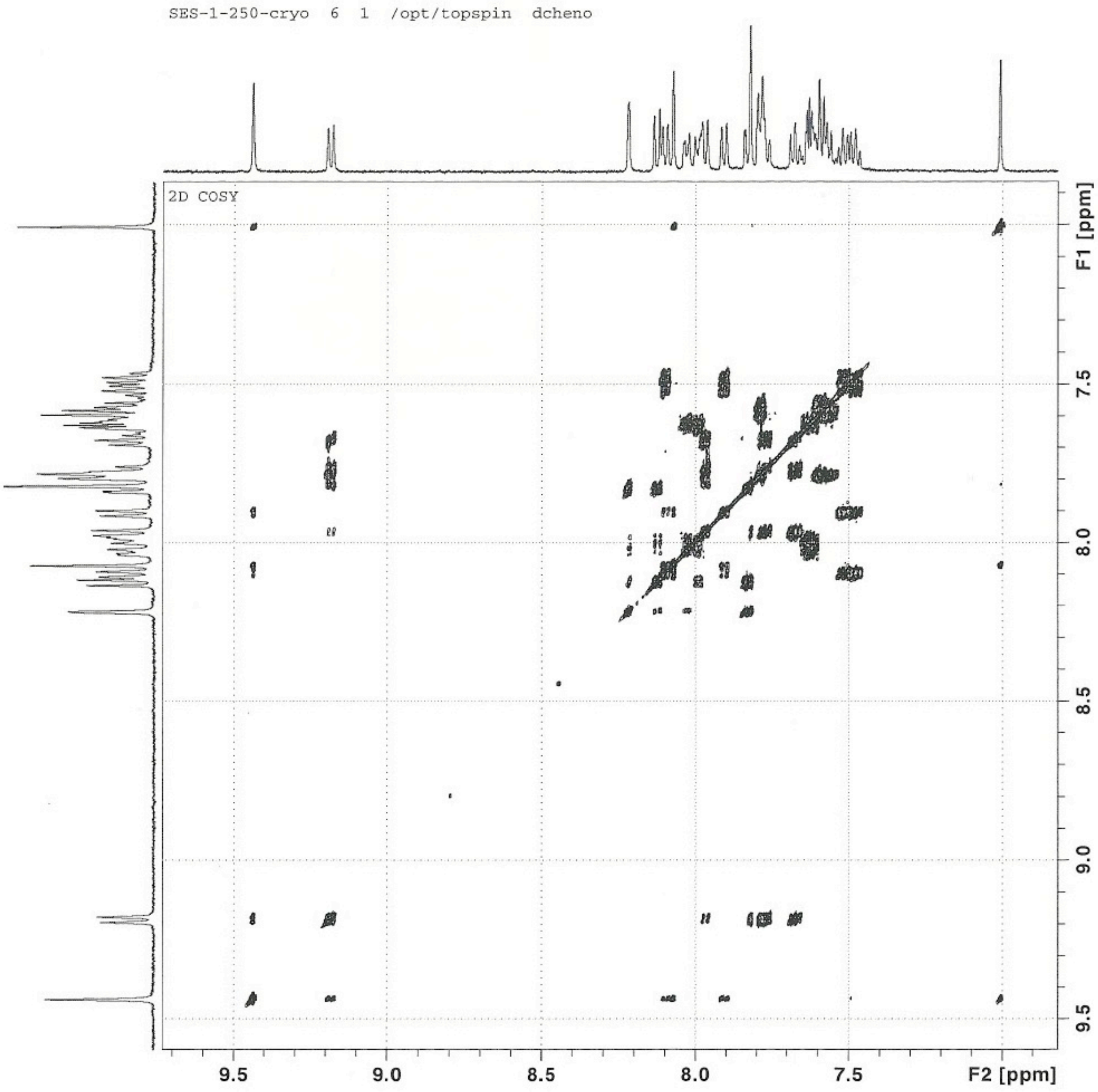

^{1}H NMR spectrum of **6** in $CDCl_3$ (500 MHz).

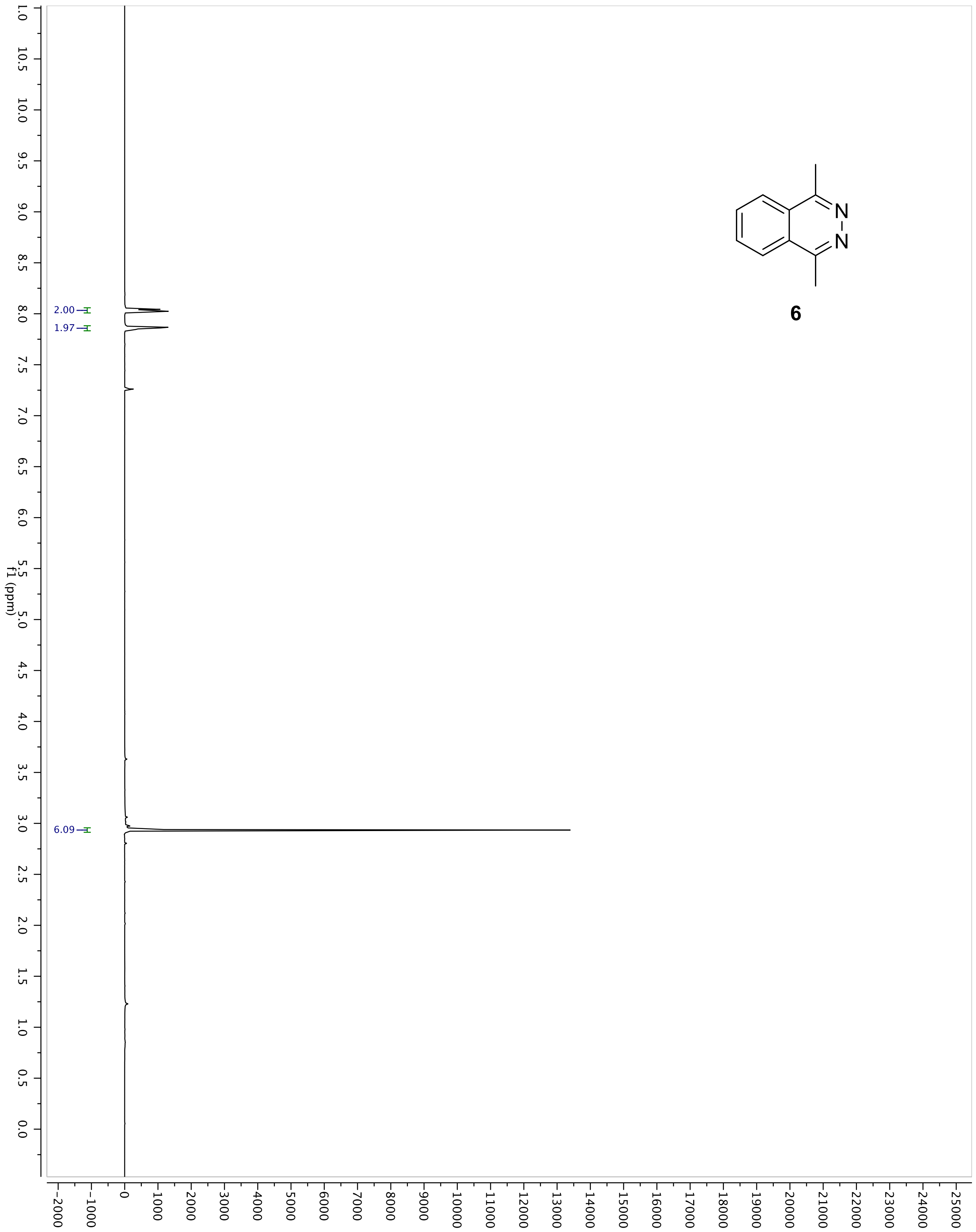

^{13}C NMR spectrum of **6** in $CDCl_3$ (125 MHz).

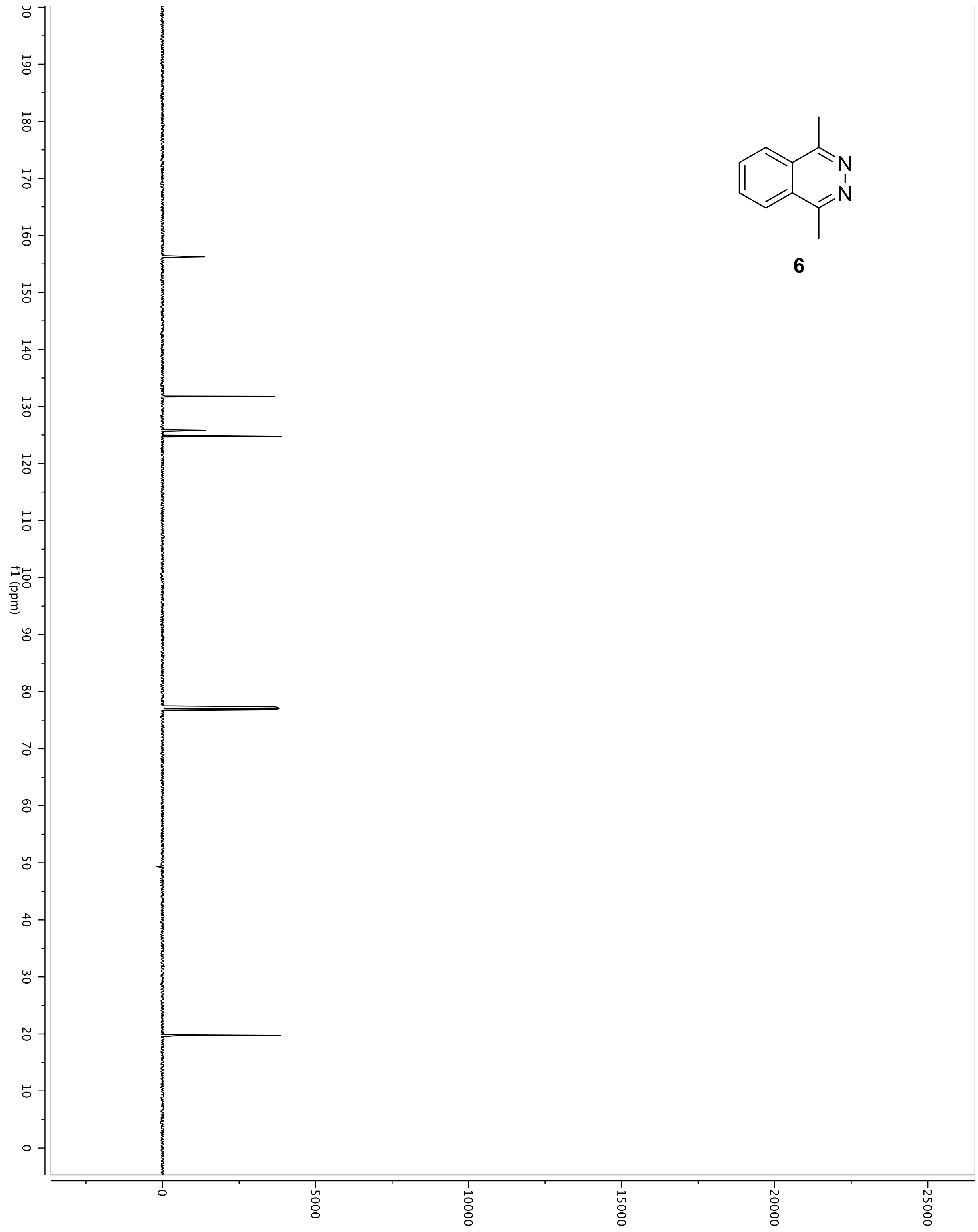

^{1}H NMR spectrum of **7** in $CDCl_3$ (500 MHz).

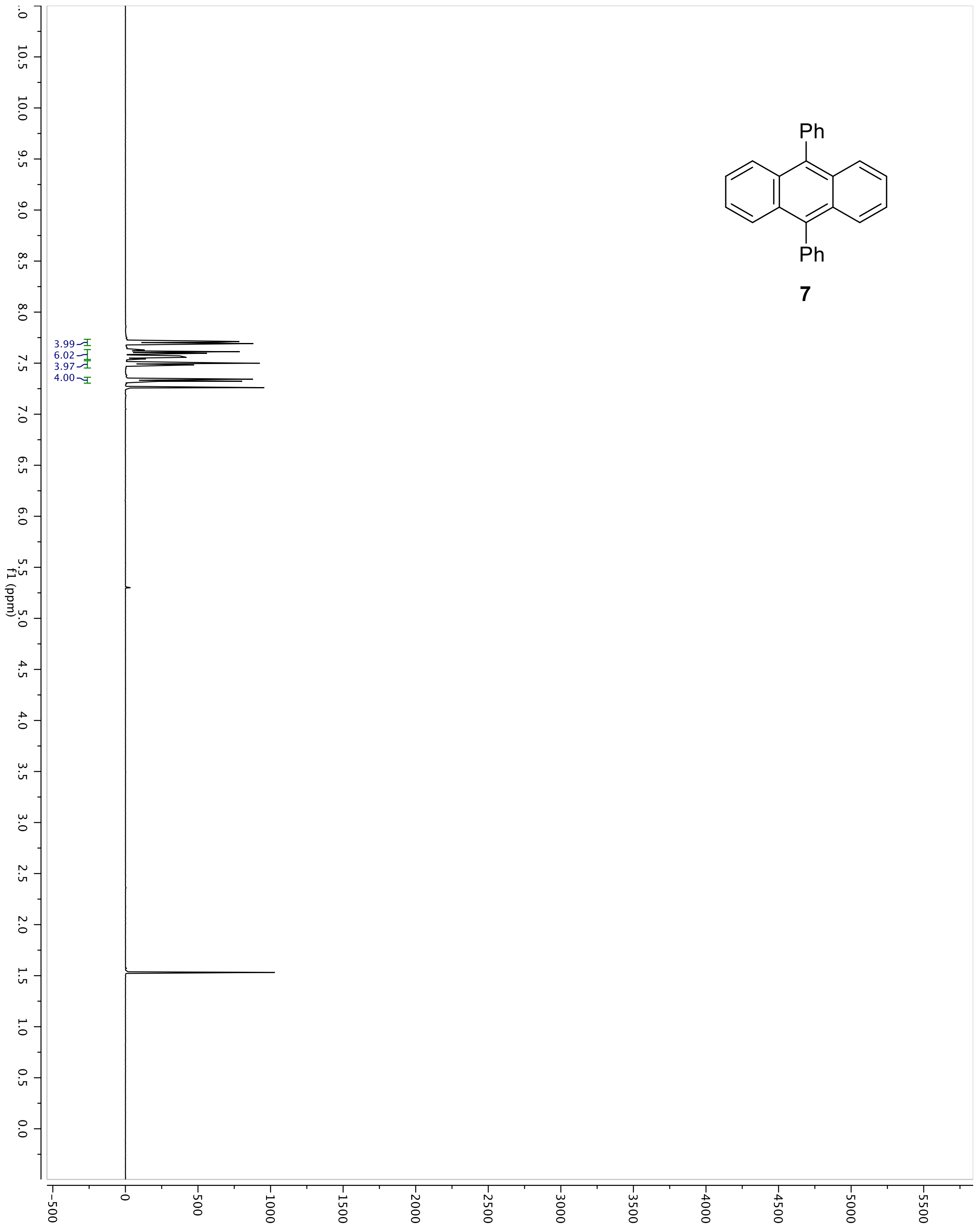

Absorption, emission, and exitation spectra of **5a** at 24 ˚C in CH_2Cl_2.

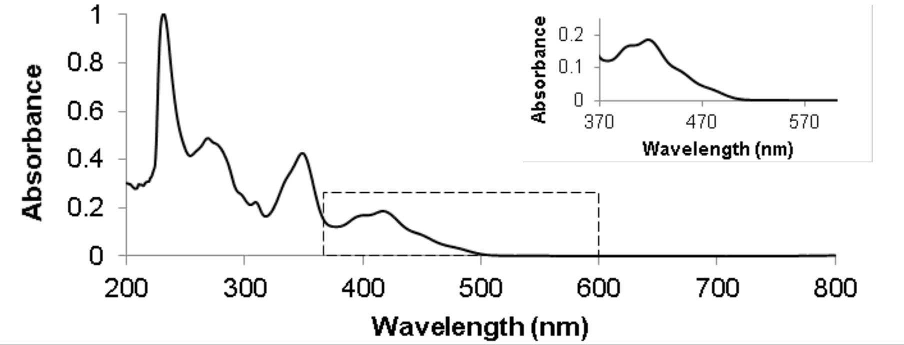

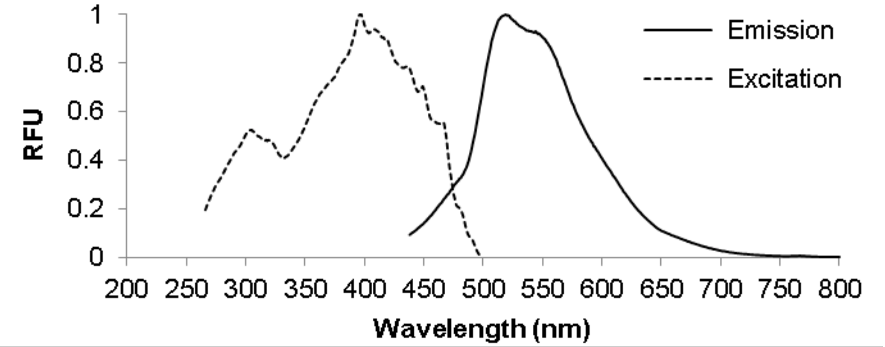

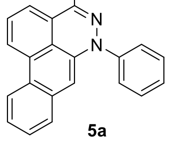

5a

Solvent: CH_2Cl_2
Quantum Yield (Φ): 1.5 %
Extinction Coefficient (ε):
7362 ± 164 (417 nm)
16847 ± 348 (349 nm)
18285 ± 419 (269 nm)
34084 ± 214 (231 nm)

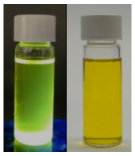

Solutions of **3a** in CH_2Cl_2 (0.4 mM) under ambient light (right) and irradiated at 365 nm with a UV lamp (left)

Absorption, emission, and excitation spectra of **5a** at 24 ˚C in CH_2Cl_2/TFA.

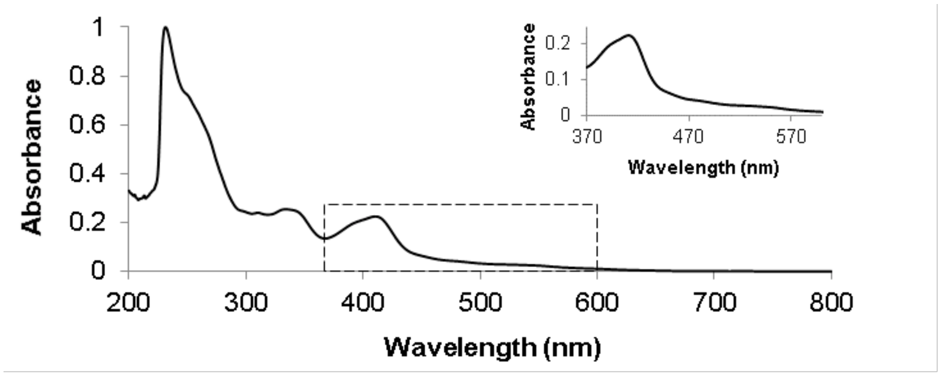

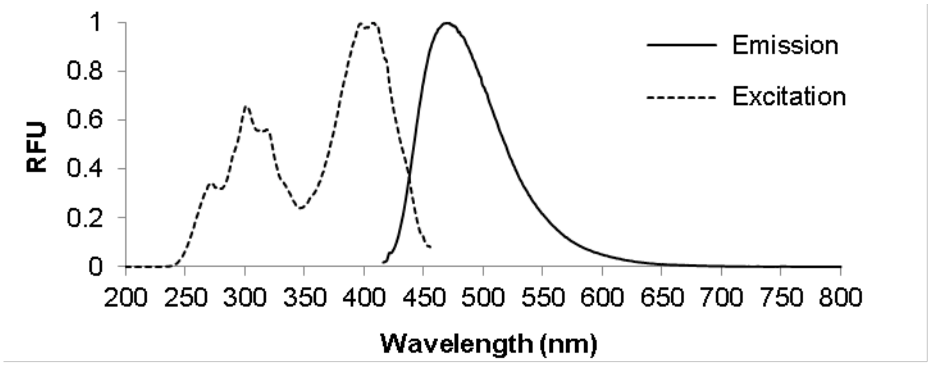

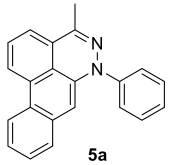

5a

Solvent: CH_2Cl_2/TFA
Quantum Yield (Φ): 27.7 %
Extinction Coefficient (ε):
7378 ± 181 (411 nm)
46894 ± 370 (230 nm)

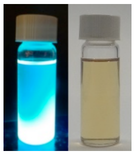

Solutions of **3a** in CH_2Cl_2/TFA (0.4 mM) under ambient light (right) and irradiated at 365 nm with a UV lamp (left)

Absorption, emission, and excitation spectra of **5b** at 24 ˚C in CH_2Cl_2.

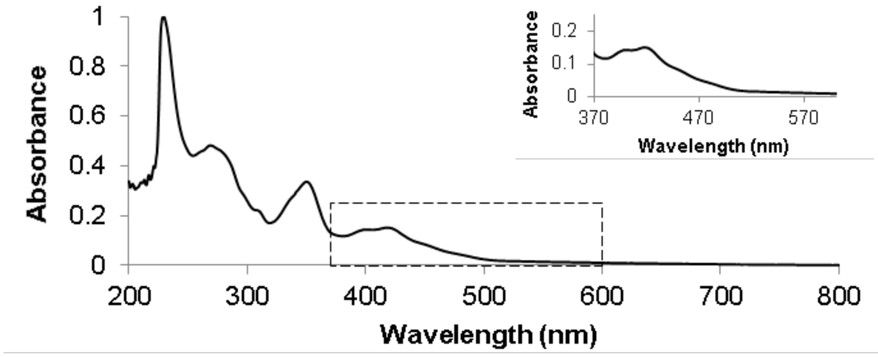

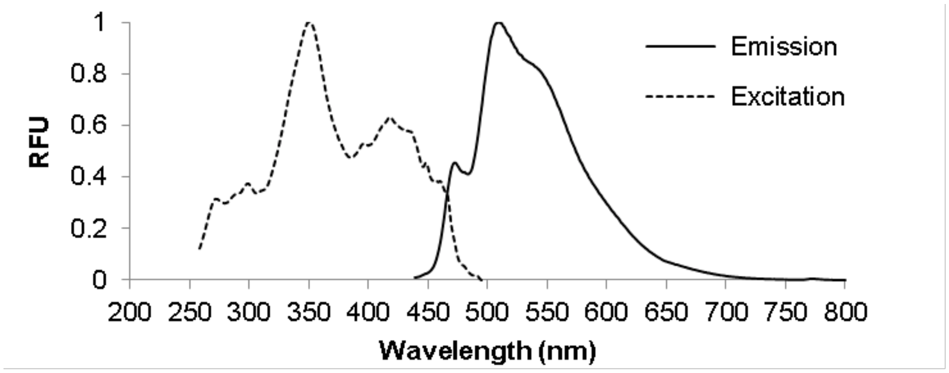

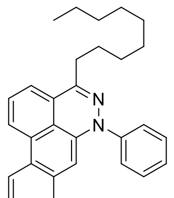

5b

Solvent: CH_2Cl_2
Quantum Yield (Φ): 1.2 %
Extinction Coefficient (ε):
9205 ± 358 (419 nm)
15954 ± 109 (350 nm)
17336 ± 601 (269 nm)
34072 ± 342 (229 nm)

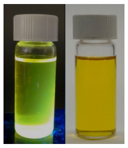

Solutions of **3b** in CH_2Cl_2 (0.4 mM) under ambient light (right) and irradiated at 365 nm with a UV lamp (left)

Absorption, emission, and excitation spectra of **5b** at 24 ˚C in CH_2Cl_2/TFA.

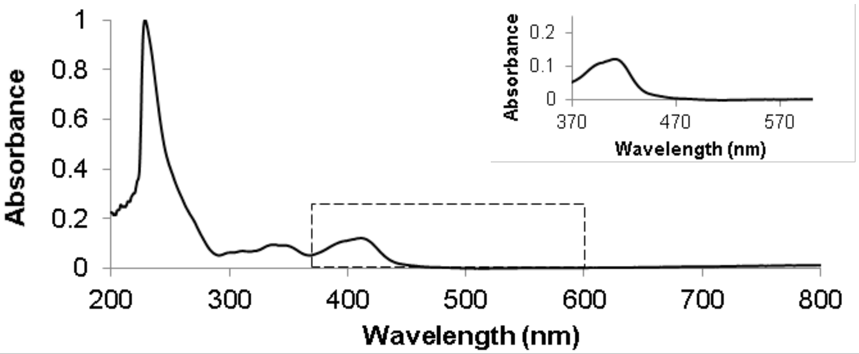

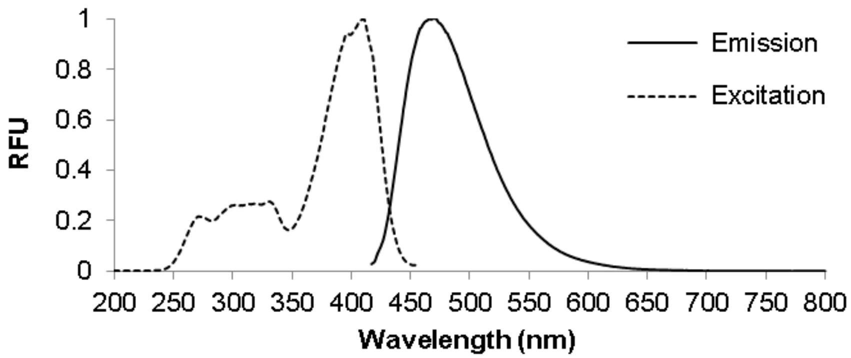

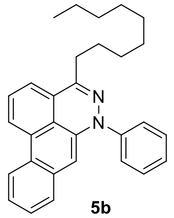

5b

Solvent: CH_2Cl_2/TFA
Quantum Yield (Φ): 6.6 %
Extinction Coefficient (ε):
7103 ± 16 (419 nm)
42445 ± 459 (229 nm)

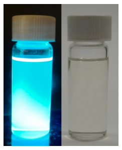

Solutions of **3b** in CH_2Cl_2/TFA (0.4 mM) under ambient light (right) and irradiated at 365 nm with a UV lamp (left)

Absorption, emission, and excitation spectra of **5d** at 24 ˚C in CH_2Cl_2.

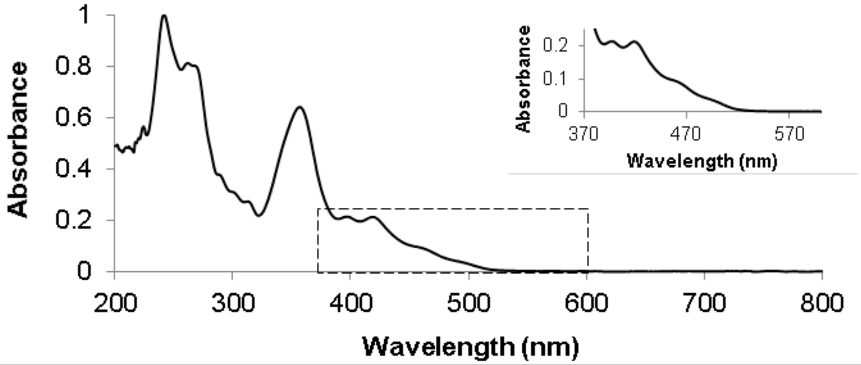

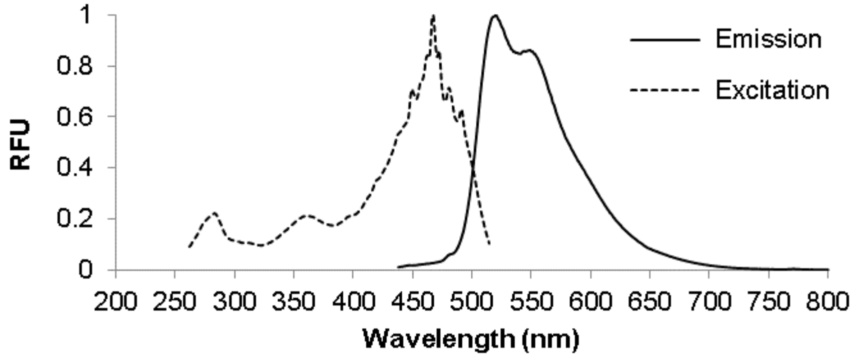

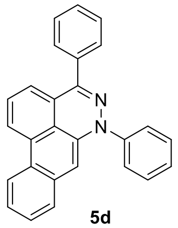

5d

Solvent: CH_2Cl_2
Quantum Yield (Φ): 0.7 %
Extinction Coefficient (ε):
4478 ± 140 (419 nm)
13669 ± 371 (357 nm)
18217 ± 536 (261 nm)
25145 ± 719 (242 nm)

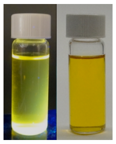

Solutions of **3c** in CH_2Cl_2 (0.4 mM) under ambient light (right) and irradiated at 365 nm with a UV lamp (left)

Absorption, emission, and excitation spectra of **5d** at 24 ˚C in CH_2Cl_2/TFA.

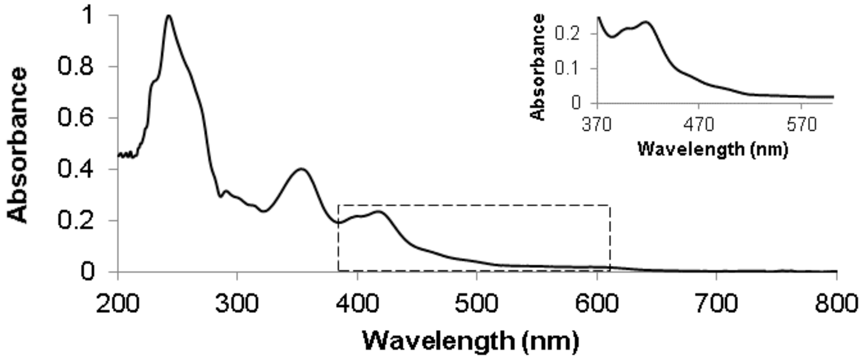

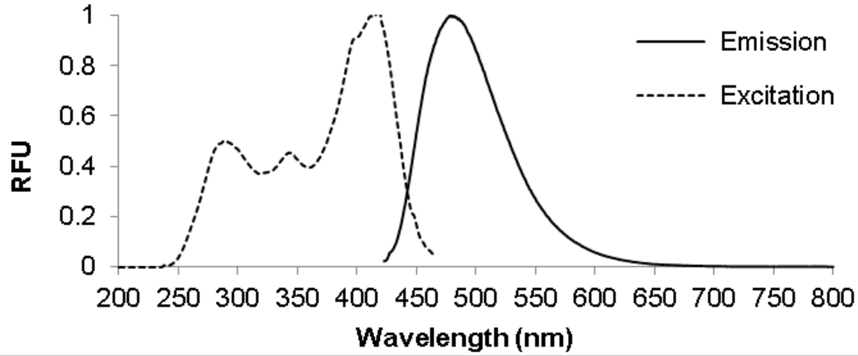

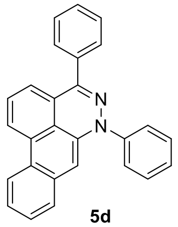

5d

Solvent: CH_2Cl_2/TFA
Quantum Yield (Φ): 8.1 %
Extinction Coefficient (ε):
7346 ± 19 (417 nm)
25976 ± 371 (404 nm)

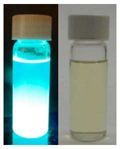

Solutions of **3c** in CH_2Cl_2/TFA (0.4 mM) under ambient light (right) and irradiated at 365 nm with a UV lamp (left)

Absorption, emission, and excitation spectra of **5e** at 24 ˚C in CH_2Cl_2.

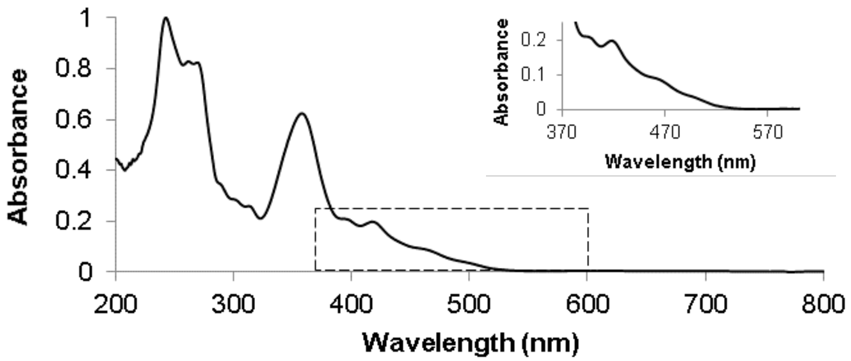

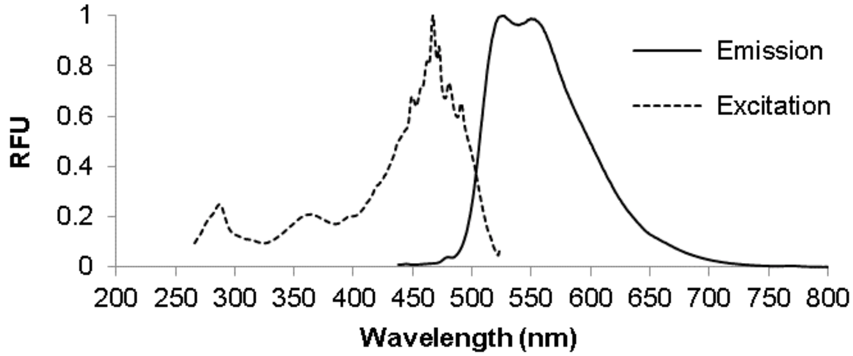

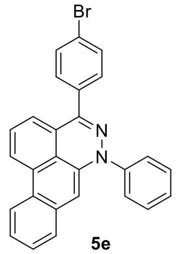

5e

Solvent: CH_2Cl_2
Quantum Yield (Φ): 0.5 %
Extinction Coefficient (ε):
5949 ± 130 (418 nm)
19021 ± 243 (358 nm)
34833 ± 827 (243 nm)

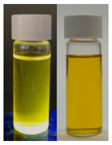

Solutions of **3d** in CH_2Cl_2 (0.4 mM) under ambient light (right) and irradiated at 365 nm with a UV lamp (left)

Absorption, emission, and excitation spectra of **5e** at 24 ˚C in CH_2Cl_2/TFA.

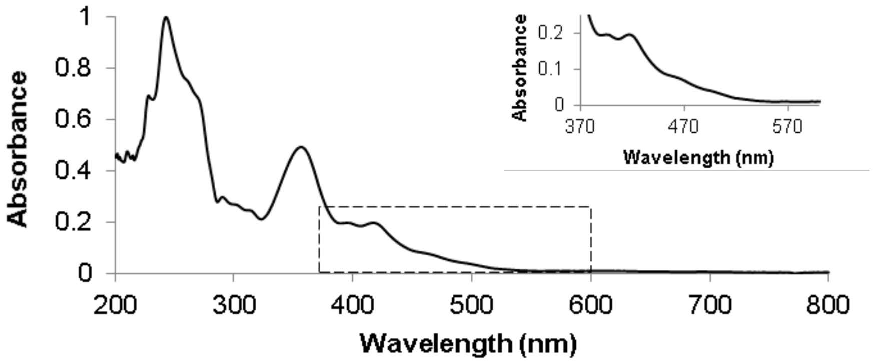

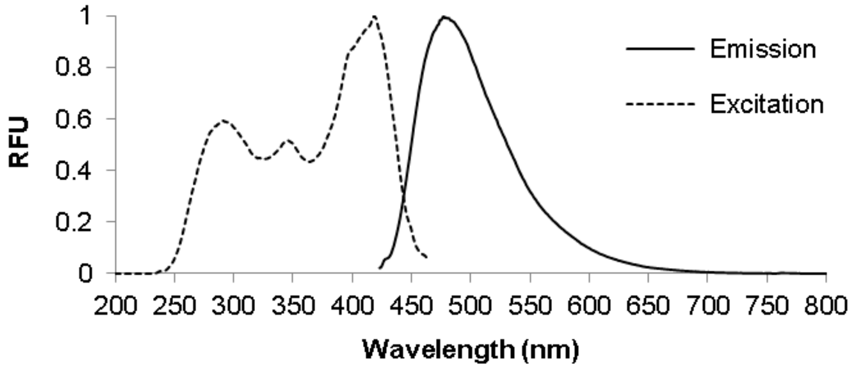

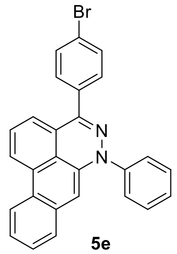

5e

Solvent: CH_2Cl_2/TFA
Quantum Yield (Φ): 6.9 %
Extinction Coefficient (ε):
7960 ± 63 (418 nm)
11769 ± 581 (356 nm)
40935 ± 701 (242 nm)

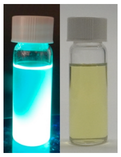

Solutions of **3d** in CH_2Cl_2/TFA (0.4 mM) under ambient light (right) and irradiated at 365 nm with a UV lamp (left)

Absorption, emission, and excitation spectra of **5i** at 24 ˚C in CH_2Cl_2.

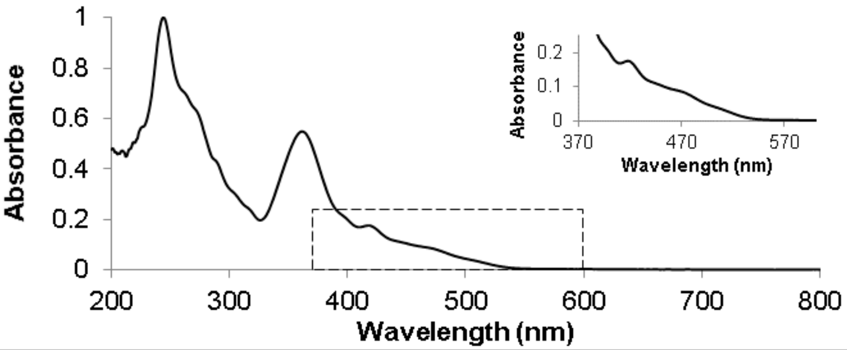

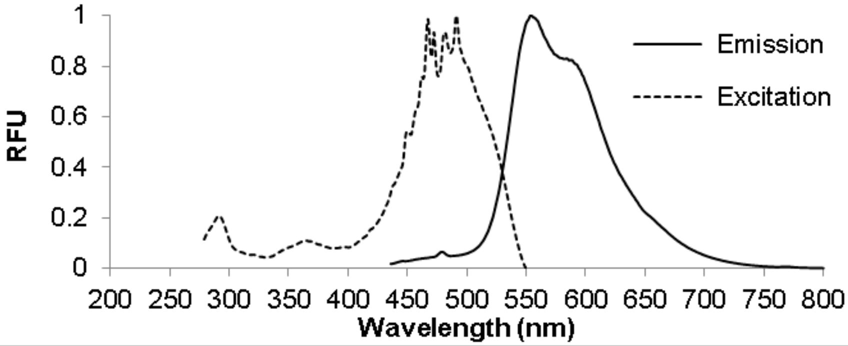

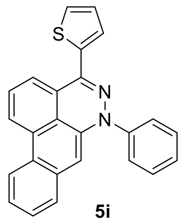

5i

Solvent: CH_2Cl_2
Quantum Yield (Φ): 0.6 %
Extinction Coefficient (ε):
4610 ± 20 (418 nm)
14238 ± 20 (362 nm)
27222 ± 192 (244 nm)

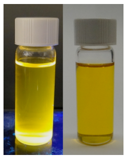

Solutions of **3e** in CH_2Cl_2 (0.4 mM) under ambient light (right) and irradiated at 365 nm with a UV lamp (left)

Absorption, emission, and excitation spectra of **5i** at 24 ˚C in CH_2Cl_2/TFA.

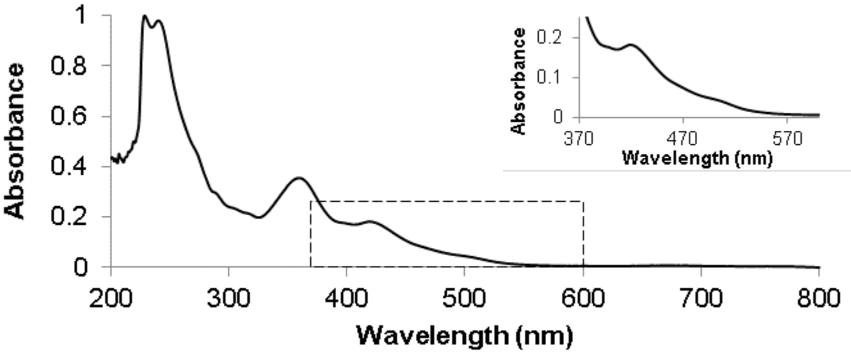

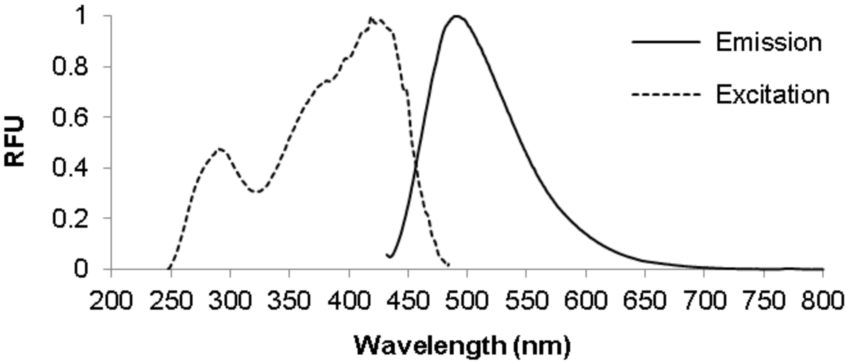

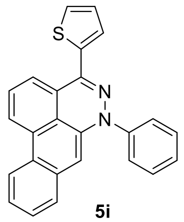

5i

Solvent: CH_2Cl_2/TFA
Quantum Yield (Φ): 1.5 %
Extinction Coefficient (ε):
6395 ± 318 (419 nm)
9607 ± 879 (360 nm)
31340 ± 27 (242 nm)
38234 ± 959 (228 nm)

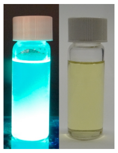

Solutions of **3e** in CH_2Cl_2/TFA (0.4 mM) under ambient light (right) and irradiated at 365 nm with a UV lamp (left)

Absorption, emission, and excitation spectra of **5j** at 24 ˚C in CH_2Cl_2.

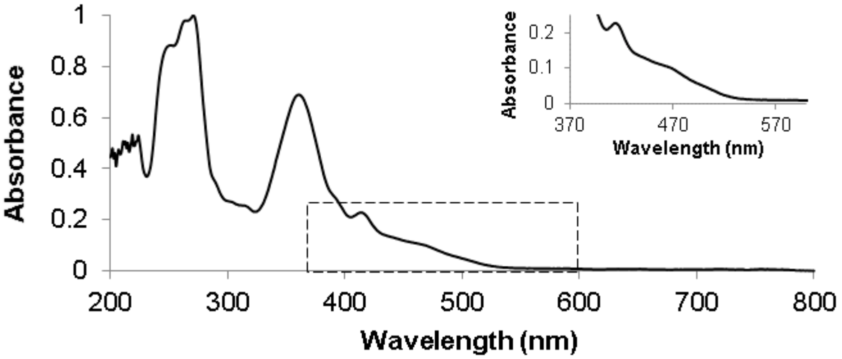

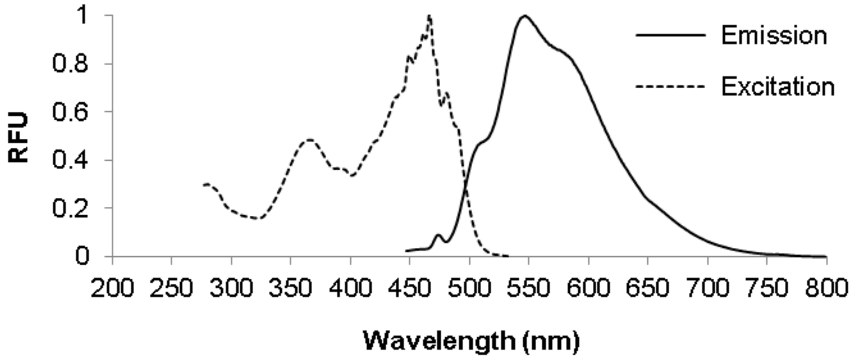

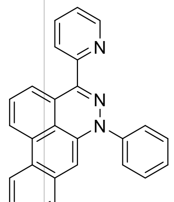

5j

Solvent: CH_2Cl_2
Quantum Yield (Φ): 0.6 %
Extinction Coefficient (ε):
1641 ± 46 (413 nm)
5306 ± 80 (361 nm)
17336 ± 601 (271 nm)

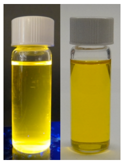

Solutions of **3e** in CH_2Cl_2 (0.4 mM) under ambient light (right) and irradiated at 365 nm with a UV lamp (left)

Absorption spectrum of **5j** at 24 ˚C in CH_2Cl_2/TFA.

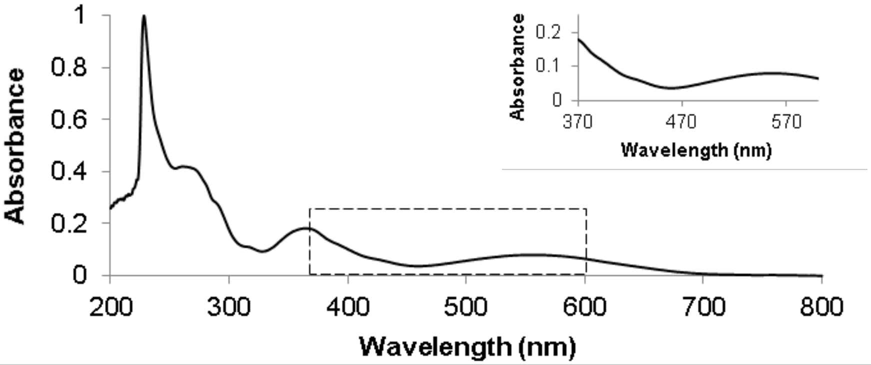

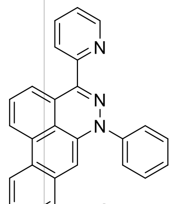

5j

Solvent: CH_2Cl_2/TFA
Quantum Yield (Φ): 0.0 %
Extinction Coefficient (ε):
1749 ± 100 (560 nm)
3857 ± 118 (365 nm)
26933 ± 133 (228 nm)

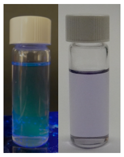

Solutions of **3e** in CH_2Cl_2/TFA (0.4 mM) under ambient light (right) and irradiated at 365 nm with a UV lamp (left)

Absorption, emission, and excitation spectra of **5q** at 24 ˚C in CH_2Cl_2.

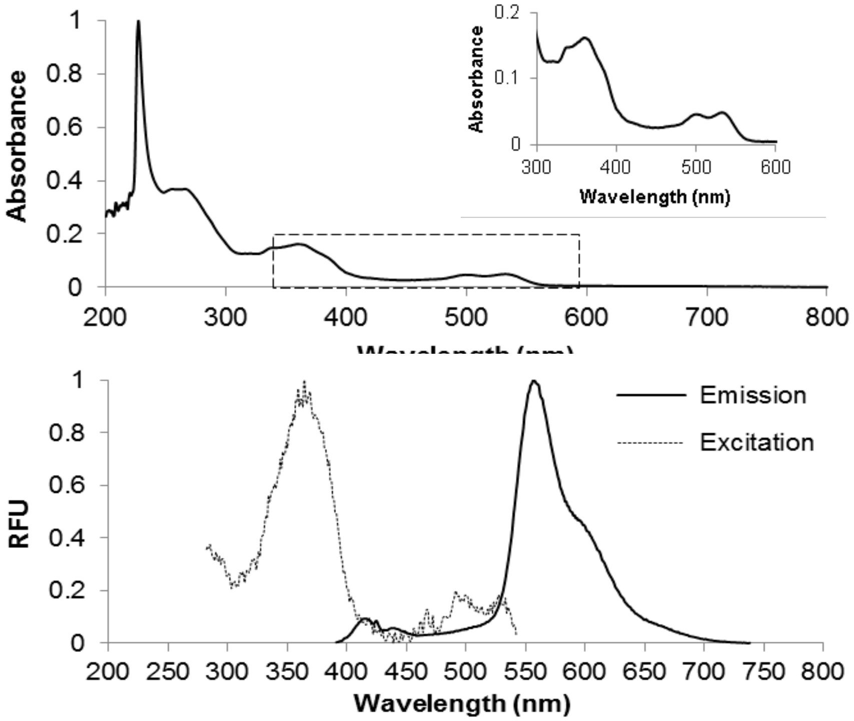

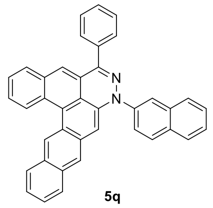

5q

Solvent: CH_2Cl_2
Quantum Yield (Φ): 6.7 %
Extinction Coefficient (ε):
2061 ± 339 (532 nm)
1850 ± 355 (501 nm)
9469 ± 57 (362 nm)
8663 ± 100 (340 nm)
23217 ± 698 (266 nm)
23141 ± 720 (257 nm)

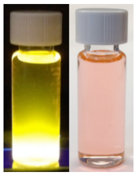

Solutions of **3i** in CH_2Cl_2 (0.4 mM) under ambient light (right) and irradiated at 365 nm with a UV lamp (left)

Absorption, emission, and excitation spectrum of **5q** at 24 ˚C in CH_2Cl_2/TFA.

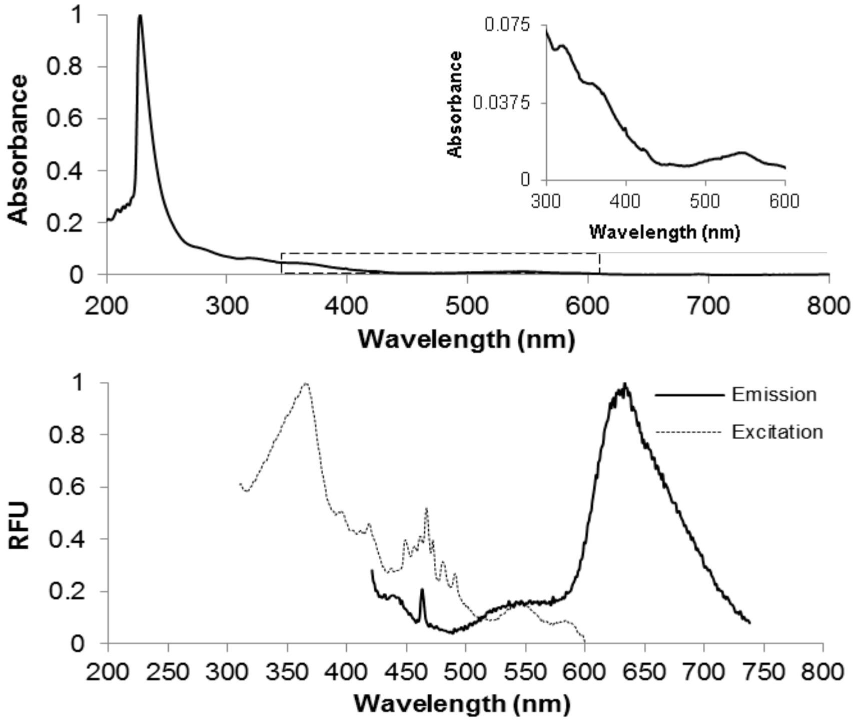

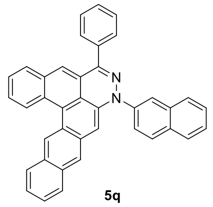

5q

Solvent: CH_2Cl_2/TFA
Quantum Yield (Φ): 2.0 %
Extinction Coefficient (ε):
2290 ± 519 (524 nm)
15500 ± 793 (279 nm)

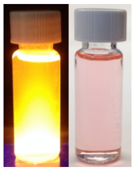

Solutions of **3i** in CH_2Cl_2/TFA (0.4 mM) under ambient light (right) and irradiated at 365 nm with a UV lamp (left)

Crystal data and structure refinement for **5a**.

Empirical formula	$C_{22}H_{16}N_2$
Formula weight	308.37
Temperature	100(1) K
Wavelength	0.71073 Å
Crystal system	monoclinic
Space group	$P2_1/c$
Cell constants:	
a	11.9993(9) Å
b	4.2453(3) Å
c	29.803(2) Å
b	100.261(4)°
Volume	1493.90(18) Å^3
Z	4
Density (calculated)	1.371 Mg/m^3
Absorption coefficient	0.081 mm^{-1}
F(000)	648
Crystal size	0.48 x 0.12 x 0.04 mm^3
Theta range for data collection	1.39 to 25.38°
Index ranges	-14 ≤ h ≤ 14, -5 ≤ k ≤ 5, -35 ≤ l ≤ 35
Reflections collected	32407
Independent reflections	2740 [R(int) = 0.0637]
Completeness to theta = 25.38°	99.9 %
Absorption correction	Semi-empirical from equivalents
Max. and min. transmission	0.7452 and 0.4717
Refinement method	Full-matrix least-squares on F^2
Data / restraints / parameters	2740 / 0 / 219

Goodness-of-fit on F^2	1.104
Final R indices [I>2sigma(I)]	R1 = 0.0541, wR2 = 0.1464
R indices (all data)	R1 = 0.0664, wR2 = 0.1579
Largest diff. peak and hole	0.308 and -0.270 e.Å^{-3}
